# Antibacterial, Antifungal, and Cytotoxic Effects of Endophytic *Streptomyces* Species Isolated from the Himalayan Regions of Nepal and Their Metabolite Study

**DOI:** 10.3390/biomedicines12102192

**Published:** 2024-09-26

**Authors:** Ram Prabodh Yadav, Chen Huo, Rabin Budhathoki, Padamlal Budthapa, Bibek Raj Bhattarai, Monika Rana, Ki Hyun Kim, Niranjan Parajuli

**Affiliations:** 1 Central Department of Chemistry, Tribhuvan University, Kirtipur 44618, Nepal; ramprabodh30@gmail.com (R.P.Y.); rabin.bc.992@gmail.com (R.B.); padambudthapa8@gmail.com (P.B.); 2 School of Pharmacy, Sungkyunkwan University, Suwon 16419, Republic of Korea; huochen.13@gmail.com; 3 Department of Chemistry and Biochemistry, Texas Tech University, Lubbock, TX 79409-1061, USA; rajbibek9990@gmail.com; 4 Central Department of Biotechnology, Tribhuvan University, Kirtipur 44618, Nepal; akinom949@gmail.com

**Keywords:** antibacterial effect, antifungal effect, cytotoxic effect, mass spectrometry, molecular networking, *Streptomyces*

## Abstract

**Background/Objectives:** Recently, antimicrobial-resistant pathogens and cancers have emerged as serious global health problems, highlighting the immediate need for novel therapeutics. Consequently, we aimed to isolate and characterize endophytic *Streptomyces* strains from the rhizospheres of the Himalayan region of Nepal and identify specialized metabolites with antibacterial, antifungal, and cytotoxic potential. **Methods:** To isolate *Streptomyces* sp., we collected two soil samples and cultured them on an ISP4 medium after pretreatment. We isolated and identified the strains PY108 and PY109 using a combination of morphological observations and 16S rRNA gene sequencing. **Results:** The BLAST results showed that PY108 and PY109 resembled *Streptomyces hundungensis* PSB170 and *Streptomyces* sp. Ed-065 with 99.28% and 99.36% nucleotide similarity, respectively. Antibacterial assays of ethyl acetate (EA) extracts from both isolates PY108 and PY109 in a tryptic soy broth (TSB) medium were conducted against four pathogenic bacteria. They showed significant antibacterial potential against *Staphylococcus aureus* and *Klebsiella pneumoniae*. Similarly, these extracts exhibited moderate antifungal activities against *Saccharomyces cerevisiae* and *Aspergillus niger*. Cytotoxicity assays on cervical cancer cells (HeLa) and breast cancer cells (MCF-7) revealed significant potential for both extracts. LC-MS/MS profiling of the EA extracts identified 27 specialized metabolites, including diketopiperazine derivatives, aureolic acid derivatives such as chromomycin A, and lipopeptide derivatives. In comparison, GC-MS analysis detected 34 metabolites, including actinomycin D and γ-sitosterol. Furthermore, a global natural product social molecular networking (GNPS)-based molecular networking analysis dereplicated 24 metabolites in both extracts. **Conclusions:** These findings underscore the potential of endophytic *Streptomyces* sp. PY108 and PY109 to develop new therapeutics in the future.

## 1. Introduction

Antimicrobial resistance (AMR) presents a significant challenge to global health, with a WHO report predicting 10 million deaths per year by 2050 due to the excessive and improper use of antibiotics [1]. In 2019, an estimated 1.27 million fatalities were attributed to drug-resistant bacterial infections [2,3]. The rise of multidrug-resistant bacteria, or ‘superbugs’, is exacerbated by factors such as antibiotic misuse, poor sanitation, self-treatment, and inadequate surveillance, outpacing the discovery of new drugs. Concurrently, the WHO’s International Cancer Research Agency (IARC) has highlighted stark disparities in cancer care access and the increasing global cancer burden, with 9.7 million cancer-related deaths and over 20 million new cases reported in 2022 [4]. These findings reveal significant gaps between high- and low-income countries, with the former more likely to offer basic cancer services within health systems. Projections suggest a 77% increase in new cancer cases by 2050, underscoring the urgent need for equitable access to cancer prevention, diagnosis, and treatment worldwide [5]. This urgency increases with the potential rise in figures if effective medications are not developed, prompting researchers to focus intensely on developing new therapeutics to combat cancers [6] and AMR [7].

Over 60% of current cancer drugs originate from natural products [8], highlighting their pivotal role in ongoing drug discovery efforts. Microorganisms represent a vast and largely untapped natural resource, offering diverse chemical structures that could serve as promising anticancer drug candidates [9]. Researchers are exploring the microbial world to identify unique metabolites that are effective against cancers and MDR bacteria. *Streptomyces*, a genus of Gram-positive bacteria, is renowned for producing a broad spectrum of therapeutics. Endophytic *Streptomyces*, though understudied, is a valuable source of bioactive secondary metabolites. These endophytic microbes, primarily found in rhizospheres, produce bioactive metabolites driven by the need to compete for resources and defend against pathogens. Such metabolites may inhibit the growth of competing microorganisms, enhance plant defense mechanisms, foster symbiotic relationships, and ultimately support the survival of both the microbes and their host plants [10]. Research has shown that endophytic relationships encourage the evolution of the biosynthetic gene clusters (BGCs) responsible for producing these secondary metabolites, thus making them valuable in drug discovery and development [11]. Metabolites produced by endophytic *Streptomyces*, such as misamycin [12], 5,7-dimethoxy-4-p-methoxylphenylcoumarin [13], plicacetin [14], endostemonines A–J [15], and actinomycins [16], have demonstrated anticancer, antifungal, antibacterial, insecticidal, and antimicrobial activities, respectively.

Currently, liquid chromatography–tandem mass spectrometry (LC-MS/MS) and gas chromatography–mass spectrometry (GC-MS) are the most widely used hyphenated techniques for examining specialized metabolites produced by natural products. Complementing these methods, other bioinformatics tools have been developed, providing additional valuable insights into metabolites in natural product research. One such tool is molecular networking (MN) based on global natural product social molecular networking (GNPS), which has recently been utilized in drug discovery programs to organize and visualize MS/MS data [17,18].

This research focuses on the identification of endophytic *Streptomyces* species from plant rhizospheres, conducting antibacterial, antifungal, and cytotoxicity assays, followed by the annotation of metabolites using cutting-edge techniques, such as GC-MS, LC-MS/MS, and GNPS-based MN. Accordingly, we have collected two rhizospheric samples, PY108 and PY109, from the Himalayan regions of Nepal, situated at elevations of 4150 m and 3750 m above sea level, respectively. These regions are known for their symbiotic relationships between bacteria and the rhizosphere.

## 2. Materials and Methods

### 2.1. Source of Microbes

Two soil samples, labeled PY108 and PY109, were collected from the rhizospheres of plants in a forest. Sample PY108 was collected from Tilicho Base Camp at an altitude of 4150 m (coordinates: 28.69167° N, 83.85278° E), while PY109 was collected from Khangsar at 3750 m altitude (coordinates: 28.6996° N, 83.9109° E), both in the Himalayan regions of the Manang district in Nepal. After carefully removing the top 3 cm of surface soil, the samples were collected from a depth of up to 15 cm using sterile polyethylene bags. These bags were then sealed to prevent contamination and stored at 4 °C for optimal preservation [19].

### 2.2. Isolation of Streptomyces Species

Soil samples were pretreated with physicochemical methods to eliminate common unwanted microbes, such as Gram-negative bacteria and fungi. To achieve this, 1 g of each soil sample was suspended in 100 mL of saline water (8.5 g/L) and incubated on a shaker at 28 °C for 30 min [20]. After pretreatment, the soil suspensions were thoroughly vortexed. Subsequently, serial dilutions were performed in a three-fold manner, and 100 μL of each dilution was spread onto International *Streptomyces* Project 4 (ISP4) medium plates. These plates were supplemented with nalidixic acid (20 mg/L) and cycloheximide (50 mg/L), and incubated for 5–7 days at 28 °C. Colonies that emerged were selected for further identification based on their morphology, mycelium, and color, followed by Gram staining to confirm the bacterial identity [21].

The inhibitory metabolite-producing capability of the two isolates was assessed using the cross-streak method against *Staphylococcus aureus* and *Escherichia coli*. The isolates were streaked on Mueller–Hinton agar (MHA) and incubated at 28 °C for 3–4 days. Subsequently, the test pathogens were streaked perpendicularly and incubated at 37 °C for 24–48 h. The inhibitory activity was indicated by a zone of bacterial inhibition around the isolate’s growth [22].

A detailed morphological assessment was also conducted, using both macroscopic observation and the naked eye. Colony characteristics, such as shape, size, and color, as well as pigment production and the development of aerial and substrate mycelia, were examined according to Bergey’s Manual of Systematic Bacteriology [23]. Furthermore, microscopic examination using Gram staining provided insights into the arrangement of spores and the structures responsible for their formation.

### 2.3. Molecular Identification

*Streptomyces* strains were cultured in 25 mL TSB medium (tryptone 17.0 g, soytone 3.0 g, glucose 2.5 g, sodium chloride 5.0 g, dipotassium phosphate 2.5 g, pH 7.3 ± 0.2 at 28 °C; volume 1 L water) broth and incubated at 28 °C for 4–5 days. The cultures were then centrifuged, and the pellets were treated with a lysis buffer and lysozyme, followed by incubation for lysis. The lysate was further processed by adding EDTA, proteinase K, and SDS, after which the genomic DNA was precipitated using isopropanol and ethanol. The DNA pellets were washed, dried, and resuspended in a TE buffer for analysis via 0.4% agarose gel electrophoresis (Cleaver Scientific, Rugby, UK), and visualized under a Gel Doc system (UVITEC, Cambridge, UK).

The 16S rRNA gene amplification involved a PCR reaction (Thermo Fisher Scientific, Waltham, MA, USA), consisting of nuclease-free water, 5x PCR premix (GenoTech Corporation, Daejeon, Republic of Korea), 0.3 µL of both forward and reverse primers at 100 pM concentration, and 0.4 µL of template DNA, producing a final volume of 10 µL. A universal primer set (27F: 5′-AGAGTTTGATCCTGGCTCAG-3′ and 1492R: 5′-GGTTACCTTGTTACGACTT-3′) was used. The PCR protocol included an initial denaturation at 98 °C for 5 min, followed by 29 cycles of denaturation at 98 °C for 10 s, annealing at 54 °C for 10 s, and extension at 72 °C for 2 min. A final extension was conducted at 72 °C for 7 min, with the samples then held indefinitely at 4 °C. The PCR product was purified using the QIAquick Gel Extraction Kit (Qiagen, Germantown, MD, USA) and confirmed using electrophoresis. Sequencing was performed by the GenoTech Corporation, using the Sanger dideoxy method.

The phylogenetic identity of the actinomycete was initially established by querying GenBank using the BLAST program on NCBI to retrieve FASTA format files (http://www.ncbi.nlm.nih.gov/blast/), accessed on 22 July 2024 followed by confirmation through multiple sequence alignment to visually inspect the genus-specific nucleotide signatures and the construction of phylogenetic trees. These analyses were conducted using the MEGA 11 software package MEGA Software (version 11.0.13) (https://www.megasoftware.net/), accessed on 10 June 2024 [24], with multiple sequence alignment and tree construction utilizing the neighbor-joining method within the Cluster W package.

### 2.4. Shake Flask Fermentation

To maximize the production of secondary metabolites, the identified isolates underwent a submerged-state fermentation process. Each isolate was cultured in a conical flask containing 100 mL of TSB and incubated at 28 °C with constant shaking at 180 rpm for 7–10 days [25]. This temperature and aeration were optimized to facilitate robust growth and metabolite production. Successful fermentation was indicated by visible signs, such as pellet formation, clumping, or increased turbidity. Following fermentation, the broth was separated using a separating funnel after adding an equal volume of ethyl acetate (EA) to extract the secondary metabolites. This EA extract then served as the basis for future investigations, including the screening and purification of specific antimicrobial compounds [26].

### 2.5. Antibacterial Assays

The antibacterial activities of the EA extracts from *Streptomyces* **sp.** PY108 and PY109 were assessed against *Staphylococcus aureus* ATCC 43300, *Escherichia coli* ATCC 25922, *Klebsiella pneumoniae* ATCC 700603, and *Shigella sonnei* ATCC 25931 using the agar well diffusion method [27]. The test organisms were swabbed onto Mueller–Hinton agar (MHA) plates and incubated at 37 °C for 24 h in Mueller–Hinton broth (MHB) to maintain a turbidity of 0.5 McFarland standard (1.5 × 10^8^ CFU/mL). The EA extracts (in 50% DMSO), a positive control (1 mg/mL neomycin), and a negative control (50% DMSO) were added to 6 mm diameter wells created using sterile cork borers. The plates were incubated at 37 °C for 24 h, after which the zones of inhibition (ZOIs) were measured.

The minimum inhibitory concentration (MIC) and minimum bactericidal concentration (MBC) values were determined using the broth dilution method, following the Clinical Laboratory Standards Institute (CLSI) guidelines [28]. Two-fold dilutions of the extracts were prepared in sterile 96-well plates with MHB. Bacterial suspensions were adjusted to 1.5 × 10^8^ CFU/mL by diluting 1:100 to match the 0.5 McFarland standard in MHB, and 10 μL was added to each well, except for the negative control. The plates were incubated at 37 °C for 18 h, followed by the addition of 5 μL of resazurin and an additional 3 h of incubation to determine MIC by observing color changes. To determine MBC, aliquots from the well containing the MIC value and the next four higher concentrations were plated onto nutrient agar (NA) and incubated at 37 °C overnight; MBC was identified as the lowest concentration that showed no visible bacterial growth.

### 2.6. Antifungal Assay

The antifungal potency of the EA extracts from fermented *Streptomyces* sp. PY108 and PY109 was assessed on potato dextrose agar (PDA) using the agar well diffusion method against two fungal strains: *Saccharomyces cerevisiae* and *Aspergillus niger* [29]. Initially, the fungal strains were spread onto PDA plates, followed by the creation of 6 mm diameter wells using a sterile cork borer at four symmetrical points on each plate. Each well received 80 µL of the bacterial extract solution (2.1 mg/mL in DMSO). Cycloheximide at 1 mg/mL served as the positive control, while the plates with untreated fungi functioned as the negative control. After allowing 1 h at room temperature for diffusion, the plates were inverted and incubated at 28 °C for 24 to 72 h. The zones of inhibition (ZOIs) around each well were then measured to evaluate the antifungal activity.

### 2.7. Cytotoxicity Assay

The cytotoxic effects of the EA extracts from *Streptomyces* sp. PY108 and PY109 were evaluated using the MTT assay on the MCF-7 breast cancer and HeLa cell lines, following the NCIB guidelines [30,31]. HeLa cells were sourced from PGIMER, Chandigarh, and MCF-7 cells from Shikhar Biotech, Lalitpur. The cells were cultured in DMEM (GIBCO Laboratories, Green Island, NY, USA) supplemented with 10% FBS, 7.5% sodium bicarbonate, 200 mM L-glutamine, 100 mM sodium pyruvate, and 1% penicillin–streptomycin. Additionally, the medium for MCF-7 included 1% non-essential amino acids (NEAAs). The cells were maintained at 37 °C in a humidified atmosphere containing 5% CO_2_. At 80% confluency, the cells were detached using 0.5% trypsin-EDTA (HiMedia Laboratories, Mumbai, India), counted, and seeded at a density of 3.5 × 10^3^ cells/well for HeLa and 4 × 10^3^ cells/well for MCF-7 in 96-well plates. These plates were incubated for 16 h to allow for cell attachment.

The EA extract stock solutions were prepared in DMSO to final concentrations of 0, 5, 10, 20, and 50 μg/mL, with 0.5% DMSO serving as the negative control. After 16 h, the old media was replaced with fresh media containing varying concentrations of extracts, and the cells were incubated for an additional 72 h. Subsequently, a 5 mg/mL MTT solution (HiMedia Laboratories, Mumbai, India) in 1X PBS (Thermo Fisher Scientific Inc., Waltham, MA, USA) was filtered and added to each well. After 3 h of incubation, the mitochondrial enzymes in the living cells reduced the MTT to purple formazan crystals, indicating the number of viable cells. These crystals were dissolved in DMSO, and the absorbance was measured at 570 nm using a microplate reader (BMG Lab, Ortenberg, Germany). The cytotoxicity was quantified by calculating the IC_50_ value, which represents the concentration required to inhibit the cell viability by 50%. This was determined using the formula: IC_50_ = (0.5 − C)/M, where ‘M’ is the slope of the dose–response curve at the 50% inhibition threshold, and ‘C’ is the response (inhibition percentage) at a given inhibitor concentration.

### 2.8. Liquid Chromatography-Mass Spectrometric Analysis

The LC-HRMS/MS analysis of the EA extracts was conducted using an Agilent G6545B quadrupole time-of-flight (Q-TOF) mass spectrometer (Agilent Technologies, Santa Clara, CA, USA) equipped with a heated electrospray ion source. The analysis was performed at Sungkyunkwan University, Suwon, Republic of Korea, operating in positive ion mode. For the MS/MS analysis, the two extracts were prepared by dissolving in HPLC-grade acetonitrile and water at a concentration of 1 mg/mL. Subsequently, 150 μL of each sample was transferred to HPLC autosampler vials. Chromatographic separation was achieved on an Acquity^®^ UPLC BEH reverse-phase C18 column (150 mm × 2.1 mm, 1.7 µm). The mobile phase comprised 0.1% formic acid in water (A) and acetonitrile (B). The gradient was set as follows: starting with 5% B for the first 2 min, increasing to 20% B over the next 3 min, ramping up to 100% B from minutes 5 to 20, maintaining 100% B from minutes 20 to 23, and then returning to 5% B by minute 25. The flow rate was maintained at 0.3 mL/min, and the injection volume was 3 µL. The ionization source parameters were set with a cone voltage of 40 V and a capillary voltage of 2.5 kV. MS data acquisition covered a mass range from 50 to 1700 Da in a positive ionization mode.

### 2.9. GNPS-Based Molecular Networking

The LC-HRMS/MS data were converted from ‘.d’ to ‘.mzXML’ format using the open-source MSConvert software (version 3.0), available through ProteoWizard (https://proteowizard.sourceforge.io/), accessed on 25 January 2024. The files were uploaded to the GNPS platform using the recommended FTP client, WinSCP. The visualization of the MS/MS data adhered to established GNPS-based procedures, accessed on 29 January 2024.

For constructing the molecular network, both the precursor and fragment ion mass tolerances were set at 0.02 Da. The advanced network settings were configured as follows: a minimum pairs cosine of 0.6, a network TopK of 10, a maximum connected component size of 100, a maximum of 3 matched fragment ions, and a minimum cluster size of 2. For the library search, the criteria included a minimum of three matching peaks with a score threshold of 0.6. All the other parameters were maintained at their default values. The molecular networks generated on GNPS were then exported to Cytoscape (version 3.9.1) in the ‘.graphml’ format, facilitating customized visualization and further analysis.

### 2.10. Gas Chromatography–Mass Spectrometric Analysis

The EA extract of *Streptomyces* sp. PY109 was analyzed using an Agilent 8890 GC system coupled with a Single Quadrupole Mass Spectrometer 5977B MSD (Agilent Technologies, Santa Clara, CA, USA) at the SAIF, Indian Institute of Technology Madras (IITM). Similarly, the GC-MS analysis of the EA extract from *Streptomyces* sp. PY108 was conducted at the SRM Institute of Science & Technology in Chennai, India, using an identical model of the instrument. Both analyses utilized an Agilent HP-5 MS UI column (30 m × 250 µm × 0.25 µm) and were run for a total duration of 53.5 min. The temperature program for the GC oven started at 75 °C and increased to 350 °C at a rate of 5 °C per minute. The samples were injected using a 10 µL syringe with a 1 µL injection volume. The rear injector was set with a split flow rate of 18 mL/min. MS detection was conducted with a 70-eV electron ionization source, scanning from 50 to 600 Da over 1.5 min.

### 2.11. Metabolomics Data Analysis

In this study, we used the open-source MSConvert software (version 3.0) to convert the raw LC-HRMS/MS data from the Waters instruments into the .mzXML format for *Streptomyces* sp. PY108 and PY109. The converted data were then processed using the MestReNova software (version 12.0.0), where parameters such as the exact mass, observed mass, absolute error, ring double bond equivalent (RDBE), and molecular formulae were generated and analyzed. These parameters were further verified and validated using the SIRIUS 5.7.2 software.

The metabolites annotated were validated based on the SIRIUS score (CSI: FingerID similarity %), literature reviews, and searches in natural product-based databases and servers, including PubChem (https://pubchem.ncbi.nlm.nih.gov/, accessed 14–28 March 2024), ChemSpider (https://www.chemspider.com/, accessed 14–28 March 2024), Natural Products Atlas (https://www.npatlas.org/, accessed 14–28 March 2024), and the LOTUS database (https://lotus.naturalproducts.net/, accessed 14–28 March 2024).

For the GC-MS data, we used the Open Lab CDS software (version 2.5) for processing, with compound identification conducted via the NIST (2017) Mass Spectral Library search. Additionally, the .mzXML data were analyzed using GNPS for the visualization of MS/MS data and annotation of natural compounds.

## 3. Results

### 3.1. Isolation and Morphological Characterization of Isolates

Two endophytic microbes, PY108 and PY109, were isolated from soil samples collected in Manang, Nepal, using an ISP4 medium supplemented with nalidixic acid (20 mg/L) and cycloheximide (50 mg/L). After seven days of incubation, the colonies displayed typical *Streptomyces* characteristics—rough, tough, dry, and elevated—as depicted in Figure S1, and were selected for further study. Gram staining of these strains revealed filamentous bacteria that retained a persistent violet color, indicative of Gram-positive bacteria. Microscopic observation under 100x oil immersion revealed hair-thread-like structures, suggesting mycelial formation. Upon streaking on the ISP4 medium, the strains PY108 and PY109 sporulated within 3–5 days. The colony morphology, substrate (white, red, and yellowish), and aerial colors, as well as pigment production, closely matched the descriptions for actinomycetes in Bergey’s Manual of Systematic Bacteriology.

### 3.2. Molecular Characterization and Phylogenetic Analysis

Genomic DNA was isolated using the phenol–chloroform method as described by Green et al. [32] (Figure S2) and was used as a template for the amplification of the 16S rRNA gene using universal primers (27F and 1492R). PCR amplification in a thermocycler produced the expected 1.5 kb products, corresponding to the 16S rRNA gene, confirmed by comparison with a 1 kb DNA ladder (Figure S3). BLAST analysis revealed that the isolates PY108 and PY109 showed a high nucleotide sequence similarity with the *Streptomyces hundungensis* PSB170 strain (99.28%) and *Streptomyces* sp. Ed-065 strain (99.36%), respectively. These results confirm that the actinomycetes, PY108 and PY109, belong to the genus *Streptomyces.* The sequences have been made available in GenBank under accession the numbers PP386434 and PP379912, respectively. A phylogenetic tree (Figure S4) illustrates the evolutionary relationships of *Streptomyces* sp. PY108 and PY109 to their closest identified taxa.

### 3.3. Antibacterial Assays of Streptomyces Isolates

EA extracts from the fermentation broth of *Streptomyces* sp. PY108 and *Streptomyces* sp. PY109 were tested for antibacterial activity, as shown in Figure S5, using the agar well diffusion method. Table S1 displays a significant zone of inhibition (ZoI) observed against *Escherichia coli*, *Klebsiella pneumoniae*, *Shigella sonnei*, and *Staphylococcus aureus*. These extracts produced ZoIs against the tested pathogens that were comparable with the positive control, neomycin, suggesting the presence of therapeutic agents in the bacterial extracts.

**Figure 1:**
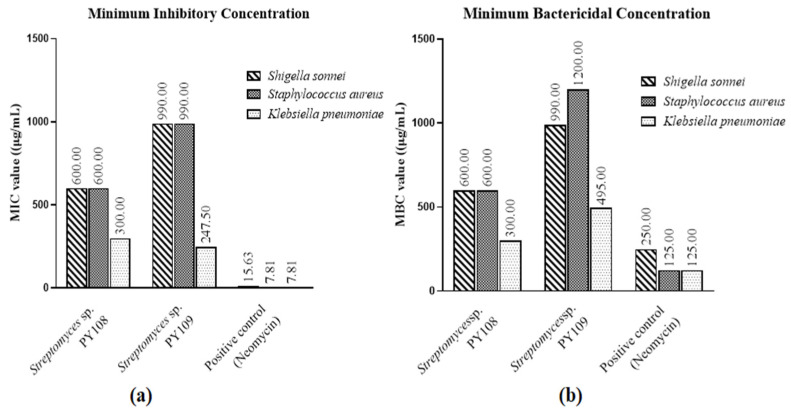
(**a**) MIC values and (**b**) MBC values for EA extracts of *Streptomyces* sp. PY108 and PY109 against tested bacterial strains.

According to the MIC and MBC tests (Figures S6 and S7), EA extracts of *Streptomyces* sp. PY108 and PY109 exhibited substantial antibacterial activity against *S. aureus*, *K. pneumoniae*, and *S. sonnei*, as shown in Table S2. Neomycin, used as the positive control, was employed at concentrations ranging from 500 to 250 μg/mL in two-fold dilutions. Compared with *S. aureus* and *S. sonnei*, Figure 1 illustrates the potency of the EA extracts of both isolates against different bacteria. These results suggest that the extracts can be further investigated for possible therapeutic uses and have the potential to serve as antibacterial agents.

### 3.4. Antifungal Potency of Streptomyces Isolates

**Figure 2:**
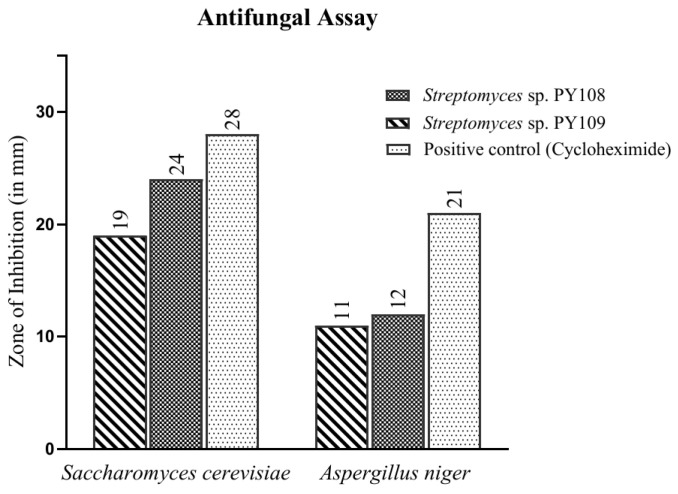
Antifungal activity of EA extracts of *Streptomyces* sp. PY108 and PY109.

In this study, the agar well diffusion method was employed to test the antifungal activity of EA extracts obtained from the fermentation of *Streptomyces* sp. PY108 and PY109 against *Saccharomyces cerevisiae* and *Aspergillus niger*. The observed results are depicted in Figure 2 and Table S3. Cycloheximide, used as a positive control, showed a zone of inhibition (ZoI) of 28 mm against *S. cerevisiae*. It was found that *Streptomyces* sp. PY108 (Figure S8) demonstrated a ZoI of 24 mm, which is the highest inhibition against *S. cerevisiae*, compared with *Streptomyces* sp. PY109, which showed a ZoI of 19 mm. Thus, the EA extract of *Streptomyces* sp. PY108 exhibited notable antifungal potential.

However, moderate antifungal activities were demonstrated against *A. niger* mycelium by EA extracts from both *Streptomyces* species, as depicted in Figure S9. Cycloheximide, used as a positive control, showed a zone of inhibition (ZoI) of 21 mm against *A. niger*. In comparison, the ZoIs observed for *Streptomyces* sp. PY108 and PY109 were 12 mm and 11 mm, respectively, which were significantly lower than that of cycloheximide. Thus, it can be inferred that the EA extract of *Streptomyces* sp. PY108 should be further explored for therapeutic applications as it exhibited better activity.

### 3.5. Cytotoxicity Assay

**Figure 3:**
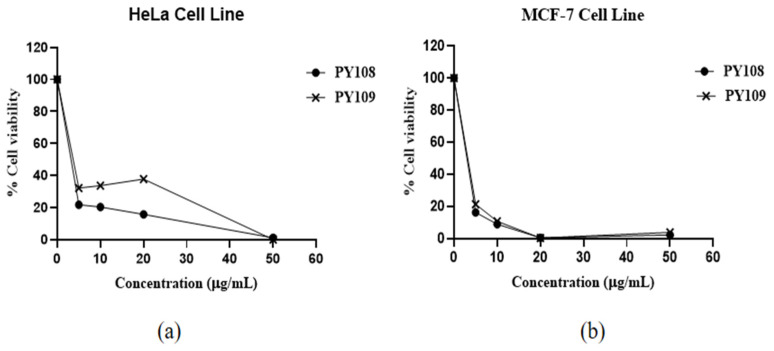
Cytotoxic activity of EA extracts from *Streptomyces* sp. PY108 and PY109 against (**a**) the HeLa cell line and (**b**) the MCF-7 cell line.

The results (Figure 3) showed that EA extracts from both *Streptomyces* species significantly inhibited both cell lines, with a dose-dependent response observed. The efficacy of these extracts is expressed through IC_50_ values, which indicate the concentration required to inhibit 50% of the cell viability. The EA extracts of *Streptomyces* sp. PY108 and *Streptomyces* sp. PY109 exhibited IC_50_ values of 4.534 μg/mL and 5.069 μg/mL against HeLa cells, respectively, indicating a slightly better potential of *Streptomyces* sp. PY108. For the MCF-7 cell lines, the IC_50_ values were 4.187 μg/mL and 4.253 μg/mL for the extracts of *Streptomyces* sp. PY108 and PY109, respectively.

An approximately 80% decrease in cell viability was observed in both cell lines at a 5 μg/mL concentration of the EA extracts from *Streptomyces* sp. PY109. Increasing the concentrations of the extracts led to a further reduction in cell viability. About an 80–85% reduction in cell viability was noted at concentrations below 5 μg/mL, while 100% cell death was observed at 50 μg/mL. These results indicate that the extract of *Streptomyces* sp. PY109 has a relatively higher cytotoxicity potential. However, these extracts exhibited greater toxicity against the MCF-7 cell lines compared with the HeLa cell line.

### 3.6. Liquid Chromatography–Mass Spectrometric Analysis

**Figure 4:**
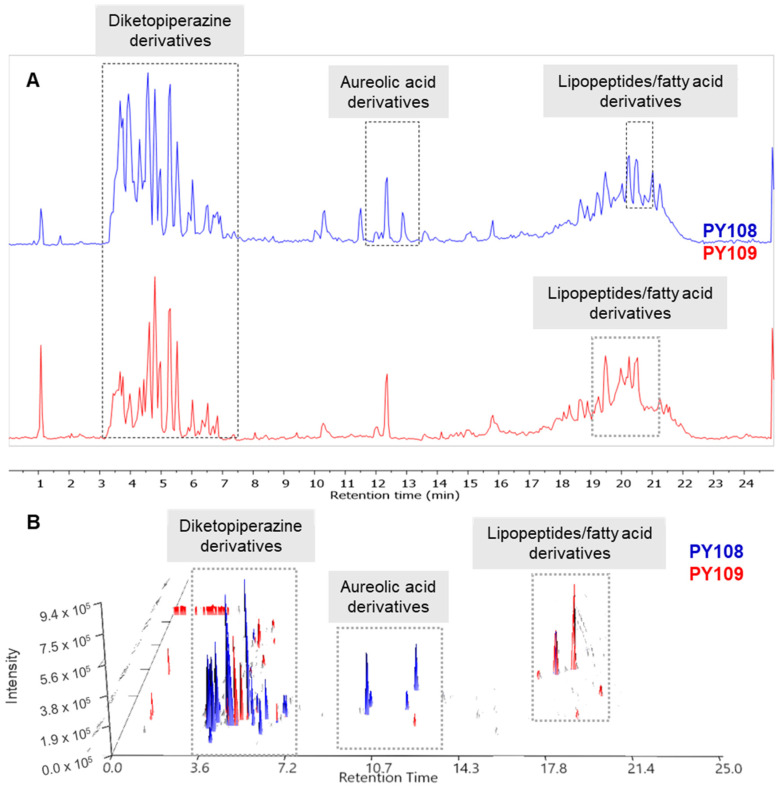
(**A**) Total ion chromatograms and (**B**) LC-MS 3D plots of the EA extracts of *Streptomyces* sp. PY108 and PY109 are presented in a stacked format.

The raw LC-MS/MS data generated from the mass spectrometry were processed and analyzed using the MestReNova software (version 12.0.0, Galicia, Spain) (accessed on 10–30 June 2024) available at the Mestre laboratory. The total ion chromatograms (TICs) of the EA extracts from the *Streptomyces* strains PY108 and PY109 were overlaid to assess the variations in metabolite profiles. Additionally, the LC-MS 3D plots from both samples were stacked together, as shown in Figure 4 allowing for a precise three-dimensional view of the complex metabolite composition in terms of retention time, *m*/*z* value, and peak intensity. The LC/MS data confirmed that *Streptomyces* sp. PY108 and PY109 exhibit similar chemical profiles, characterized by the production of diketopiperazine derivatives, aureolic acid derivatives, lipopeptides, and fatty acid derivatives, which vary according to their polarity.

In this study, we identified 27 metabolites across both samples belonging to various classes, such as diketopiperazines (e.g., cyclo-(D-Pro-L-Val), cyclo-(D-Phe-L-Pro)), aureolic acid derivatives (e.g., chromomycin A3, chromomycin A2, chromomycin A2-1, chromomycin A3-1), alkyl diethanolamine (*N*-lauryldiethanolamine), *N*-acyl amine derivatives (e.g., *N*-acetyltyramine), and lipopeptides (e.g., surfactin B, surfactin C). The majority of the metabolites were diketopiperazines. The secondary metabolites identified via the LC-HR MS/MS analyses are presented in Table 1 and Figure 5. Additionally, the base peak chromatograms (BPCs) and MS profiles that depict the identified metabolites are provided in Supplementary Figures S10–S36.

### 3.7. Global Natural Product Social Molecular Networking (GNPS) Analysis

**Figure 6:**
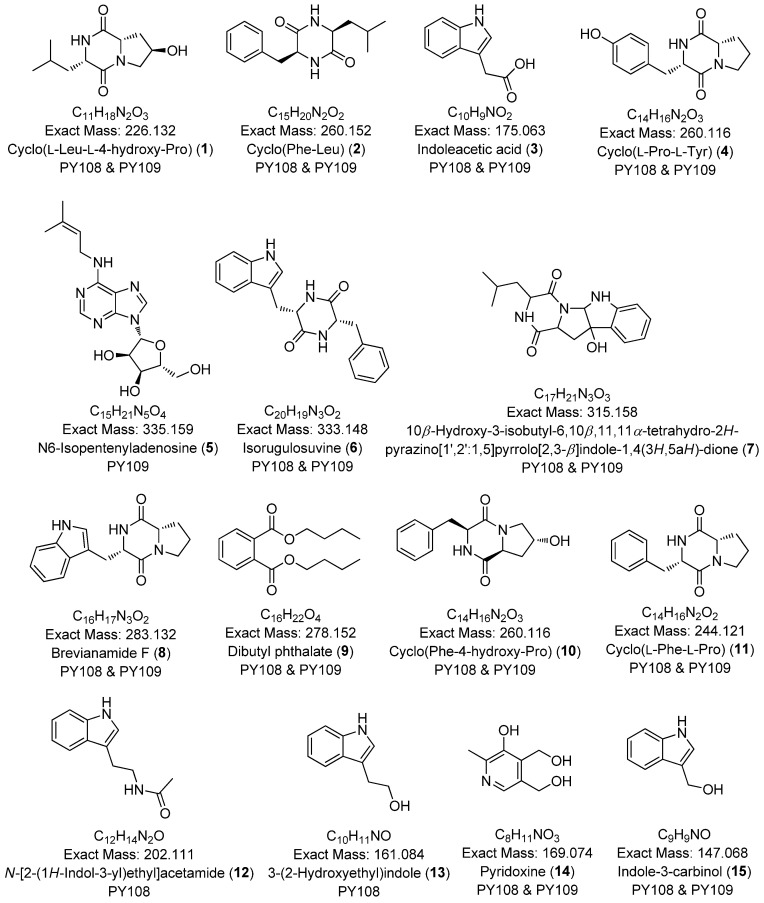
Annotated diketopiperazines derivatives from *Streptomyces* sp. PY108 and PY109 using a GNPS analysis.

**Figure 7:**
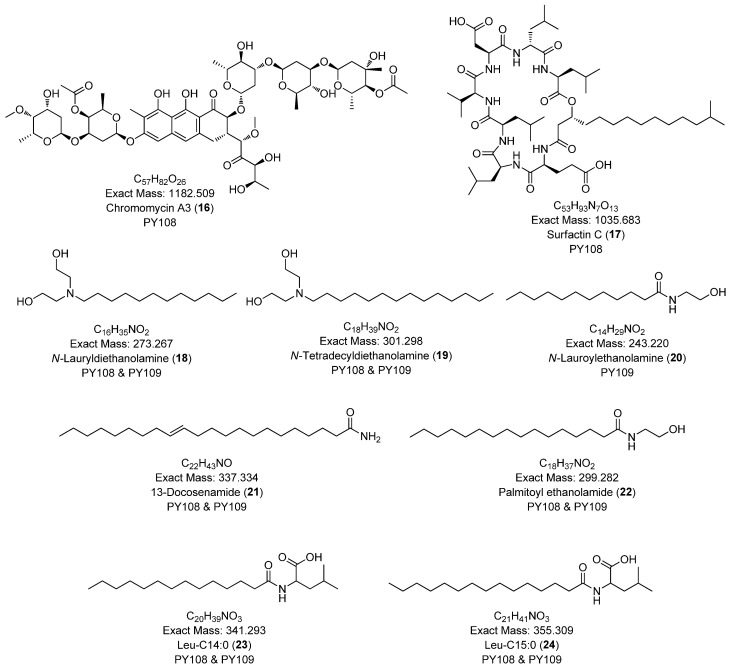
Annotated aureolic acid derivatives, lipopeptides, and *N*-acylethanolamines from *Streptomyces* sp. PY108 and PY109 using a GNPS analysis.

To comprehensively investigate the detailed metabolite profile of *Streptomyces* sp. PY108 and PY109, we conducted MS^2^ and GNPS metabolic profiling. A total of 362 molecular ions, which displayed the MS^2^ spectra in the two samples, were represented by nodes connected by 633 edges in the molecular network. We successfully identified and dereplicated 24 known compounds through the GNPS library with an error below 10, as listed in Table 2. The chemical structures of these compounds are illustrated in Figure 6 and Figure 7.

**Figure 8:**
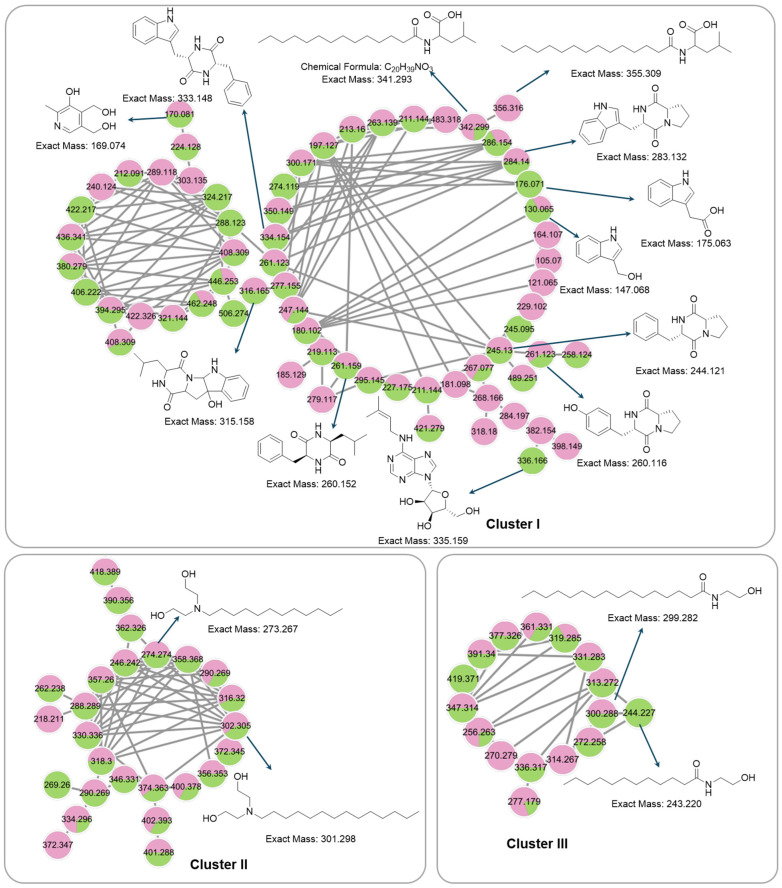
Cluster of annotated compounds putatively characterized by the molecular network obtained from the MS/MS data.

This analysis revealed a rich diversity of compounds produced by both *Streptomyces* species, with several compounds identified in both strains and unique compounds specific to each. In both PY108 and PY109, we identified derivatives of diketopiperazines, aureolic acids, lipopeptides, and *N*-acylethanolamines, as shown in Figure 6 and Figure 7. The presence of these common compounds suggests that PY108 and PY109 may possess similar biosynthetic pathways, potentially reflecting their shared ecological niches or evolutionary backgrounds. An MS/MS analysis was conducted using Cytoscape, as illustrated in Figure 8.

**Figure 9:**
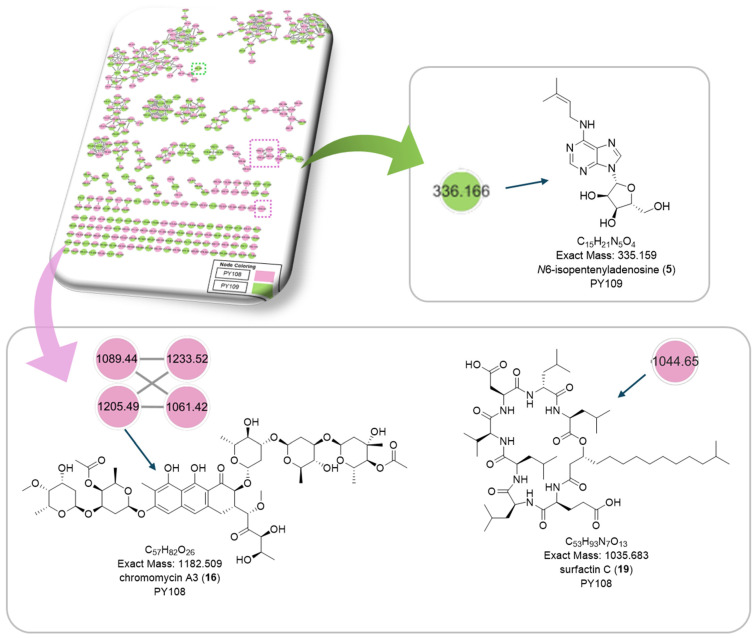
Chemical structures of specific compounds from *Streptomyces* sp. PY108 using GNPS analysis.

Additionally, we identified a nucleoside derivative, N6-Isopentenyladenosine (**5**), in PY109. The production of *N*_6_-Isopentenyladenosine (**5**) could suggest a role in plant-microbe interactions, potentially in enhancing plant growth and health through cytokinin-like activity. In PY108, we identified the antibiotic chromomycin A3 (**16**) and the lipopeptide surfactin C (**19**), indicating that PY108 may possess a potent antimicrobial arsenal that could provide a competitive advantage in natural environments (Figure 9).

### 3.8. Gas Chromatography–Mass Spectrometry Analysis

A GC-MS analysis was employed to investigate the volatile and non-polar chemical composition of bioactive extracts from *Streptomyces* sp. PY108 and PY109. The GC-MS chromatograms obtained from the EA extracts of these species are shown in Figures S37 and S38, respectively. A total of 34 compounds were identified in both samples by comparing the retention times, molecular weights, and molecular formulas of each component’s mass spectrum against the NIST library. The identified metabolites are listed in Table 3 and illustrated in Figure S39. The spectra of the volatile metabolites identified in the GC-MS analysis are presented in Figures S40–S72. Among the 34 identified compounds, ethyl iso-allocholate, ergotaman-3′,6′,18-trione, 2-propenoic acid, 1,9-dioxacyclohexadeca-4,13-diene-2,10-dione, 7,8,15,16-tetramethyl-, carda-16,20(22)-dienolide, and D-homo-24-nor-17-oxachola-20,22-dien-16-one emerged as the most significant.

## 4. Discussion

The increasing incidence of cancer and the rise of antibiotic-resistant bacteria highlight the urgent need to explore microbial natural products for new therapeutic agents. *Streptomyces* species, particularly from unexplored regions, show promise as sources of novel compounds with potential anticancer and antibacterial properties against drug-resistant strains [80,81]. In this study, we isolated and characterized two endophytic *Streptomyces* strains from the soil in Manang, Nepal, to evaluate their potential to produce bioactive metabolites within this unique ecosystem. A molecular analysis, including genomic DNA extraction and 16S rRNA gene amplification, identified the strains as *Streptomyces* isolates PY108 and PY109 at the genus level. These morphological and genomic investigations revealed that *Streptomyces* sp. PY108 is closely related to *Streptomyces hundungensis* PSB170 (99.28% similarity), while PY109 is most similar to *Streptomyces* sp. Ed-065 (99.36% similarity).

The EA extracts from fermented *Streptomyces* sp. PY108 and PY109 demonstrated antifungal, cytotoxic, and antibacterial properties. Both extracts were effective in inhibiting the growth of *S. aureus*, *S. sonnei*, and *K. pneumoniae*, although their effectiveness against *E. coli* was comparatively lower. This variation may be attributed to the differences in the susceptibility of these species to the specific compounds present in the extracts, which could affect membrane permeability and ion leakage [82]. Notably, *Streptomyces* sp. PY108 exhibited superior antibacterial efficacy compared with *Streptomyces* sp. PY109, as evidenced by lower minimum inhibitory concentration (MIC) and minimum bactericidal concentration (MBC) values. The varying responses of bacteria to antibacterial agents can be influenced by their unique characteristics and mechanisms [83]. These findings suggest that these extracts possess significant antibacterial potential and highlight the value of exploring microbial sources like *Streptomyces* in discovering novel antibiotics. Furthermore, the EA extracts showed moderate antifungal activity against *Saccharomyces cerevisiae*, with *Streptomyces* sp. PY108 demonstrating slightly higher activity compared with *Streptomyces* sp. PY109. In contrast, both extracts exhibited lower inhibitory activity against *A. niger*, indicating a more specific action against certain fungal strains. Both extracts also revealed cytotoxic effects against cervical (HeLa) and breast cancer (MCF-7) cells.

To identify the potential metabolites responsible for the observed antibacterial, antifungal, and anticancer activities, the bioactive organic extracts of *Streptomyces* sp. PY108 and PY109 were analyzed using LC-HRMS/MS and GC-MS techniques, followed by GNPS-based molecular networking. The LC-HRMS/MS analyses detected various specialized metabolites in the EA extracts of both *Streptomyces* strains. A total of 27 metabolites were annotated from these extracts. Additionally, GNPS-based metabolic profiling led to the dereplication of 19 known compounds. The overlap between the metabolites identified through the GNPS-based analysis and manual interpretation underscores the reliability of the dereplication process across different methods. Both the PY108 and PY109 strains shared several metabolites in their EA extracts (Table 1 and Table 2), suggesting that they may possess common BGCs due to their comparable ecological niches or evolutionary backgrounds (horizontal gene transfer).

The dereplicated metabolites included diketopiperazine derivatives (e.g., maculosin, cyclo-(D-Pro-L-Val), cyclo-(D-Phe-L-Pro)), aureolic acid derivatives (e.g., chromomycin A3, chromomycin A2, chromomycin A2-1, chromomycin A3-1), alkyl diethanolamine (e.g., *N*-lauryl diethanolamine), *N*-acyl amines (e.g., *N*-acetyltyramine), and lipopeptides (e.g., surfactin B, surfactin C). Many of the diketopiperazines identified in both samples, such as cyclo-(l-Trp-l-Phe) [84], cyclo-(d-Phe-l-Pro) [85], maculosin [46], brevianamide F [86], and cyclo-(l-leucyl-l-leucyl) [43], are known for their antibacterial potential. Additionally, compounds such as maculosin [33], cyclo-(d-Pro-l-Val) [87], cyclo-(2-hydroxy-Pro-R-Leu) [35], gancidin W [88], cyclo-(d-Phe-l-Pro) [38,89], and brevianamide F [90,91] have shown antifungal or anticancer activities. Thus, the diketopiperazine derivatives in the EA extracts of both *Streptomyces* species may contribute significantly to the observed antibacterial, antifungal, and anticancer properties in this study.

Interestingly, we identified the anthraquinone antibiotic glycoside, chromomycin A3 in *Streptomyces* sp. PY108. This antibiotic was also detected in the EA extract of *Streptomyces* sp. PY108 during the GNPS library search. Chromomycin A3 has been previously isolated from the EA extract of *Streptomyces* sp. MBTI36, which is known for its potent antibacterial activity against methicillin-resistant *S. aureus* (MRSA) [92]. Additionally, chromomycin A3 derived from marine sediment-associated *Streptomyces* sp. KMM 9048 has demonstrated strong antibacterial activity against Gram-positive bacteria [50]. Isolates of chromomycin A3 from *S. griseus* and *S. cavourensis* have shown antibacterial activity against Gram-positive bacteria, such as *Bacillus subtilis*, *S. aureus*, and *Enterococcus hirae*. It has exhibited considerable antibacterial activity against *S. aureus*, *E. faecium*, and *E. faecalis*, with lower MIC values compared with important antibiotics like vancomycin and linezolid [92]. Thus, the presence of chromomycin A3 exclusively in *Streptomyces* sp. PY108 might account for its higher antibacterial activity compared with *Streptomyces* sp. PY109. In addition to its antimicrobial properties, chromomycin A3 is known for its cytotoxic effects. It binds to DNA and inhibits transcription and replication, demonstrating significant cytotoxicity [93]. For instance, chromomycin A3 significantly reduced colony formation by 82% in RPMI-7951 and by 72% in SK-Mel-28 cancer cells at a concentration of 5 nM [50]. As a member of the aureolic acid family, chromomycin A3 forms dimeric complexes with Mg²^+^ at the CG region of DNA through a non-chelating process, blocking DNA replication and transcription, which contributes to its anticancer activity [92]. Chromomycin A2, another aureolic acid derivative, exhibits similar anticancer mechanisms but with a more pronounced effect due to an additional acetyl group that enhances its interaction with DNA in cancer cells [94].

Actinomycin C2 (also known as actinomycin D), which was detected only through the GC-MS analysis and not LC-MS, is renowned for its potent antitumor activity and is clinically used in cancer therapy due to its ability to inhibit nucleic acid synthesis [95]. Although the detection of actinomycin C2 was hindered in the LC-MS analysis—likely due to ion suppression during ionization—it showed significant cytotoxicity against human cancer cell lines (HeLa, PC-3, THP-1, and Caco-2) [96]. Cyclo-(2-hydroxy-Pro-R-Leu), isolated from a marine-derived *Streptomyces* sp., has demonstrated anticancer activity against HL-60 cell lines [35]. Additionally, *N*-phenethylacetamide from *Aquimarina* sp. MC085 has been reported to inhibit the TGF-β/Smad pathway, reducing metastasis in A549 human lung cancer cells [97].

Surfactin and its derivatives are cyclic lipopeptides derived from the *Bacillus* genus, known for their antimicrobial and anticancer properties [98,99]. The antimicrobial efficacy of surfactin stems from its ability to permeate plasma membranes and its remarkable surface, interface, and membrane-active properties [100]. Additionally, surfactin’s anticancer effects are attributed to the hydrophobic nature of its fatty acid chain, which interacts with the acyl chains of phospholipids in cancer cell membranes [101]. Therefore, it can be hypothesized that other surfactin derivatives, such as surfactin B and surfactin C, detected in this study, might exhibit similar antimicrobial and anticancer properties due to their structural similarities to surfactin.

Moreover, we used the GC-MS technique to identify non-polar and volatile metabolites in the organic extracts of both *Streptomyces* species. Thirty-four volatile compounds from diverse classes, such as polypeptide antibiotics, steroids, acids, cyclic peptides, and other nitrogen-containing compounds, were detected. γ-Sitosterol, previously identified for its antibacterial and antifungal properties [102], was also found in this study. Notably, this compound is known to induce apoptosis in cancer cells, demonstrating anticancer activity [95]. The presence of γ-sitosterol and actinomycin D exclusively in *Streptomyces* sp. PY108 could explain the enhanced antibacterial and antifungal efficacy observed in this strain compared with *Streptomyces* sp. PY109. The literature reports that actinomycin D is an effective fungal growth inhibitor [103], suggesting that these metabolites might have contributed to the better antifungal outcomes observed in *Streptomyces* sp. PY108.

The metabolites detected in *Streptomyces* sp. PY108 and PY109 are known to possess antibacterial, antifungal, and anticancer properties according to the existing literature, and this study has similarly shown potential correlations with these previously observed bioactivities. Notably, these 27 metabolites had not been reported previously in extracts from the *Streptomyces hundungensis* PSB170 strain and *Streptomyces* sp. Ed-065 strain. The identification of these metabolites enriches the emerging database of natural products and contributes to our understanding of their biological activities and therapeutic applications. The presence of these compounds in both PY108 and PY109 suggests that these strains may share similar biosynthetic pathways, likely due to their common ecological niches or evolutionary histories. This finding implies that the specialized metabolites present in the organic extracts of these *Streptomyces* species may offer a novel source in developing valuable medications. Therefore, further research involving the isolation, characterization, and assessment of these annotated metabolites is warranted to explore their potential as therapeutic agents. This research also opens the door to discovering additional therapeutics through new fermentation methods, beyond those reported in this study.

## 5. Conclusions

In our study, two endophytic *Streptomyces* strains, PY108 and PY109, were isolated from soil samples in the Manang district of Nepal. Microscopic observation revealed characteristic *Streptomyces* features, such as spore formation and filamentous growth. Genetic analysis showed that the strains PY108 and PY109 were closely related to *Streptomyces hundungensis* PSB170 and *Streptomyces* sp. Ed-065, respectively, based on their 16S rRNA gene sequences. EA extracts from both strains exhibited significant antibacterial activity, as evidenced by substantial zones of inhibition and low minimum inhibitory concentration (MIC) and minimum bactericidal concentration (MBC) values, comparable with those of the controls. Additionally, both extracts showed notable antifungal activity against *S. cerevisiae* and *A. niger*, as well as significant anticancer potential against cervical cancer (HeLa) and breast cancer (MCF-7) cells. Analytical techniques, including LC-MS/MS, GC-MS, and GNPS-based molecular networking, were employed to identify the metabolites responsible for these biological activities. The analyses revealed 27 specialized metabolites in the LC-MS/MS analysis, 34 volatile metabolites in the GC-MS analysis, and 24 metabolites in the molecular networking analysis. The presence of certain diketopiperazine derivatives, aureolic acid derivatives, lipopeptide derivatives, actinomycin D, and γ-sitosterol in the EA extract of *Streptomyces* sp. PY108 was identified as a key factor contributing to its higher biological activity compared with *Streptomyces* sp. PY109. To the best of our knowledge, while some of the identified diketopiperazines and aureolic acid derivatives have been previously evaluated for biological activity, many remain unstudied. This highlights the need for further research to isolate and evaluate the biological potential of these compounds in the development of new therapeutic agents.

## Figures and Tables

**Figure 5:**
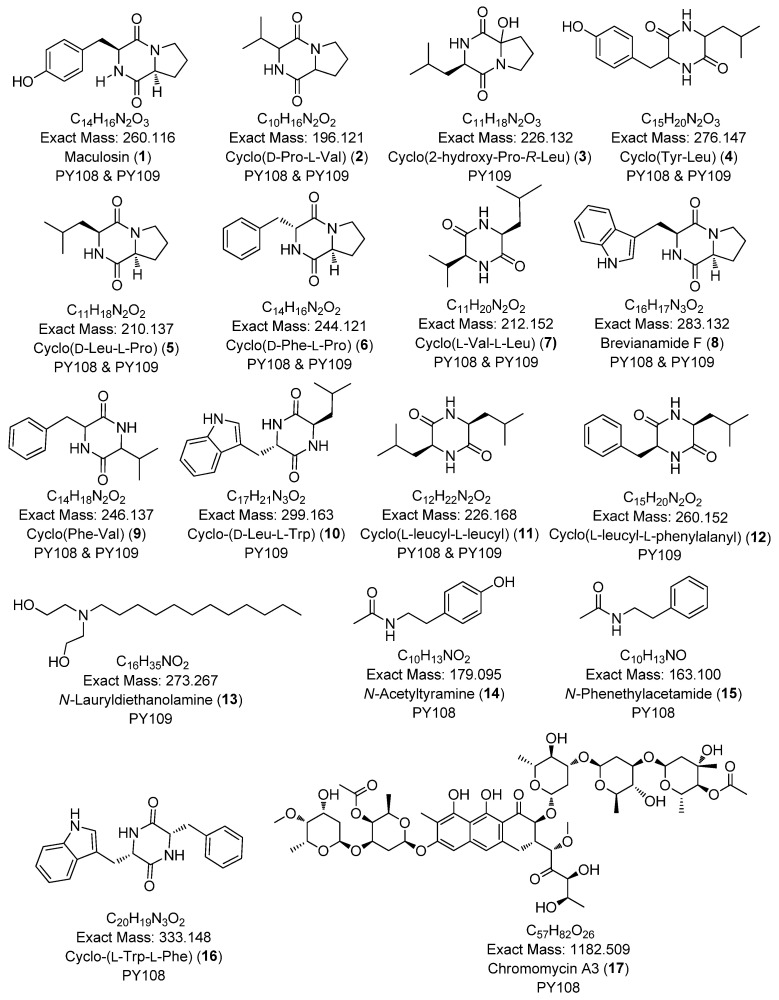

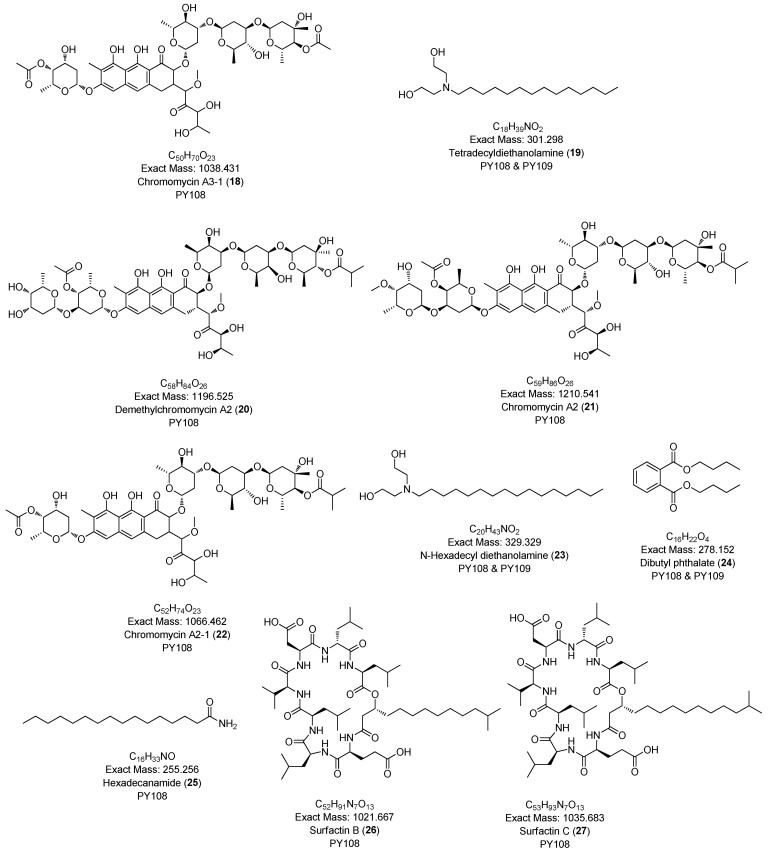
Chemical structures of annotated compounds in *Streptomyces* sp. PY108 and PY108 using an LC-MS/MS analysis.

**Table 1:** The list of annotated compounds in *Streptomyces* sp. PY108 and PY109 using an LC-HRMS/MS analysis.

C.N	Annotated Compound	Exact Mass *m*/*z*	Observed Mass *m*/*z*	Detected Ion	Molecular Formula	RDBE	Absolute Error (ppm)	Retention Time (min)	Sources	CSI:FingerID Score (%)	References
1	Maculosin	260.115	261.124	[M+H]^+^	C_14_H_16_N_2_O_3_	8.0	3.25	3.49	PY109 PY108 (3.50)	92.41	[33]
2	Cyclo-(d-Pro-l-Val)	196.121	197.129	[M+H]^+^	C_10_H_16_N_2_O_2_	4.0	4.06	3.76	PY109 PY108 (3.38)	95.52	[34]
3	Cyclo(2-hydroxy-Pro-R-Leu)	226.131	227.139	[M+H]^+^	C_11_H_18_N_2_O_3_	4.0	2.04	3.98	PY109	-	[35]
4	Cyclo(Tyr-Leu)	276.147	277.155	[M+H]^+^	C_15_H_20_N_2_O_3_	7.0	2.98	4.25	PY109 PY108 (4.21)	78.98	[36]
5	GancidinW Or, Cyclo-(d-Leu- l-Pro)	210.136	211.145	[M+H]^+^	C_11_H_18_N_2_O_2_	4.0	3.90	4.43	PY109 PY108 (4.41)	98.69	[37]
6	Cyclo-(d-Phe-l-Pro)	244.120	245.129	[M+H]^+^	C_14_H_16_N_2_O_2_	8.0	2.05	4.93	PY109 PY108 (4.98)	100	[38]
7	Cyclo-(l-Val-l-Leu)	212.152	213.161	[M+H]^+^	C_11_H_20_N_2_O_2_	3.0	4.35	5.52	PY109 PY108 (5.50)	75.92	[39]
8	Brevianamide F	283.131	284.139	[M+H]^+^	C_16_H_17_N_3_O_2_	10.0	0.47	5.56	PY109 PY108 (5.57)	100	[40]
9	Cyclo-(l-Valyl-Phenylalanyl) Or, Cyclo-(Phe-Val)	246.136	247.144	[M+H]^+^	C_14_H_18_N_2_O_2_	7.0	1.46	6.02	PY109 PY108 (5.98)	88.89	[41]
10	Cyclo-(d-Leu-l-Trp)	299.163	300.170	[M+H]^+^	C_17_H_21_N_3_O_2_	9.0	0.58	6.43	PY109	86.94	[42]
11	Cyclo-(l-Leucyl-l-Leucyl)	226.167	227.176	[M+H]^+^	C_12_H_22_N_2_O_2_	3.0	2.06	6.47	PY109 PY108 (6.42)	77.84	[43,44]
12	Cyclo-(l-Leucyl-l-Phenylalanyl)	260.152	261.160	[M+H]^+^	C_15_H_20_N_2_O_2_	7.0	0.83	6.84	PY109	94.47	[44]
13	*N*-Lauryldiethanolamine	273.26	274.274	[M+H]^+^	C_16_H_35_NO_2_	0.0	1.63	10.28	PY109	99.17	[45]
14	*N*-Acetyltyramine	179.094	180.102	[M+H]^+^	C_10_H_13_NO_2_	5.0	2.23	3.86	PY108	69.47	[46]
15	*N*-Phenethylacetamide	163.099	164.107	[M+H]^+^	C_10_H_13_NO	5.0	2.72	5.93	PY108	86.52	[47]
16	Cyclo-(l-Trp-l-Phe)	333.147	334.155	[M+H]^+^	C_20_H_19_N_3_O_2_	13.0	0.42	7.20	PY108	96.26	[48]
17	Chromomycin A3 (Aburamycin B)	1182.529	1205.497	[M+Na]^+^	C_57_H_82_O_26_	17.0	1.40	11.46	PY108	-	[49]
18	Chromomycin A3-1	1038.431	1061.418	[M+Na]+	C_50_H_70_O_23_	16.0	1.65	11.55	PY108	-	[50]
19	Tetradecyldiethanolamine	301.298	302.306	[M+H]^+^	C_18_H_39_NO_2_	0.0	1.26	12.00	PY108 PY109 (12.00)	95.83	[51,52]
20	Demethlychromomycin A2	1196.525	1219.513	[M+Na]^+^	C_58_H_84_O_26_	17.0	1.37	12.18	PY108	-	[53]
21	Chromomycin A2 (Aburamycin A)	1210.541	1233.526	[M+Na]^+^	C_59_H_85_O_26_	17.0	2.99	12.86	PY108	-	[54]
22	Chromomycin A2-1	1066.462	1067.470	[M+H]^+^	C_52_H_74_O_23_	16.0	0.76	12.86	PY108	-	[50]
23	*N*-Hexadecyl diethanolamine	329.329	330.337	[M+H]^+^	C_20_H_43_NO_2_	0.0	0.30	13.59	PY108 PY109 (13.58)	98.33	[52]
24	Dibutyl Phthalate	278.152	279.160	[M+H]^+^	C_16_H_22_O_4_	6.0	2.73	15.81	PY108 PY109 (15.77)	93.68	[55]
25	Hexadecanamide	255.256	256.263	[M+H]^+^	C_16_H_33_NO	1.0	2.51	18.44	PY108	95.69	[56]
26	Surfactin B	1021.667	1022.673	[M+H]^+^	C_52_H_91_N_7_O_13_	11.0	1.24	20.25	PY108	-	[57]
27	Surfactin C	1035.683	1036.688	[M+H]^+^	C_53_H_93_N_7_O_13_	11.0	2.77	20.79	PY108	-	[57]

Note: C.N (compound number), RDBE (ring double bond equivalents). To facilitate easier tracking, the isolate name has been provided with a retention time.

**Table 2:** The list of annotated compounds using GNPS in *Streptomyces* species PY108 and PY109.

C.N.	Annotated Compound	Accurate Mass (Da)	Precursor Ion	Adduct Type	MS^2^ Fragmentation Pattern	Molecular Formula	Retention Time (min)	Sources	Error (ppm)	Reference
Diketopiperazines derivatives										
1	Cyclo(L-Leu-L-4-hydroxy-Pro)	226.132	227.139	[M+H]^+^	136.113, 86.060	C_11_H_18_N_2_O_3_	3.78	PY108 PY109	0.0	[58]
2	Cyclo(Phe-Leu)	260.152	261.159	[M+H]^+^	120.080	C_15_H_20_N_2_O_2_	3.46	PY108 PY109	0.0	[59]
3	Indoleacetic acid	175.063	176.071	[M+H]^+^	130.065, 103.054, 77.038	C_10_H_9_NO_2_	6.33	PY109	0.0	[60]
4	Cyclo(L-Pro-L-Tyr)	260.116	261.123	[M+H]^+^	261.123, 107.049	C_14_H_16_N_2_O_3_	6.76	PY108 PY109	0.0	[61]
5	N6-Isopentenyladenosine	335.1594	336.167	[M+H]^+^	204.124, 148.061, 136.062	C_15_H_21_N_5_O_4_	5.11	PY109	2.9	[62]
6	Isorugulosuvine	333.147	334.155	[M+H]^+^	130.064	C_20_H_19_N_3_O_2_	7.19	PY108 PY109	3.0	[63]
7	10β-Hydroxy-3-isobutyl-6,10β,11,11a-tetrahydro-2H-pyrazino[1′,2′:1,5]pyrrolo[2,3-b]indole-1,4(3H,5aH)-dione	315.158	316.166	[M+H]^+^	298.155, 130.064	C_17_H_21_N_3_O_3_	6.20	PY108 PY109	3.0	[64]
8	Brevianamide F	283.132	284.139	[M+H]^+^	130.064	C_16_H_17_N_3_O_2_	5.36	PY108 PY109	3.5	[65]
9	Dibutyl phthalate	278.152	279.159	[M+H]^+^	149.023, 121.028, 65.038	C_16_H_22_O_4_	15.76	PY108 PY109	3.6	[66]
10	Cyclo(Phe-4-hydroxy-Pro)	260.116	261.124	[M+H]^+^	170.077, 120.080, 86.060, 68.049	C_14_H_16_N_2_O_3_	4.23	PY108 PY109	3.8	[67]
11	Cyclo(L-Phe-L-Pro)	244.121	245.130	[M+H]^+^	154.072, 120.080, 98.059, 70.065	C_14_H_16_N_2_O_2_	5.22	PY108 PY109	8.1	[68]
12	*N*-[2-(1H-Indol-3-yl)ethyl]acetamide	202.111	203.118	[M+H]^+^	144.081	C_12_H_14_N_2_O	6.32	PY108	4.8	[69]
13	3-(2-Hydroxyethyl)indole	161.084	144.081	[M+H-H_2_O]^+^	155.060, 143.072, 115.054, 91.054	C_10_H_11_NO	6.33	PY108	6.8	[70]
14	Pyridoxine	169.074	170.081	[M+H]^+^	134.060, 106.065, 77.038, 65.038	C_8_H_11_NO_3_	1.09	PY108 PY109	0.0	[71]
15	Indole-3-carbinol	147.068	130.066	[M+H-H_2_O]^+^	103.056, 95.050, 77.038, 51.045	C_9_H_9_NO	5.88	PY108 PY109	7.62	[72]
Aureolic acid derivatives										
16	Chromomycin A3	1182.509	1205.5	[M+Na]^+^	889.346, 469.204, 357.151	C_57_H_82_O_26_	11.49	PY108	4.15	[73]
Lipopeptides and *N*-Acylethanolamines										
17	Surfactin C	1035.683	1036.690	[M+H]^+^	685.449, 596.426, 554.354, 441.270	C_53_H_93_N_7_O_13_	20.20	PY108	8.6	[74]
18	*N*-Lauryldiethanolamine	273.266	274.274	[M+H]^+^	88.075, 70.065, 57.069	C_16_H_35_NO_2_	10.27	PY108 PY109	0.0	[75]
19	*N*-Tetradecyldiethanolamine	301.298	302.305	[M+H]^+^	88.075, 70.065, 57.069	C_18_H_39_NO_2_	10.01	PY108 PY109	0.0	[52]
20	*N*-Lauroylethanolamine	243.219	244.227	[M+H]^+^	81.069, 67.054, 62.059, 57.069	C_14_H_29_NO_2_	12.95	PY109	0.0	[76]
21	13-Docosenamide	337.3345	338.342	[M+H]^+^	149.132, 121.101, 97.101, 69.070	C_22_H_43_NO	11.22	PY108 PY109	2.9	[77]
22	Palmitoyl ethanolamide	299.2824	300.289	[M+H]^+^	300.288, 283.264, 62.060	C_18_H_37_NO_2_	17.94	PY108 PY109	3.3	[78]
23	Leu-C14:0	341.293	342.299	[M+H]^+^	132.101, 86.096	C_20_H_39_NO_3_	17.48	PY108 PY109	2.85	[79]
24	Leu-C15:0	355.309	356.316	[M+H]^+^	132.101, 86.096	C_20_H_39_NO_3_	19.02	PY108 PY109	0.0	[79]

Note: C.N (Compound Number).

**Table 3:** List of volatile compounds identified in the EA extracts of *Streptomyces* sp. PY108 and PY109 using GC-MS analysis.

C.N	Name of Volatile Compounds	Retention Time (min)	Molecular Formula	Sources
1	2-Propenoic acid	7.92	C_17_H_32_O_2_	PY108
2	Benz[e]azulene-3,8-dione	10.00	C_19_H_24_O_6_	PY108
3	Pyrrolo[1,2-a]pyrazine-1,4-dione, hexahydro-3-(2-methyl propyl)-	11.07	C_11_H_18_N_2_O_2_	PY108
4	Actinomycin C2 Or Actinomycin D	11.69	C_63_H_88_N_12_O_16_	PY108
5	1,9-Dioxacyclohexadeca-4,13-diene-2-10-dione, 7,8,15,16-tetramethyl-	13.39	C_18_H_28_O_4_	PY108
6	1-Phenyl-3,6-diazahomoadamantane	17.63	C_15_H_20_N_2_	PY108
7	Ergotaman-3′,6′,18-trione,	18.61	C_33_H_35_N_5_O_5_	PY108
8	Ethyl iso-allocholate	21.90	C_26_H_44_O_5_	PY108
9	γ-Sitosterol	25.25	C_29_H_50_O	PY108
10	1H-Cyclopropa[3,4]benz[1,2-e]azulene-4a,5,7b,9,9a(1aH)-pentol	26.82	C_28_H_38_O_10_	PY108
11	3′H-Cycloprop(1,2)-5-cholest-1-en-3-one	27.31	C_26_H_44_O_5_	PY108
12	3′H-Cycloprop(1,2)-5α-cholest-1-en-3-one,1′,1′-dicarboethoxy-1β,2β-dihydro	27.49	C_34_H_54_O_5_	PY108
13	7aH-Cyclopenta[a]cyclopropa[f]cycloundecene-2,4,7,7a,10,11-hexol	29.69	C_30_H_44_O_11_	PY108
14	Carda-16,20(22)-dienolide	31.09	C_30_H_36_O_11_	PY108
15	D-Homo-24-nor-17-oxachola-20,22-dien16-one,	32.39	C_32_H_42_O_10_	PY108
16	Pyrrolizin-1-one, 7-propyl-	14.17	C_10_H_17_NO	PY109
17	2,4-Di-tert-butylphenol	15.29	C_14_H_22_O	PY109
18	Cetene	17.12	C_16_H_32_	PY109
19	Benzophenone	17.91	C_13_H_10_O	PY109
20	8-Pentadecanone	18.91	C_15_H_30_O	PY109
21	1,4-diazabicyclo[4.3.0]nonan-2,5-dione, 3-methyl	19.61	C_8_H_12_N_2_O_2_	PY109
22	Uric Acid	20.12	C_5_H_4_N_4_O_3_	PY109
23	Pyrrolo[1,2-a]pyrazine-1,4-dione, hexahydro-	20.52	C_7_H_10_N_2_O_2_	PY109
24	Imidazole-4-carboxylic acid, 2-fluoro-1-methoxymethyl-, ethyl ester	20.89	C_8_H_11_N_2_	PY109
25	3,4-Methylenedioxyamphetamine	21.06	C_10_H_13_NO_2_	PY109
26	*E*-15-Heptadecenal	21.46	C_17_H_32_O	PY109
27	Cyclo(L-Pro-L-Val)	21.94	C_10_H_16_N_2_O_2_	PY109
28	7-Ethylpentadecane-4,6-dione	22.74	C_17_H_32_O_2_	PY109
29	10-Nonadecanone	23.35	C_19_H_38_O	PY109
30	Pyrrolo[1,2-a]pyrazine-1,4-dione, hexahydro-3-(2-methylpropyl)-	24.33	C_11_H_18_N_2_O_2_	PY109
31	Dibutyl Phthalate	25.83	C_16_H_22_O_4_	PY109
32	1-Henicosyl formate	26.66	C_22_H_44_O_2_	PY109
33	*dl*-Alanyl-*dl*-phenylalanine	30.09	C_12_H_16_N_2_O_3_	PY109
34	2,5-Piperazinedione, 3,6-bis(2-methylpropyl)-	30.60	C_12_H_22_N_2_O_2_	PY109

Note: C.N (compound number).

## Data Availability

The original contributions presented in the study are included in the article/Supplementary Material, further inquiries can be directed to the corresponding author.

## Supplementary Materials

The following supporting information can be downloaded at: https://www.mdpi.com/article/10.3390/biomedicines12102192/s1, Figure S1. Isolation of actinomycetes from soil samples; Figure S2. Isolation of genomic DNA; Figure S3. PCR amplification of 16S rRNA; Figure S4. Neighbor-joining phylogenetic tree of 16S rRNA; Figure S5. Antibacterial assay of *Streptomyces* sp. PY108 and PY109; Table S1. Zone of inhibition of *Streptomyces* sp. extracts; Figure S6. MIC of EA extracts of *Streptomyces* species; Figure S7. MBC of EA extracts of *Streptomyces* species; Table S2. MIC and MBC values of *Streptomyces* sp.; Table S3. Antifungal activity of EA extracts of *Streptomyces* sp.; Figure S8. Antifungal activity against *Saccharomyces cerevisiae*; Figure S9. Antifungal activity against *Aspergillus niger*; Figures S10–S36. BPC and LC-MS/MS profiles of various metabolites; Figures S37–S72. GC-MS chromatogram obtained in EA extracts.
