# Supplementary material for: Antibacterial, Antifungal, and Cytotoxic Effects of Endophytic Streptomyces Species Isolated from the Himalayan Regions of Nepal and Their Metabolite Study
Source: Biomedicines. 2024 Sep 26;12(10):2192. doi: 10.3390/biomedicines12102192 (PMC11505041; doi:10.3390/biomedicines12102192)
Supplement: Supplementary file 1 [file biomedicines-12-02192-s001.zip › biomedicines-3209673-supplementary.pdf]

Antibacterial, Antifungal, and Cytotoxic Effects of Endophytic *Streptomyces* Species Isolated from the Himalayan Regions of Nepal and Their Metabolite Study

Ram Prabodh Yadav, Chen Huo, Rabin Budhathoki, Padamlal Budthapa, Bibek Raj Bhattarai, Monika Rana, Ki Hyun Kim and Niranjan Parajuli

Samples	A. Isolation	B. Subculture	C. Gram-staining
PY108	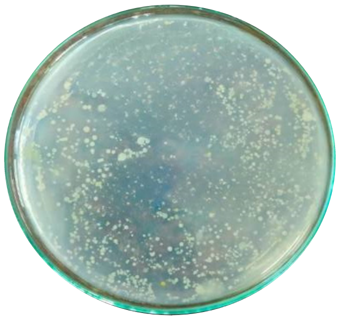	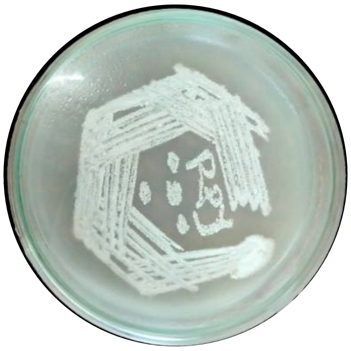	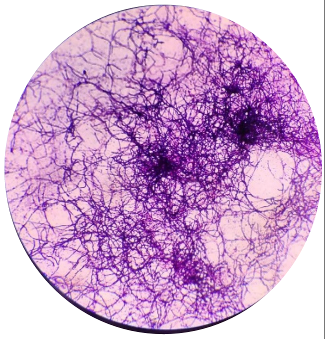
PY109	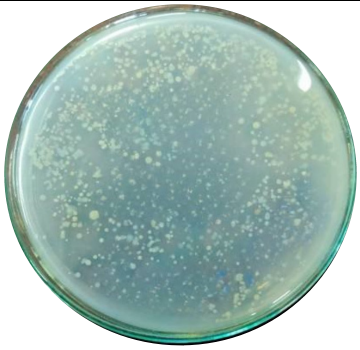	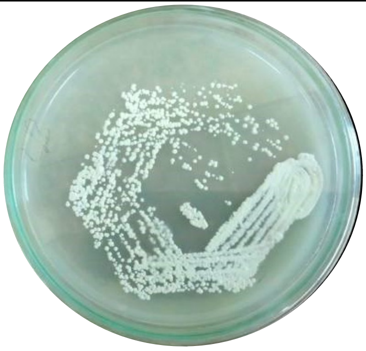	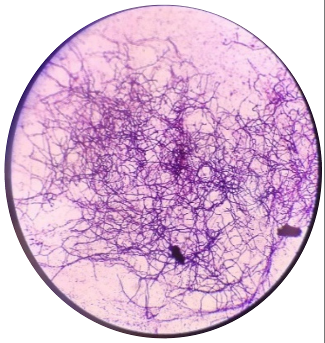

Figure S1. (A) Isolation of actinomycetes from soil samples by using ISP4 media after a week of incubation, which represents the morphology of colonies (white or greyish white) (B) Subculture of those isolates by picking up the immersed colony for pure culture on the same media, (C) Respective microscopic feature of mycelia at 100X oil immersion of four representative genera of actinomycetes isolates (PY108, and PY109)

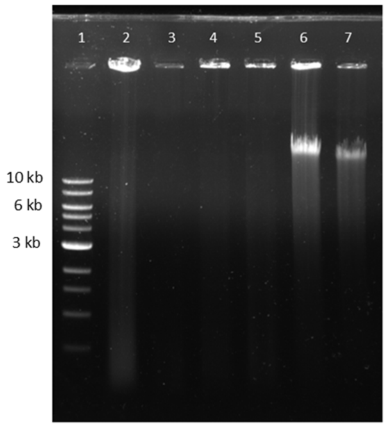

Figure S2. Isolation of genomic DNA from *Streptomyces* species; lane 1 (1 kb DNA ladder), lane 2-5 (Diluted genomic DNA not detected in the gel), lane 6 (genomic DNA of *Streptomyces* sp. PY108), and lane 7 (genomic DNA of *Streptomyces* sp. PY109).

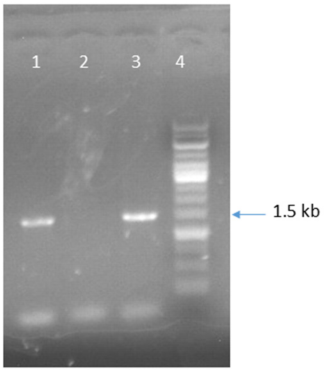

Figure S3. PCR amplification of 16S rRNA from *Streptomyces* species by using universal primers.; lane 1 (PCR product of *Streptomyces* sp. PY108), lane 2 (PCR product not detected), lane 3 (PCR product of *Streptomyces* sp. PY109), and lane 4 (1 kb DNA ladder).

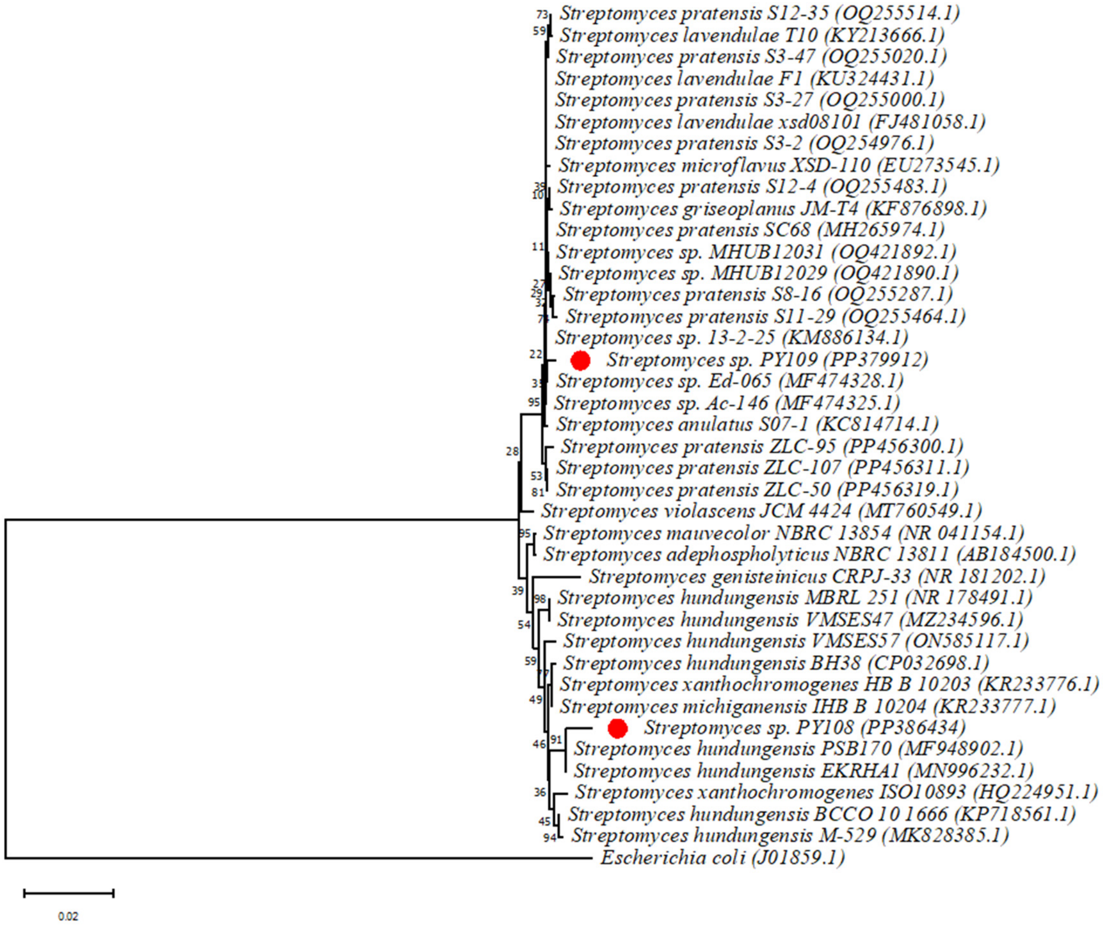

Figure S4. Neighbor-joining phylogenetic tree of 16S rRNA of *Streptomyces* strains PY108 and PY109 showing their evolutionary relationship to closet identified taxa. The bar 0.02 indicates substitutions per nucleotide position, as determined using 1000 bootstrap replications. Accession numbers are shown in parentheses. *E. coli* acts as out-group strains, with PY108 and PY109 being closely related to their identified taxa.

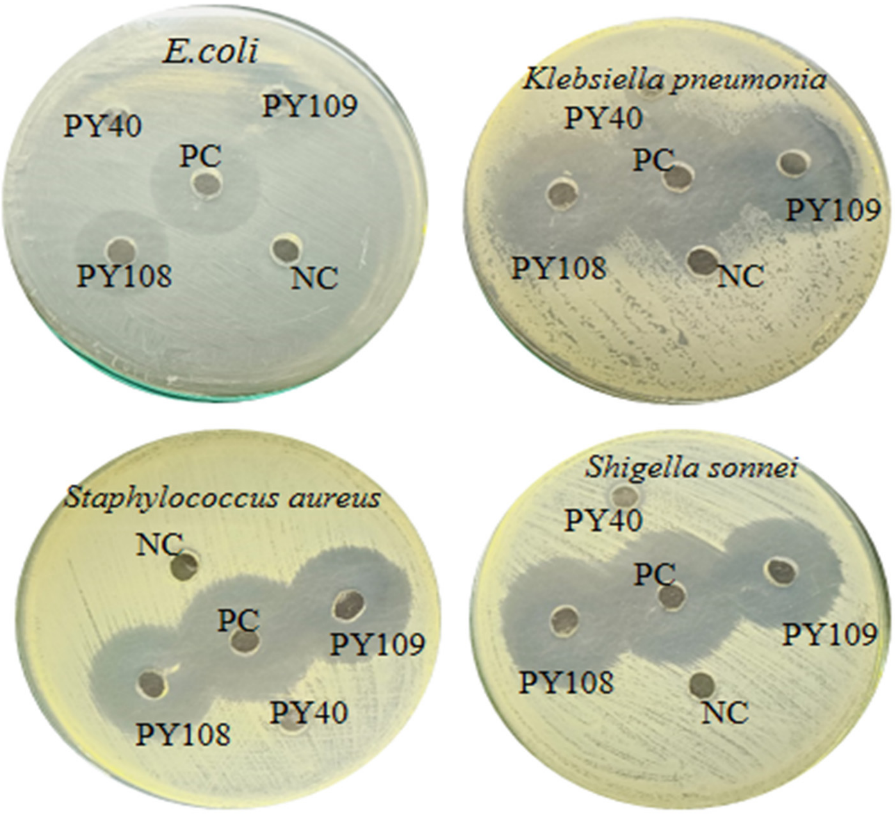

Figure S5. Antibacterial assay of *Streptomyces* sp. PY108 and PY109 against *E. coli*, *Shigella sonnei*, *Staphylococcus aureus,* and *Klebsiella pneumonia*. All results for PY40 presented in this figure are not related to the data discussed in this paper.

Table S1. Zone of inhibition of ethyl acetate (EA) extracts of *Streptomyces* sp. PY108 and *Streptomyces* sp**.** PY109 against tested pathogenic bacteria.

**Samples**	**Zone of Inhibition (in mm)**			
	***Escherichia coli* ATCC 25922**	***Shigella sonnei* ATCC 25931**	***Staphylococcus aureus* ATCC 43300**	***Klebsiella pneumoniae* ATCC 700603**
Streptomyces sp. PY108	15	28	26	30
Streptomyces sp. PY109	17	26	27	29
Neomycin	22	29	28	32

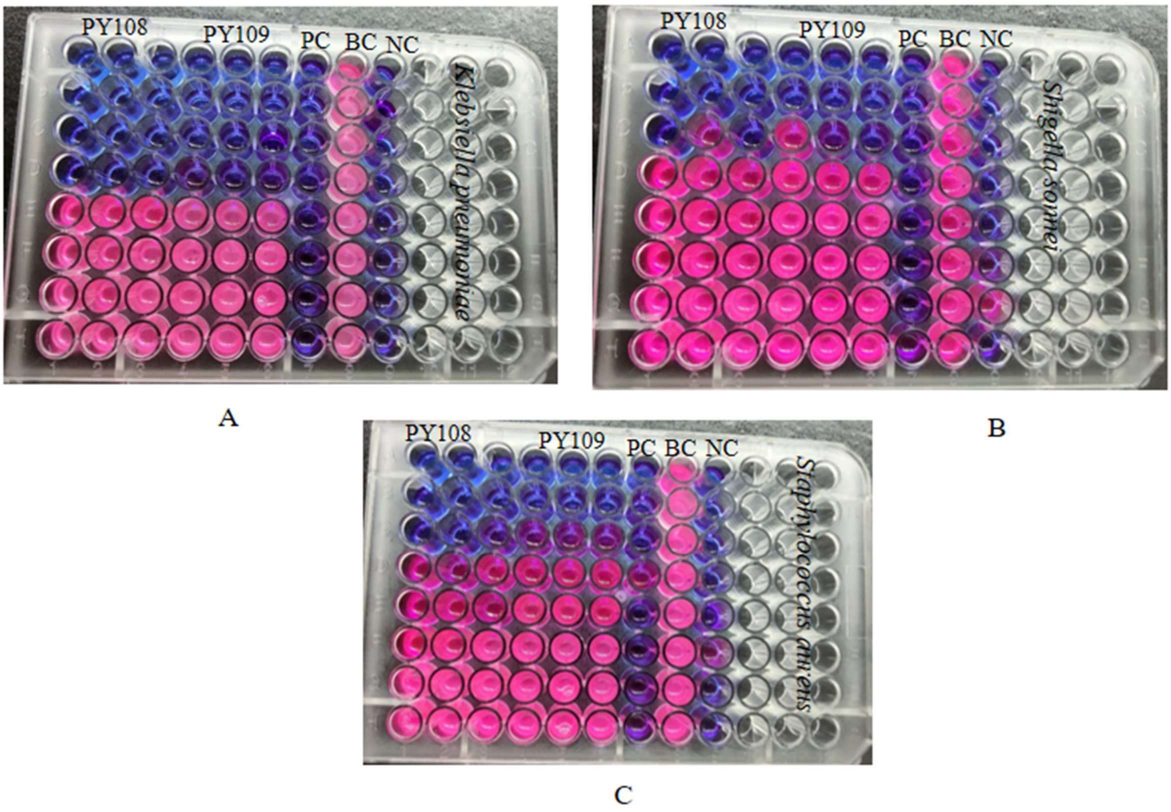

Figure S6. MIC of EA extracts of *Streptomyces* species PY108 and PY109.

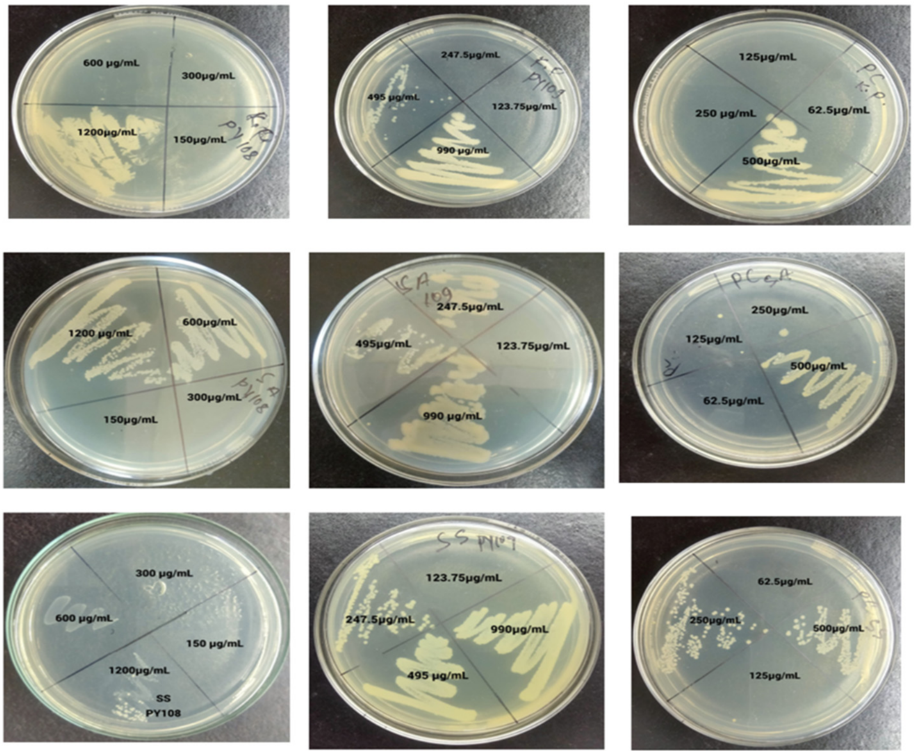

Figure S7. MBC of EA extracts of *Streptomyces* species PY108 and PY109.

Table S2. MIC and MBC values of *Streptomyces* sp. PY108 and PY109 against tested bacterial strains.

**Tested bacterial strains**	***Streptomyces* sp. PY108**		***Streptomyces* sp. PY109**		**Positive Control**	
	**MIC (µg/mL)**	**MBC (µg/mL)**	**MIC (µg/mL)**	**MBC (µg/mL)**	**MIC (µg/mL)**	**MBC (µg/mL)**
Shigella sonnei ATCC 25931	600	600	990	990	15.625	250
Staphylococcus aureus ATCC 43300	600	600	990	1200	7.8125	125
Klebsiella pneumoniae ATCC 700603	300	300	247.5	495	7.8125	125

Table S3. Antifungal activity of EA extracts of *Streptomyces* sp. PY108 and PY109.

Fungal mycelium	Zone of Inhibition (in mm)		
	***Streptomyces* sp. PY108**	***Streptomyces* sp. PY109**	**Positive control** (Cycloheximide)
Saccharomyces cerevisiae	24	19	28
Aspergillus niger	12	11	21

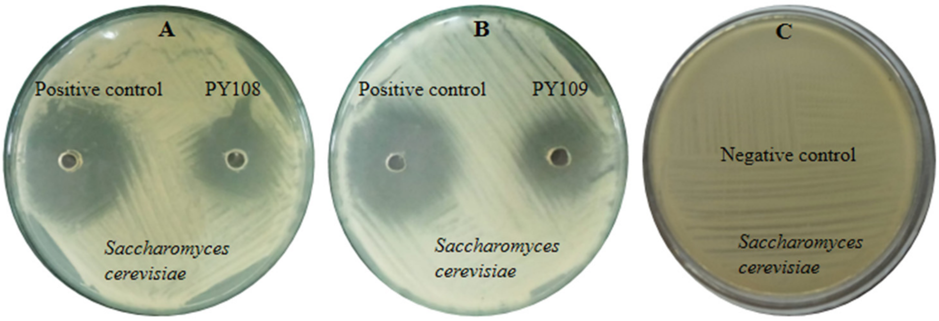

Figure S8. Antifungal activity against *Saccharomyces cerevisiae* (**A**) *Saccharomyces cerevisiae* on EA extract of *Streptomyces* sp. PY108, (**B**) *Saccharomyces cerevisiae* on EA extract of *Streptomyces* sp. PY109, and (**C**) *Saccharomyces cerevisiae* itself (negative control).

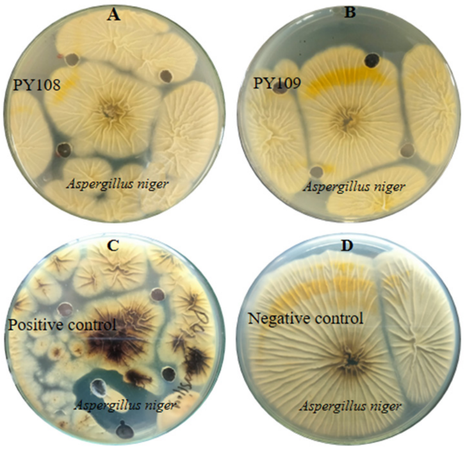

Figure S9. Antifungal activity against *Aspergillus niger* (**A**) *Aspergillus niger* on EA extract of *Streptomyces* sp. PY108, (**B**) *Aspergillus niger* on EA extract of *Streptomyces* sp. PY109, (**C**) *Aspergillus niger* on Cycloheximide (positive control), and (**D**) *Aspergillus niger* itself (negative control)

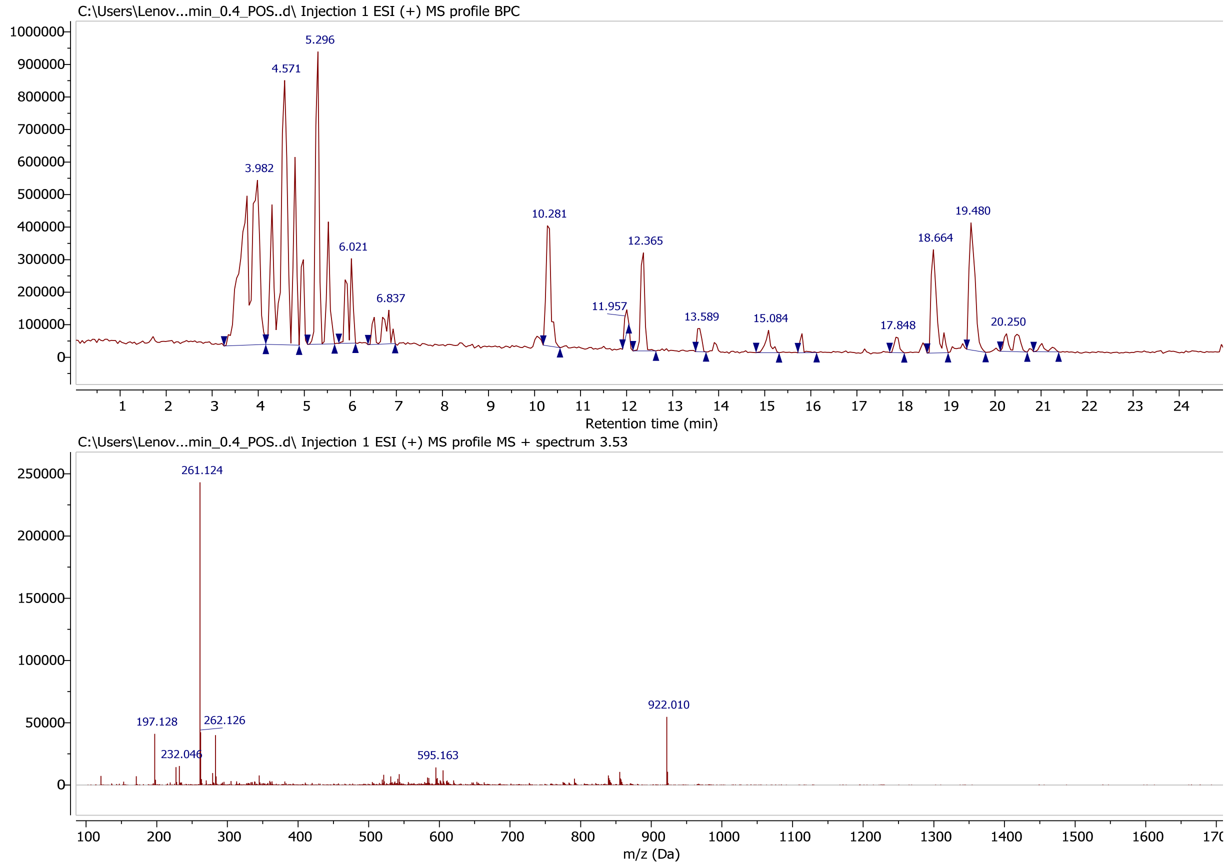

Figure S10. BPC and MS profile of Maculosin

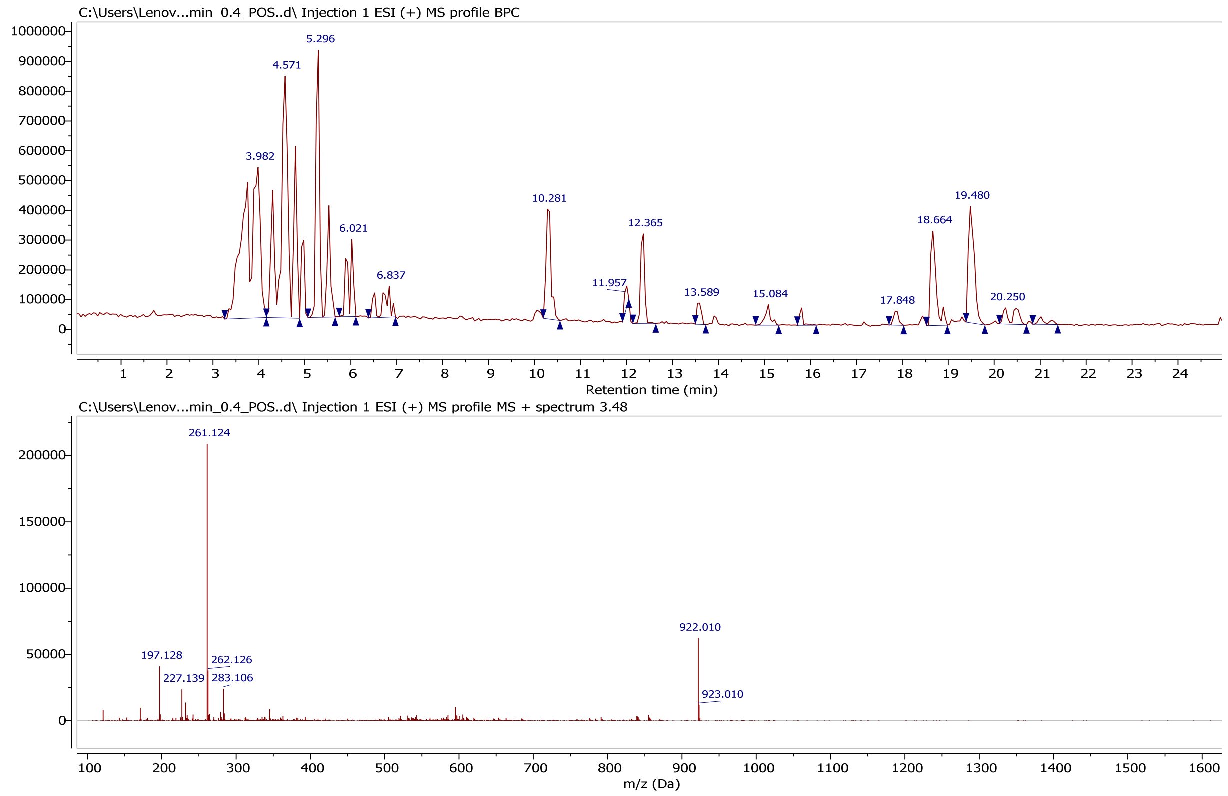

Figure S11. BPC and MS profile of Cyclo-(D-Pro-L-Val)

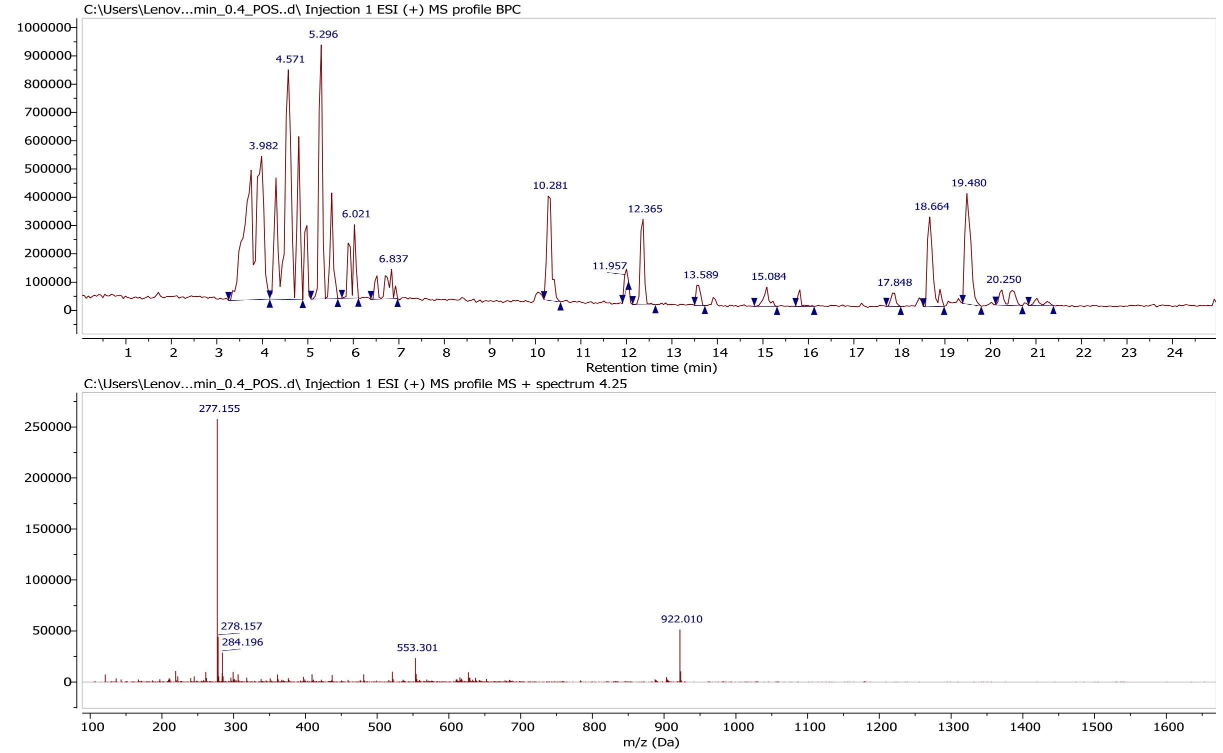

Figure S12. BPC and MS profile of Cyclo-(Tyr-Leu)

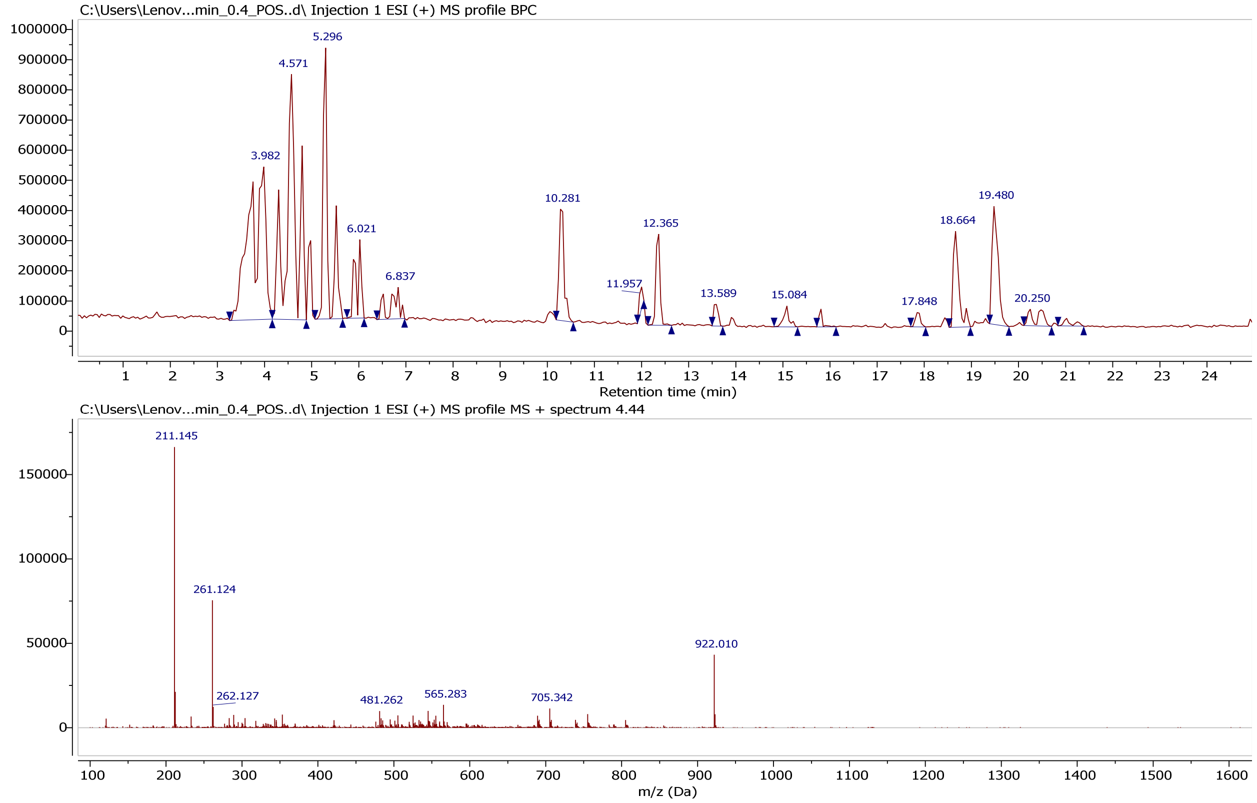

Figure S13. BPC and MS profile of Gancidin W

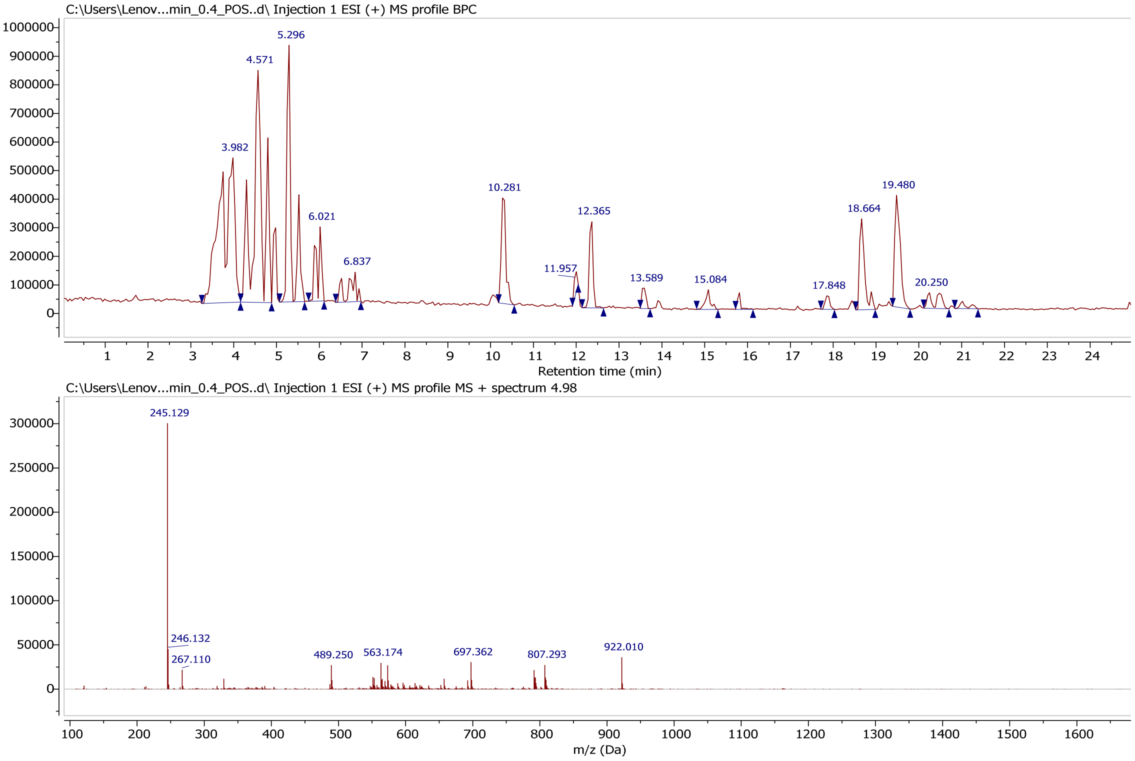

Figure S14. BPC and MS profile of Cyclo-(D-Phe-L-Pro)

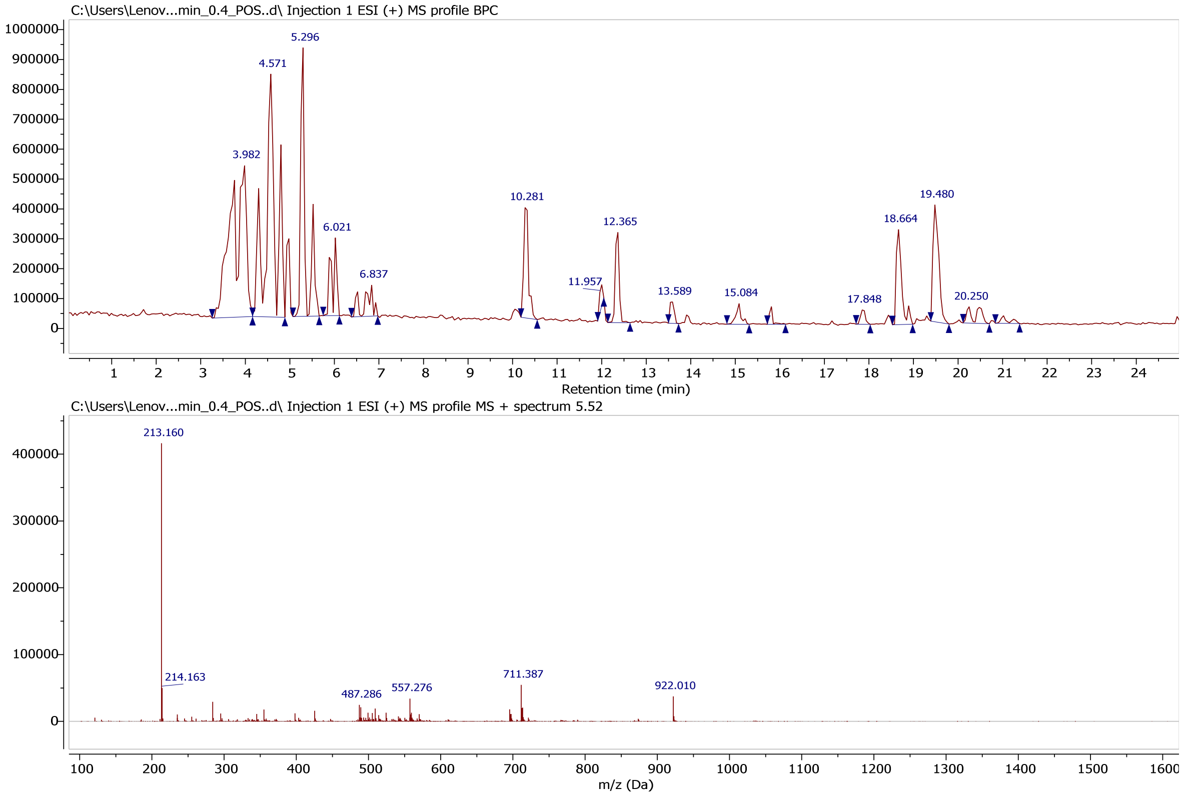

Figure S15. BPC and MS profile of Cyclo(L-val-L-Leu)

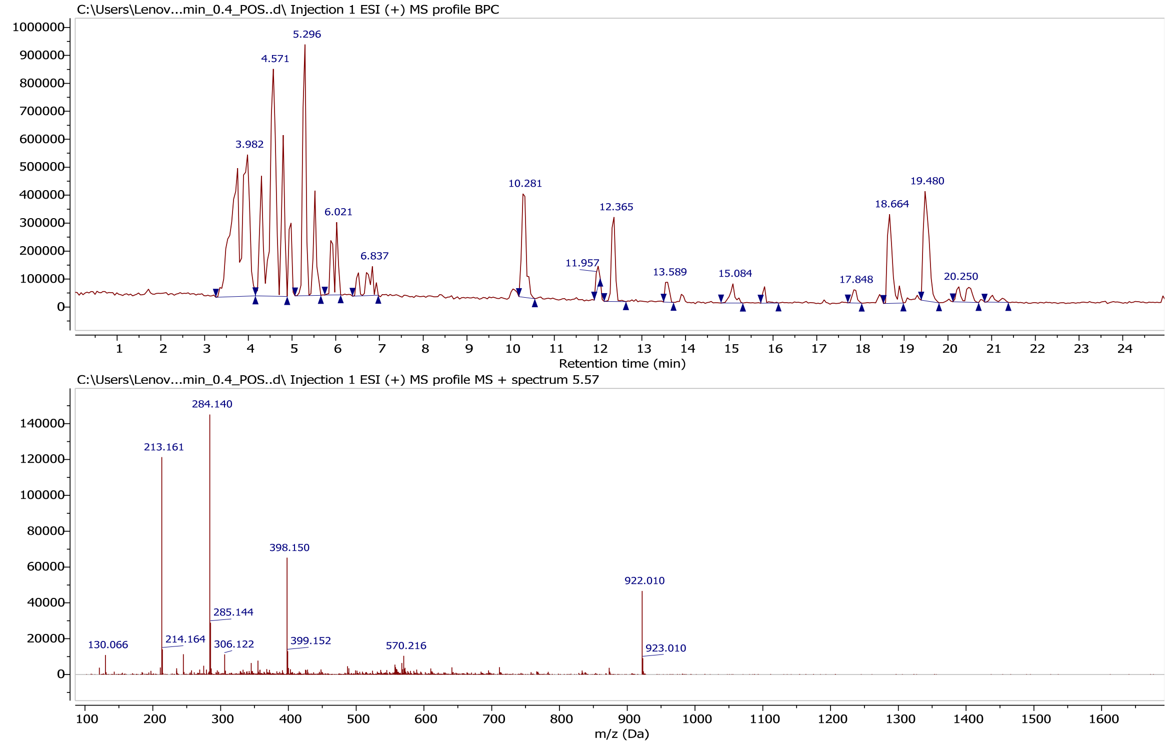

Figure S16. BPC and MS profile of Brevianamide F

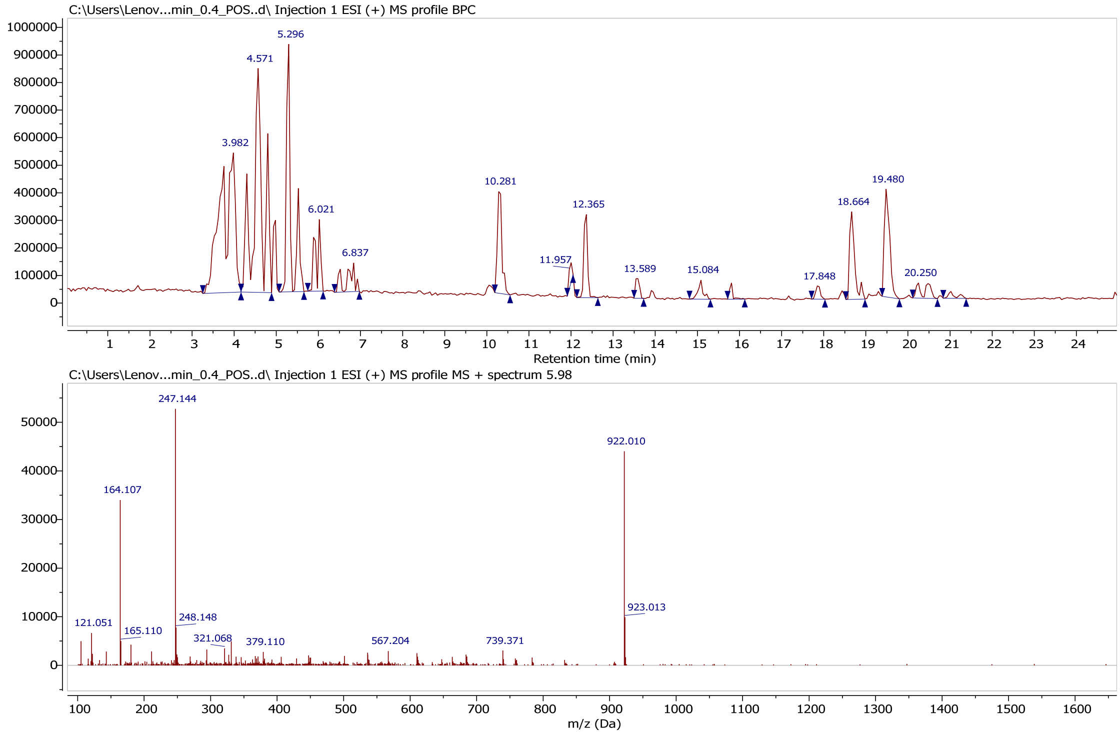

Figure S17. BPC and MS profile of Cyclo-(L-valyl-phenylalanyl)

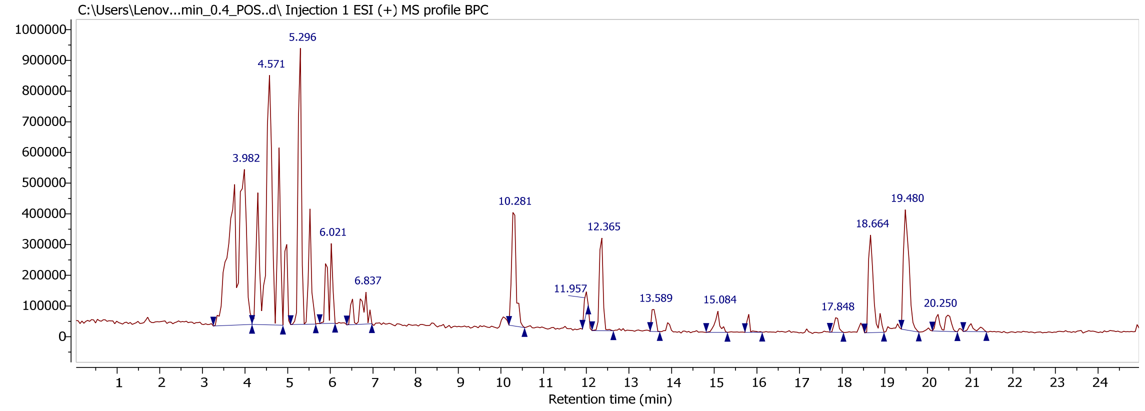

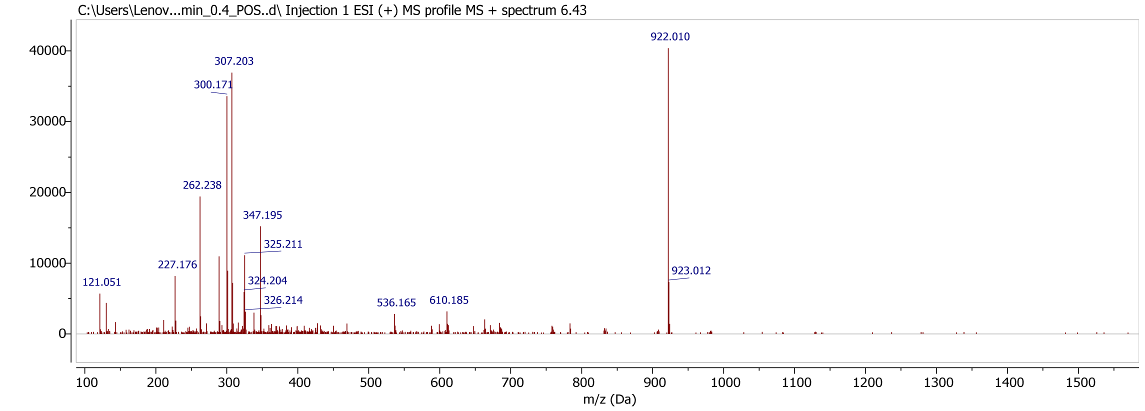

Figure S18. BPC and MS profile of Cyclo-(L-leucyl-L-leucyl)

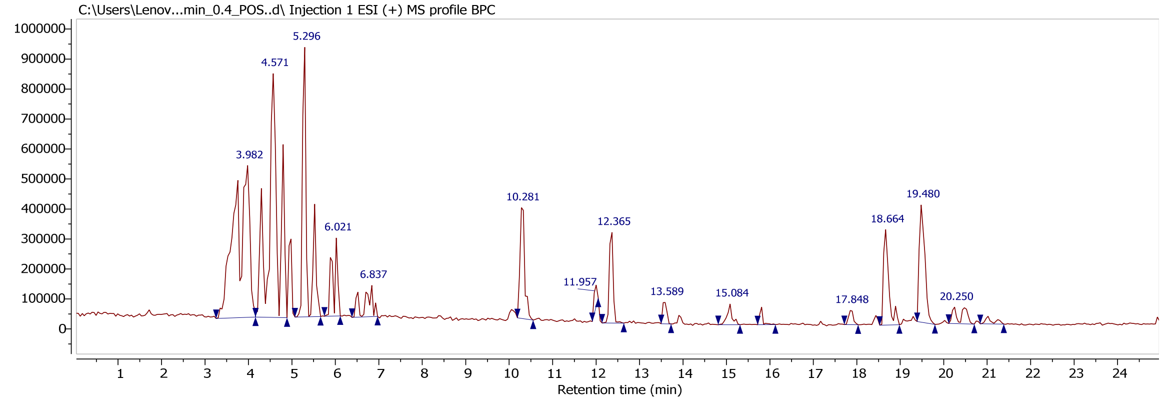

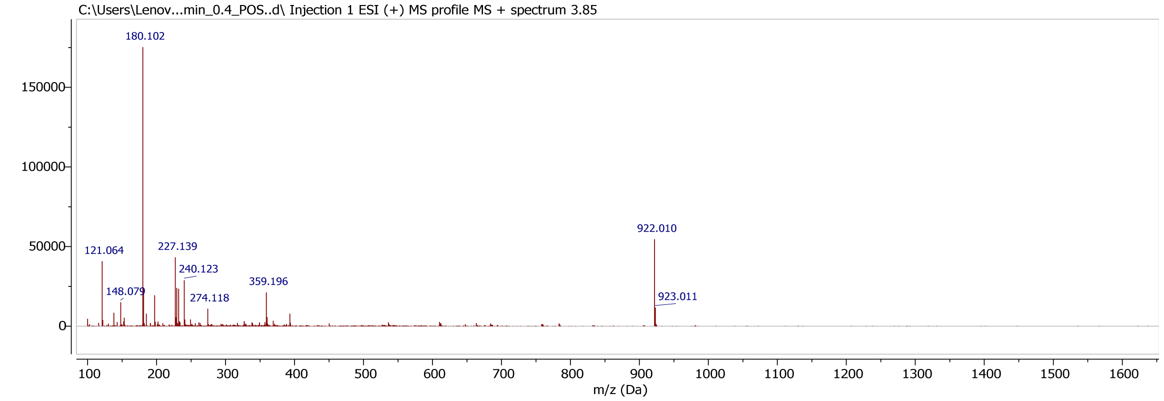

Figure S19. BPC and MS profile of *N*-Acetyltyramine

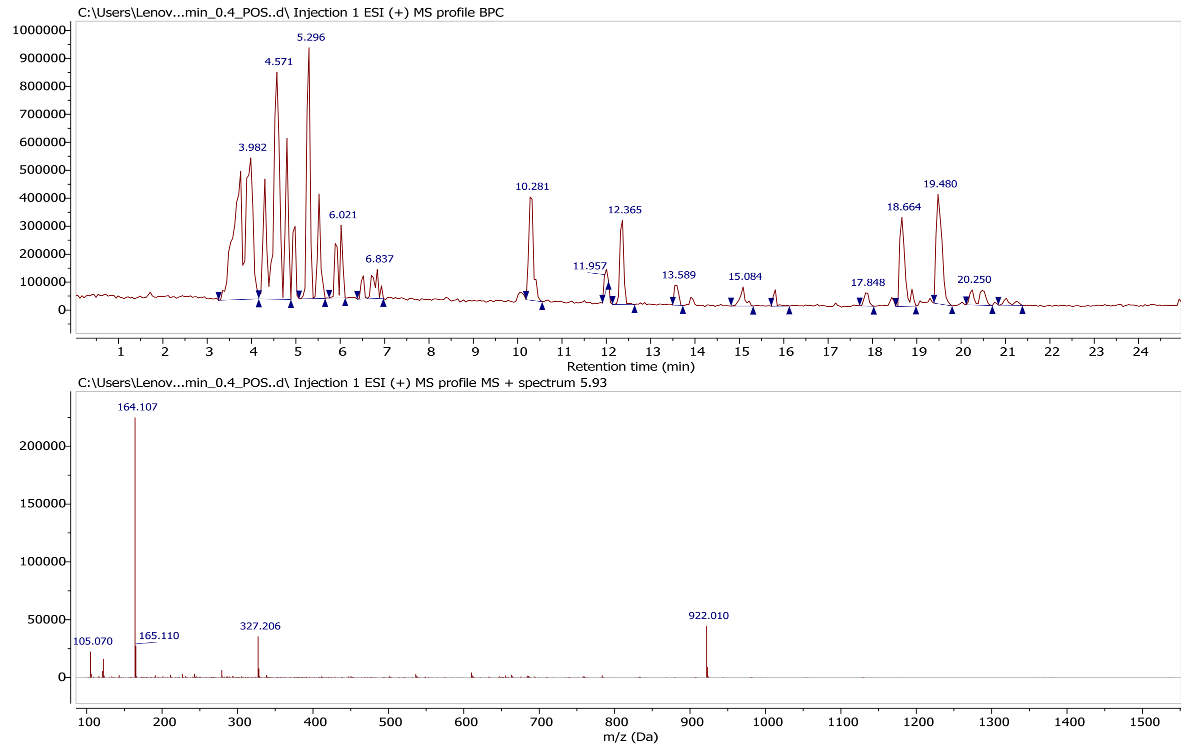

Figure S20. BPC and MS profile of *N*-Phenethylacetamide

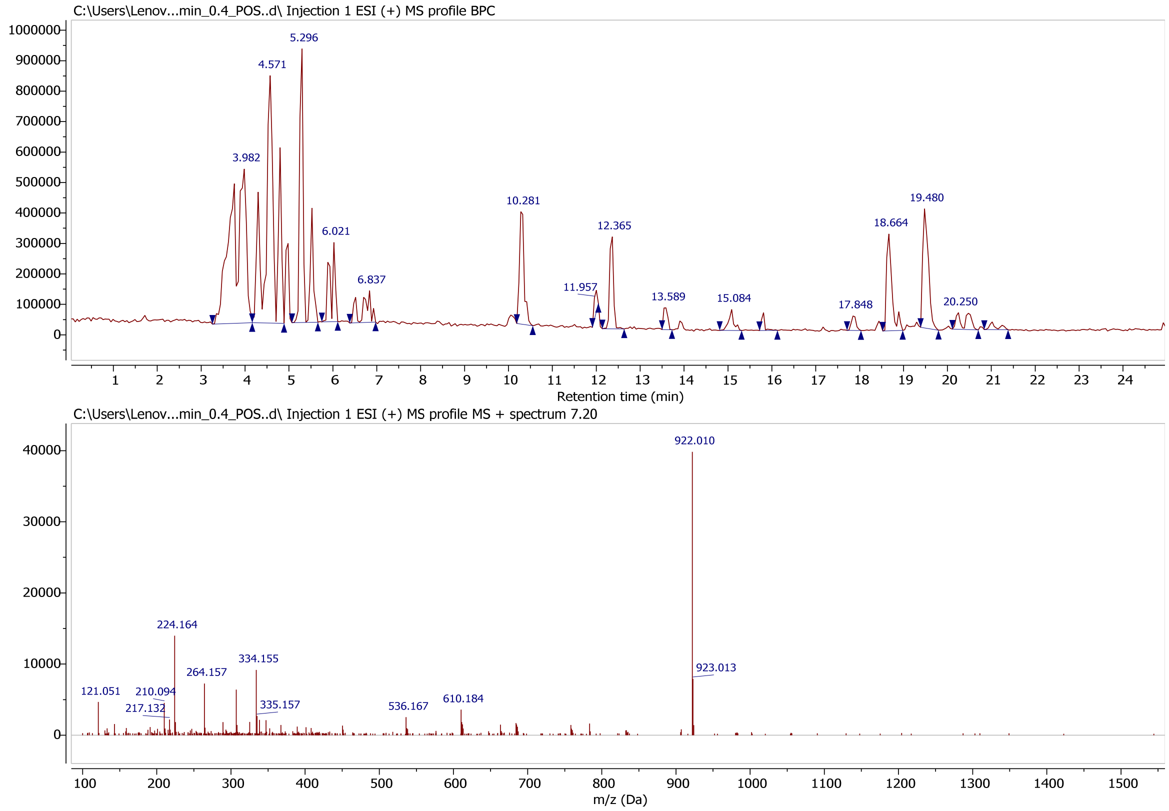

Figure S21. BPC and MS profile of Cyclo-(L-Trp-L-Phe)

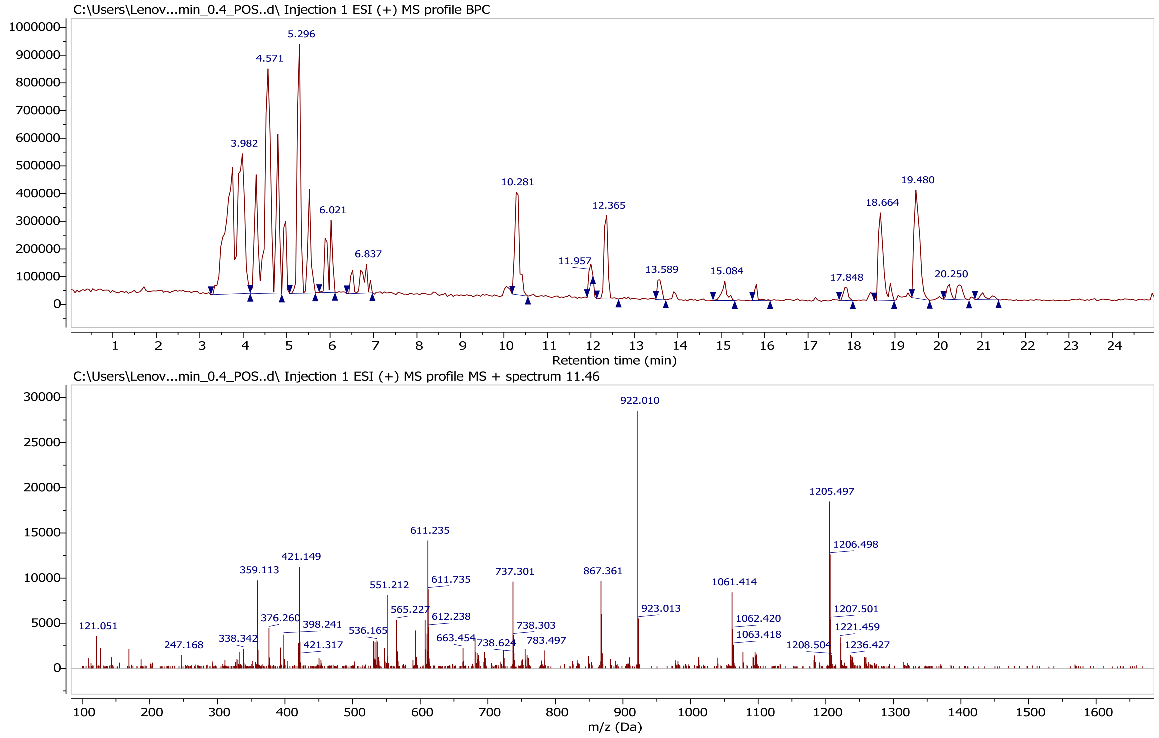

Figures S22. BPC and MS profile of Chromomycin A3

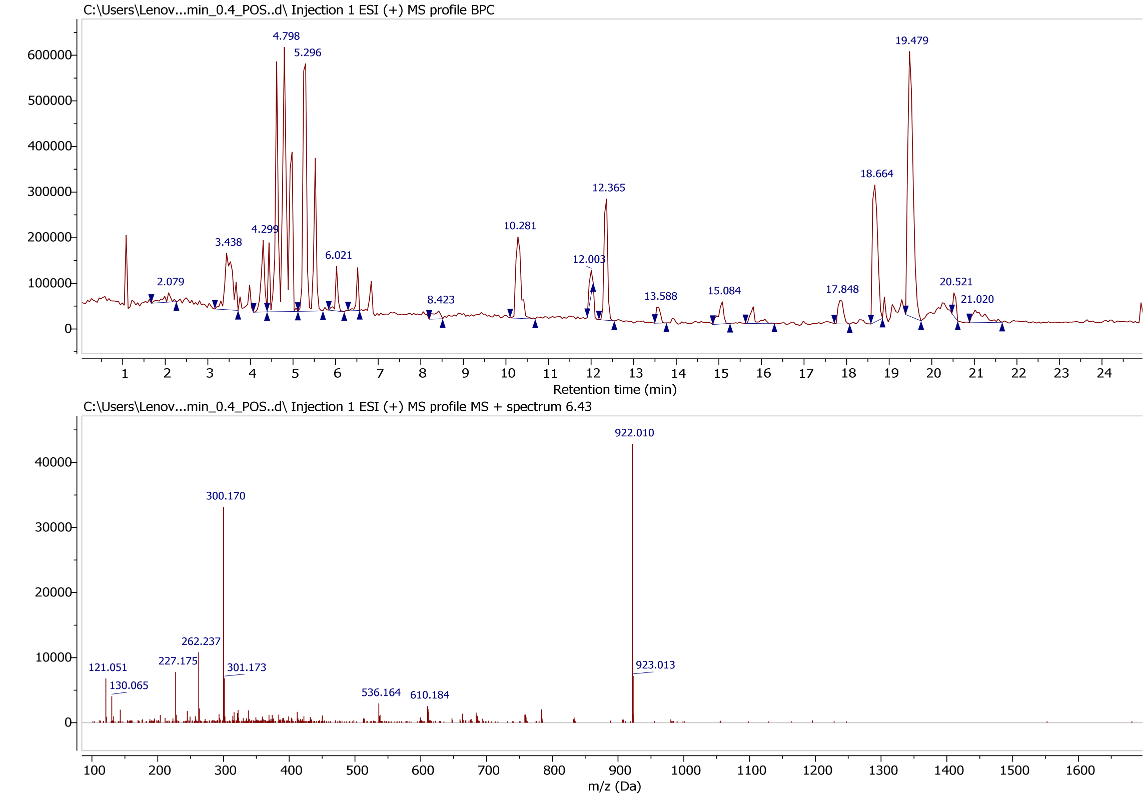

Figure S23. BPC and MS profile of Cyclo-(D-Leu-L-Trp)

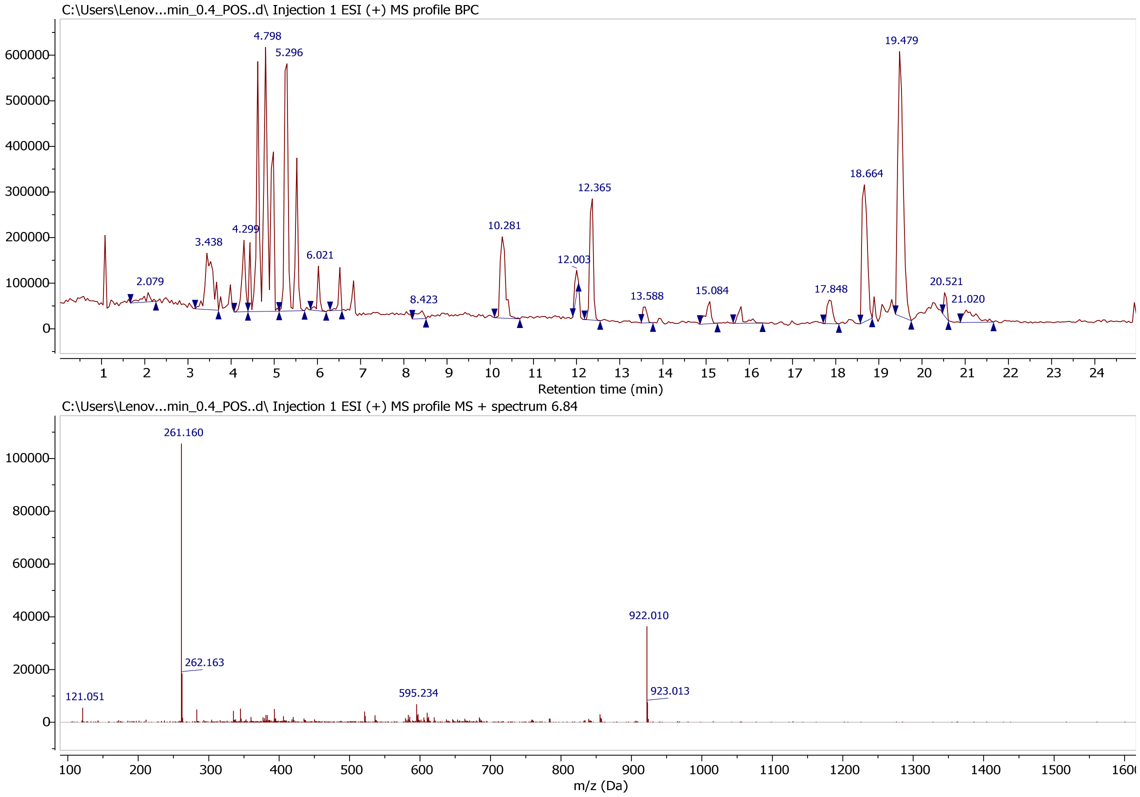

Figure S24. BPC and MS profile of Cyclo-(L-leucyl-L-phenylalanyl)

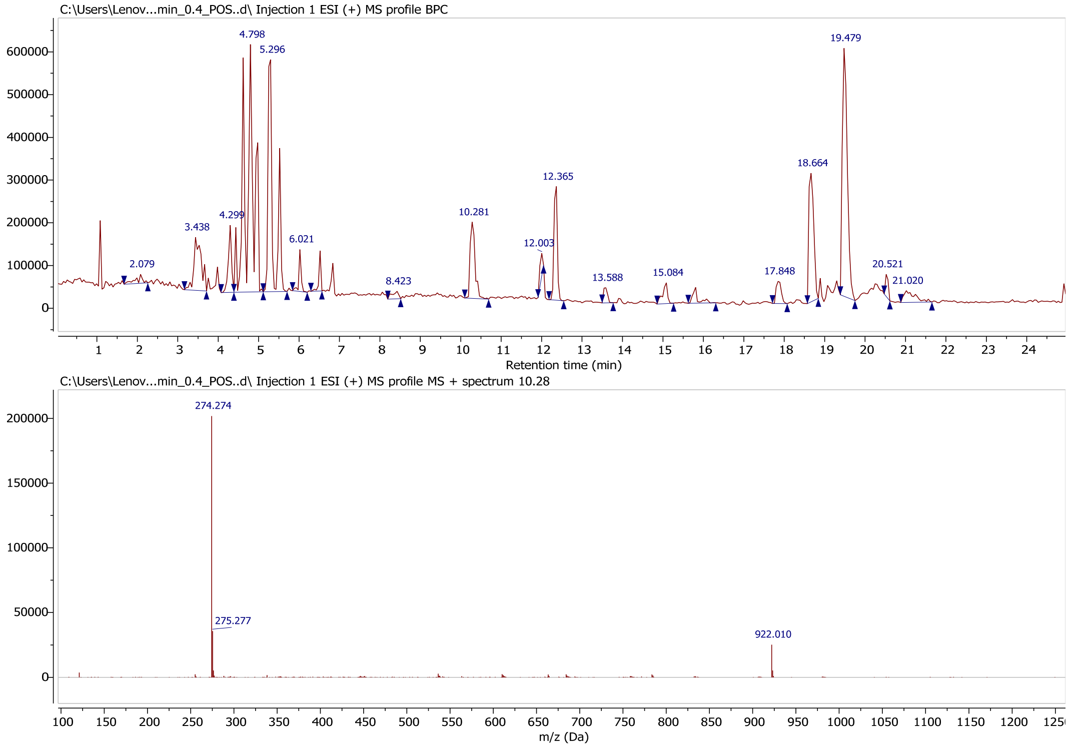

Figure S25. BPC and MS profile of *N*-Lauryldiethanolamine

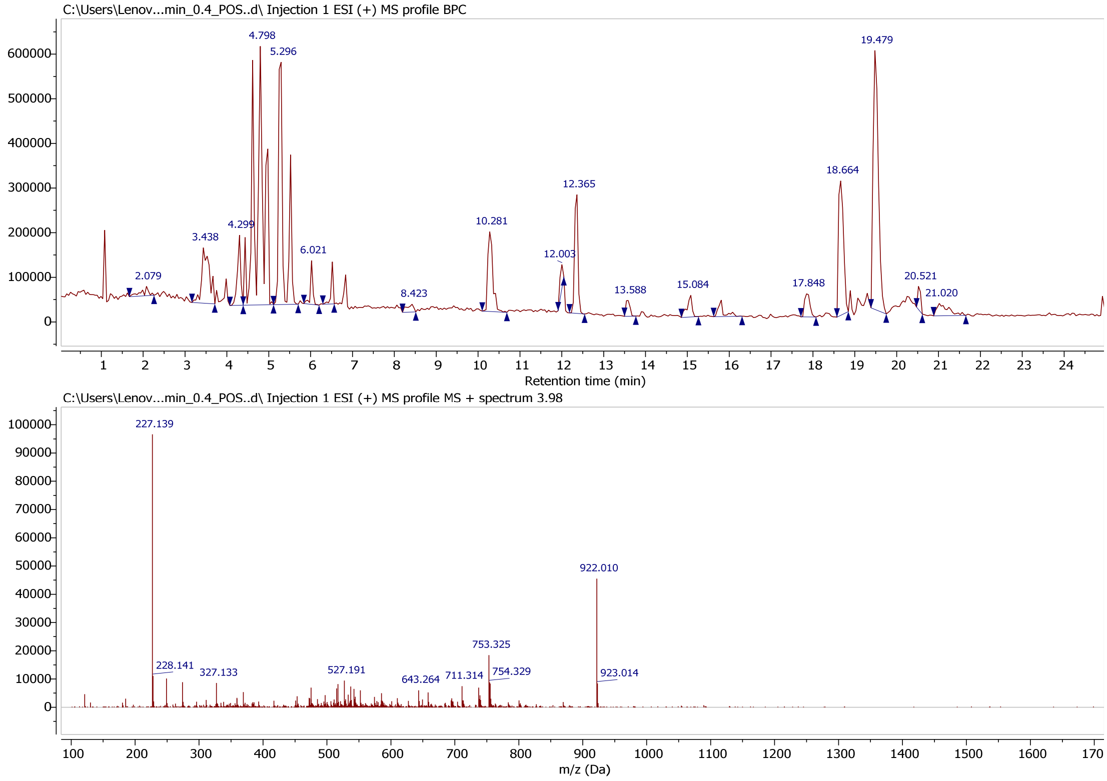

Figure S26. BPC and MS profile of Cyclo-(2-hydroxy-Pro-R-Leu)

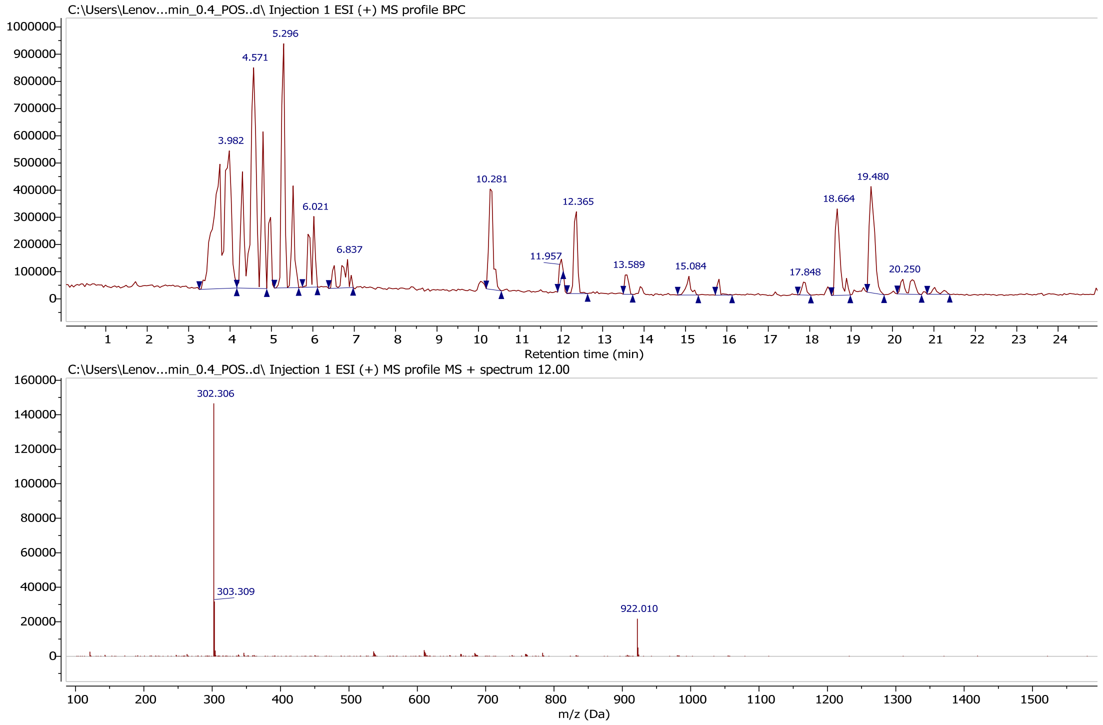

Figure S27. BPC and MS profile of Tetradecyldiethanolamine

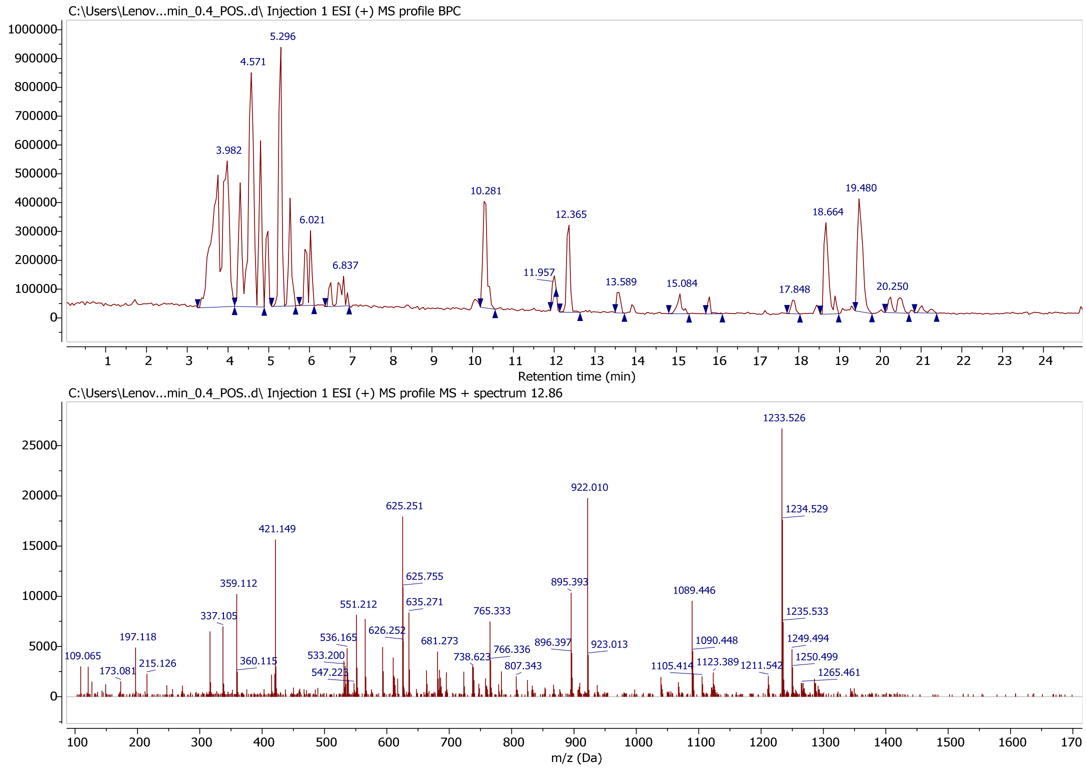

Figure S28. BPC and MS profile of Chromomycin A2

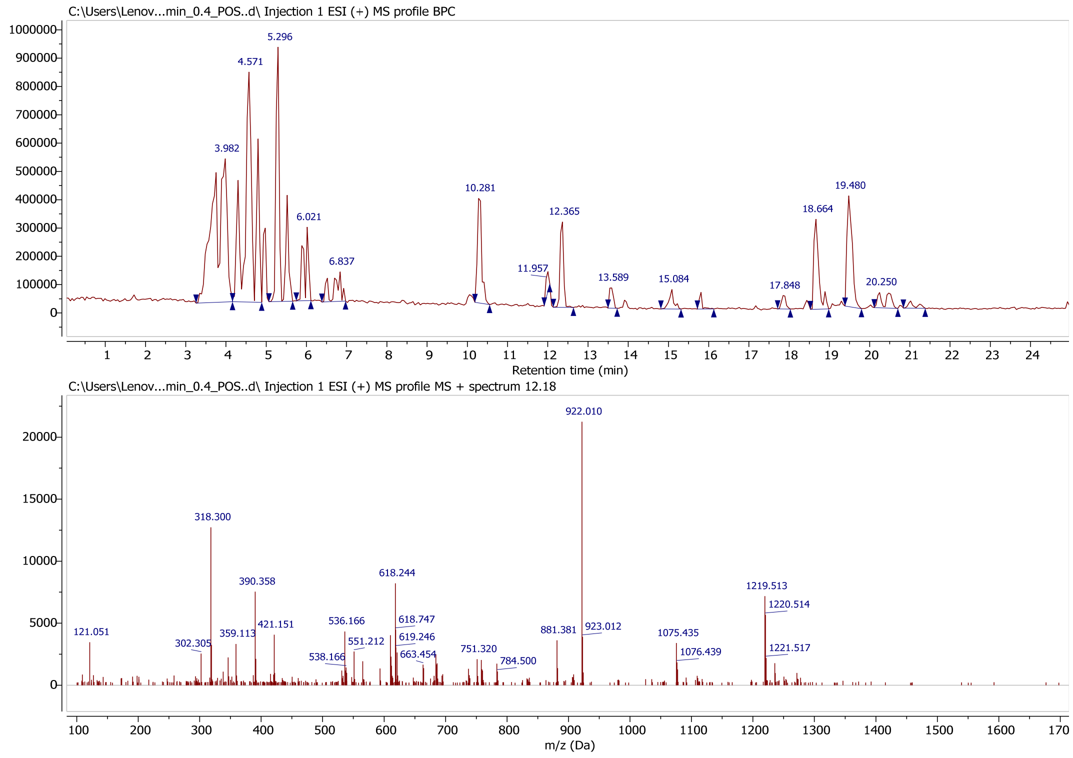

Figure S29. BPC and MS profile of Demethlychromomycin A2

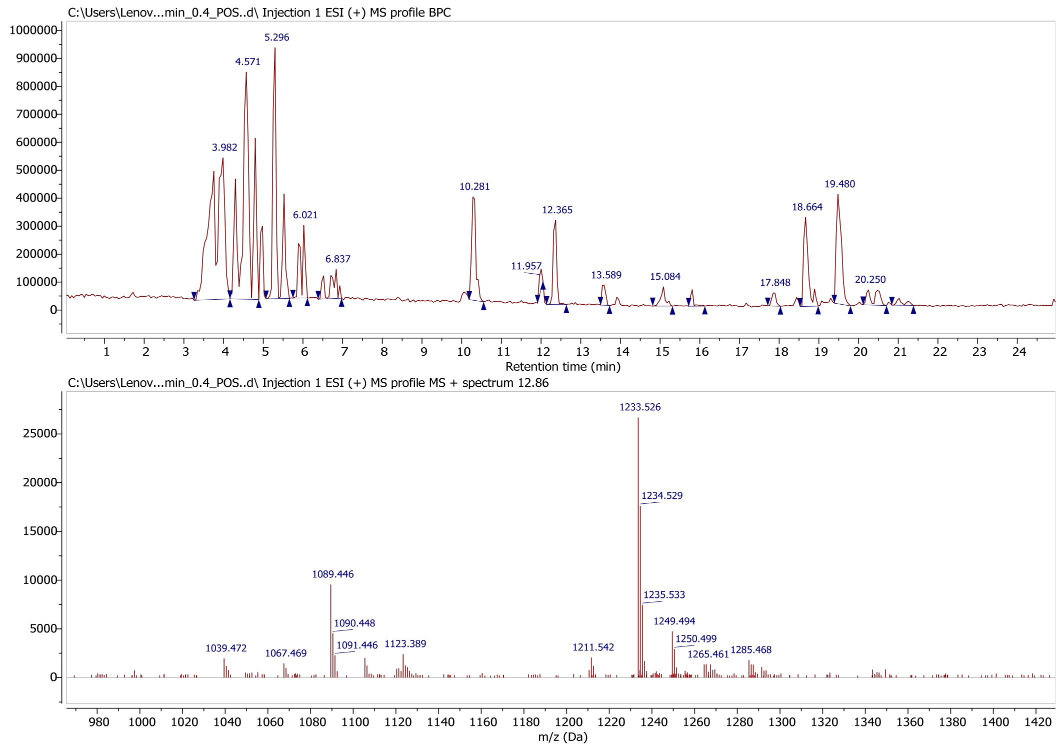

Figure S30. BPC and MS profile of Chromomycin A2-1

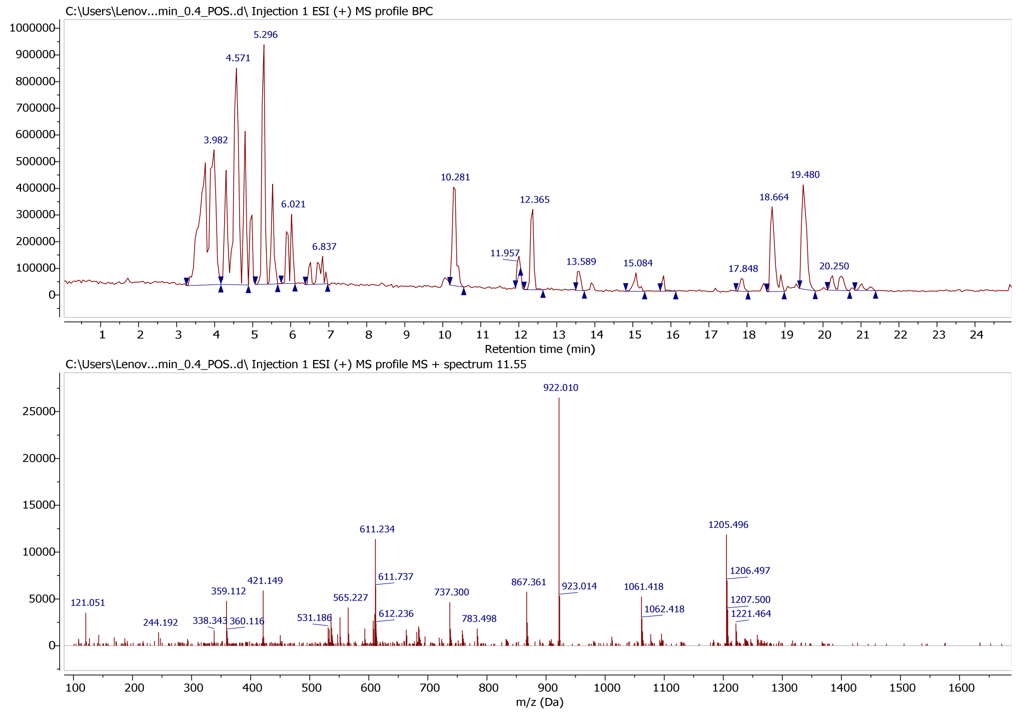

Figure S31. BPC and MS profile of Chromomycin A3-1

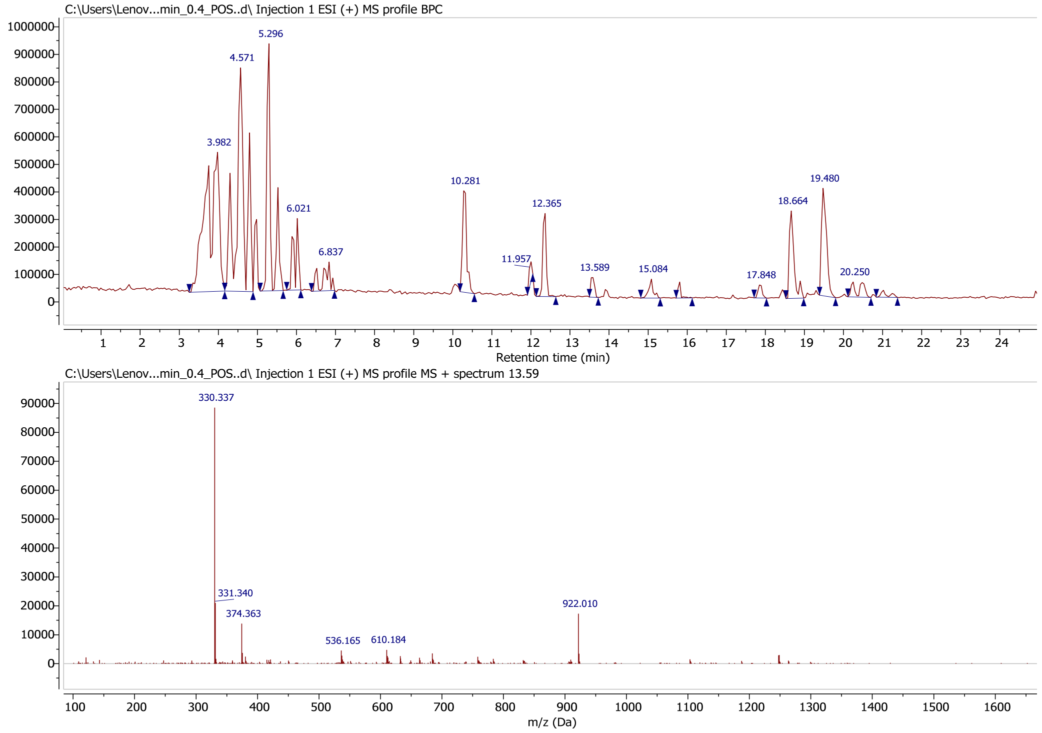

Figure S32. BPC and MS profile of *N*-Hexadecyl diethanolamine

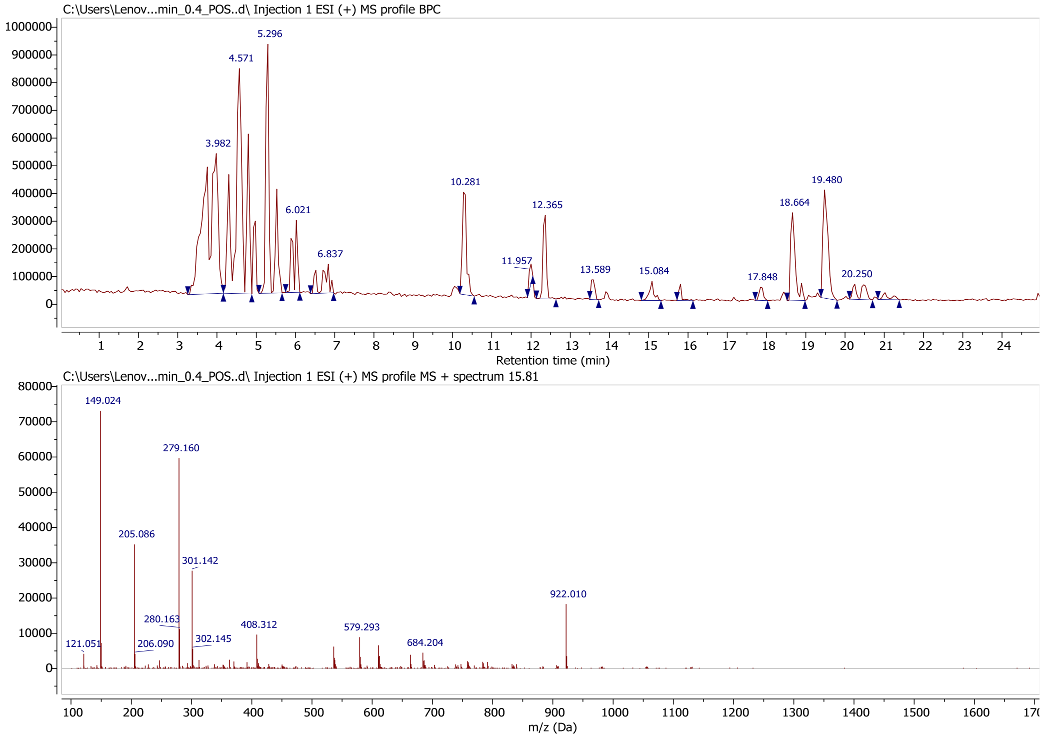

Figure S33. BPC and MS profile of Dibutyl Phthalate

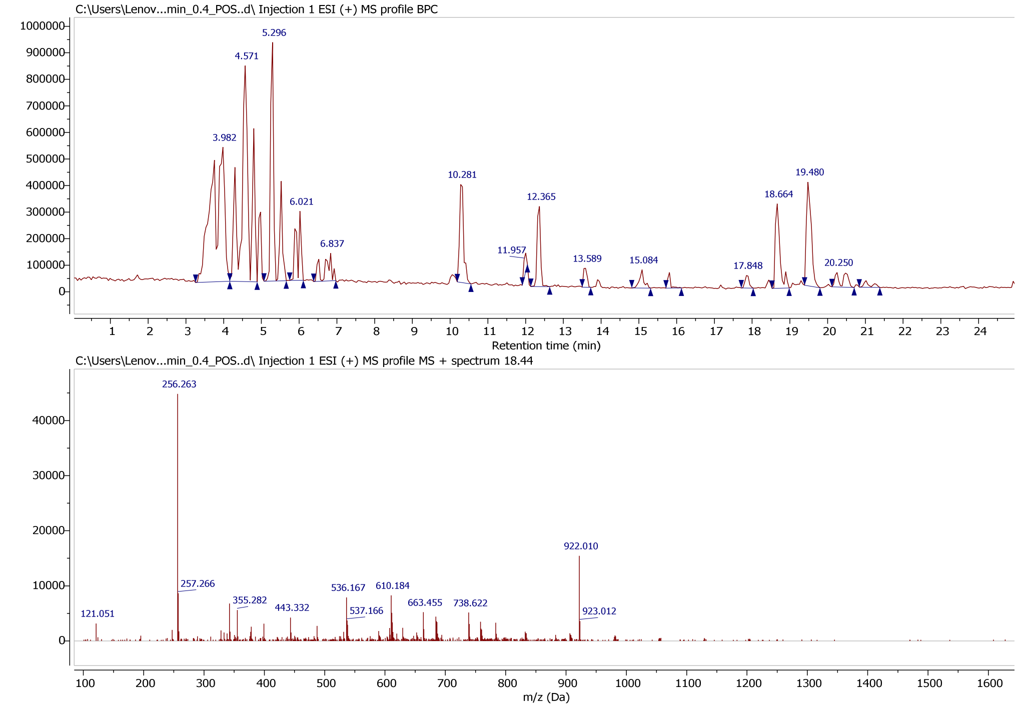

Figure S34. BPC and MS profile of Hexadecanamide

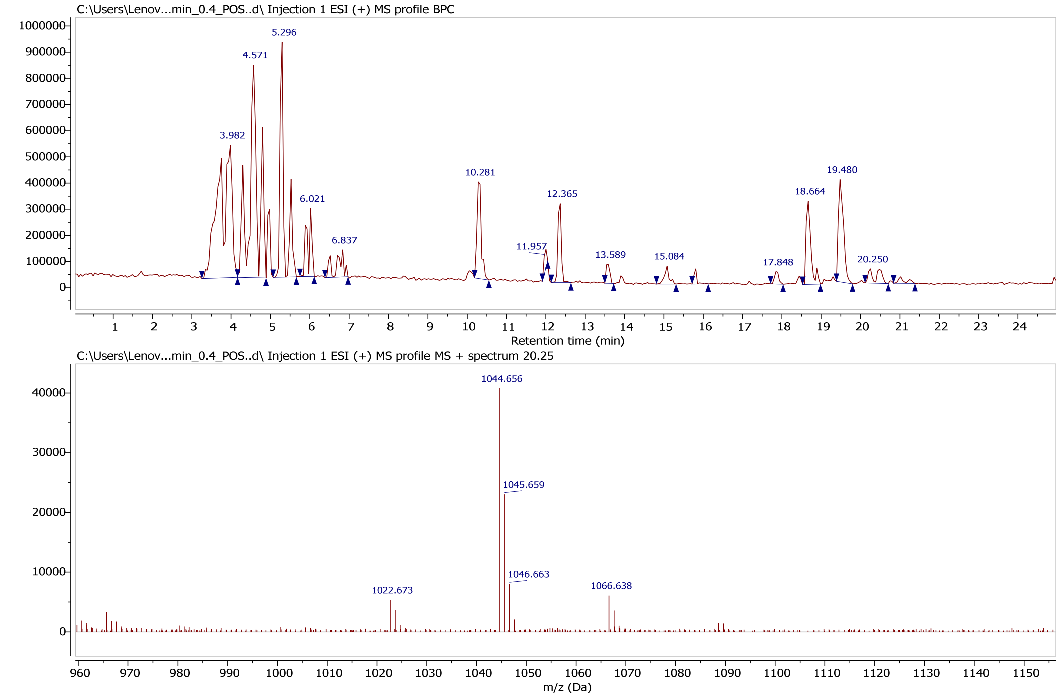

Figure S35. BPC and MS profile of Surfactin B

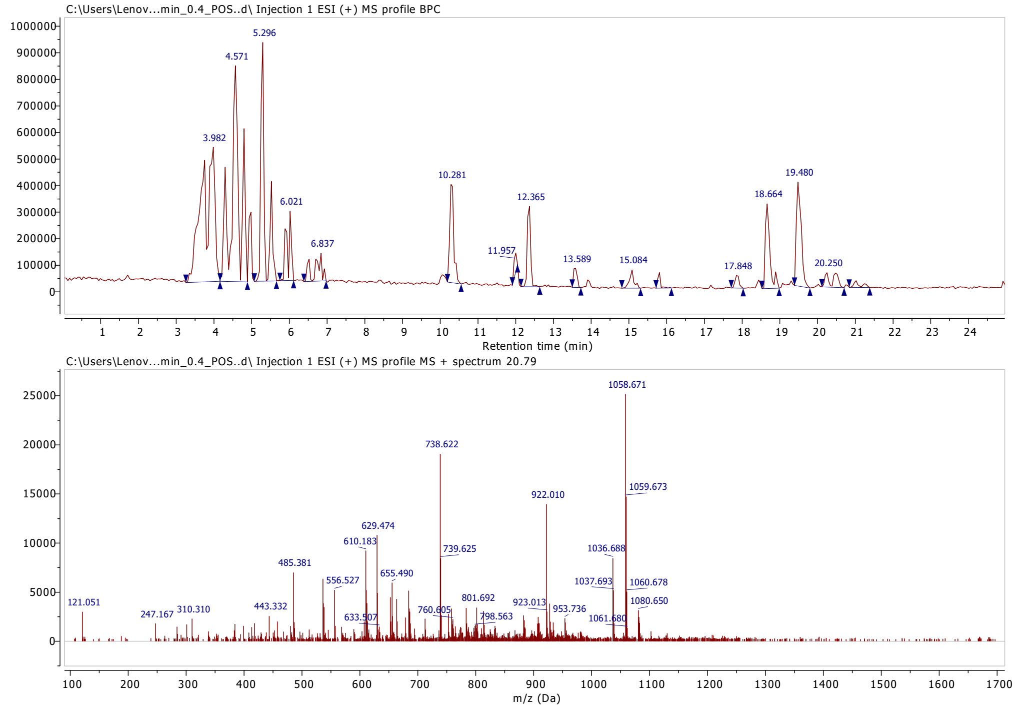

Figure S36. BPC and MS profile of Surfactin C

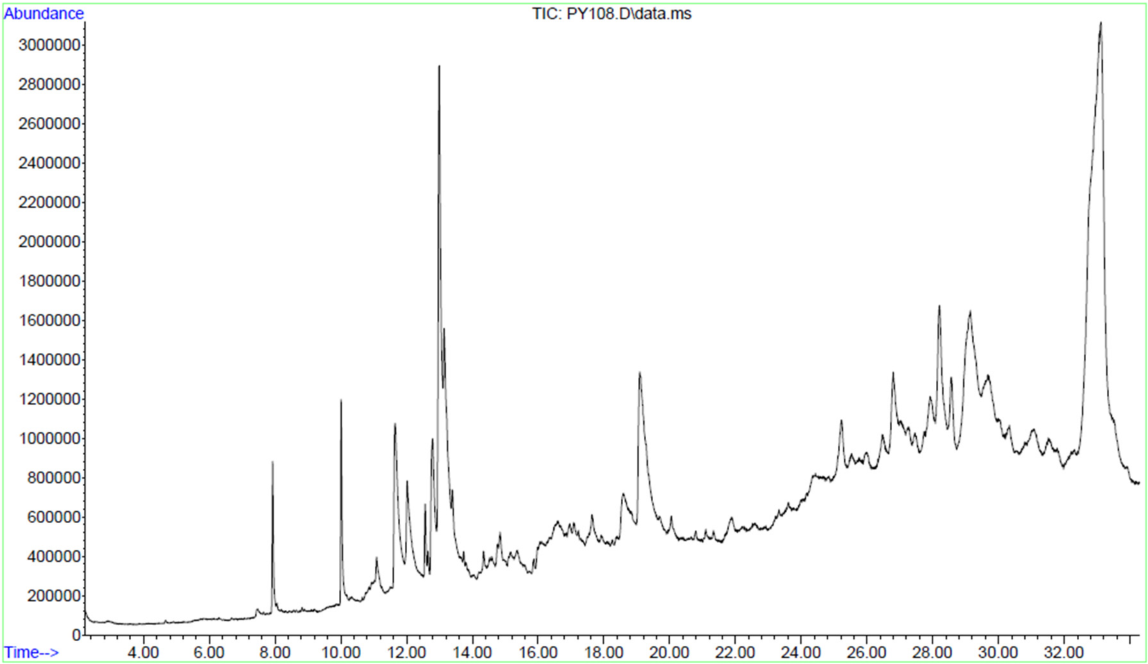

Figure S37. GC-MS chromatogram obtained in EA extracts of *Streptomyces* sp. PY108.

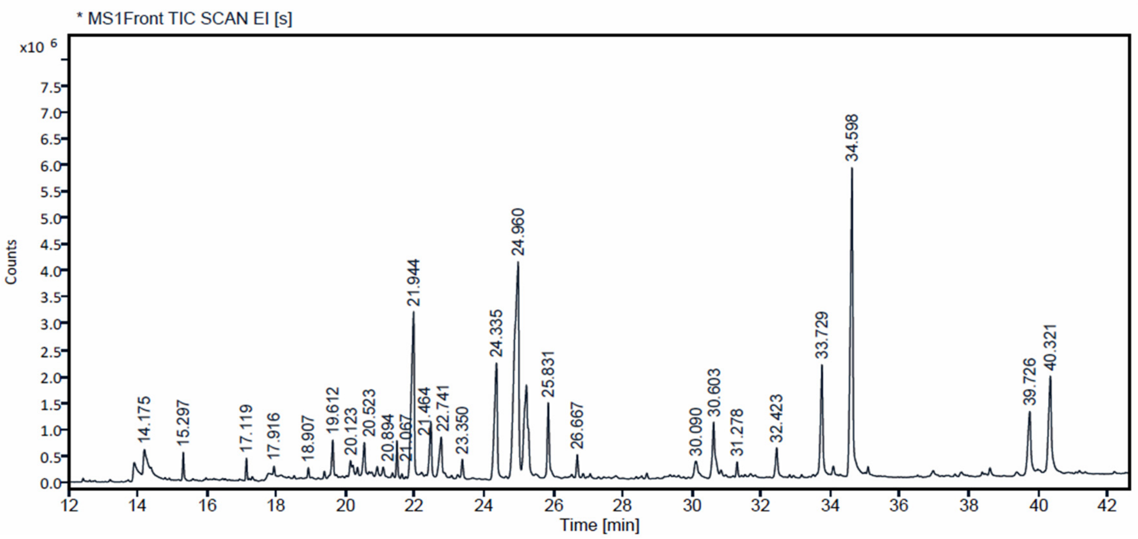

Figure S38. GC-MS chromatogram obtained in EA extracts of *Streptomyces* sp. PY109

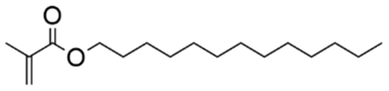

$C_{17}H_{32}O_2$
2-Propenoic acid

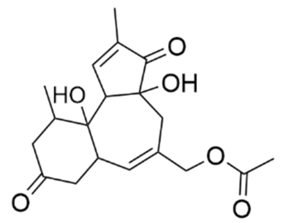

$C_{19}H_{24}O_6$
Benz[e]azulene-3,8-dione,

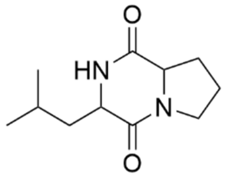

$C_{11}H_{18}N_2O_2$
Pyrrolo[1,2-a]pyrazine-1,4-dione, hexahydro-3-(2-methylpropyl)-

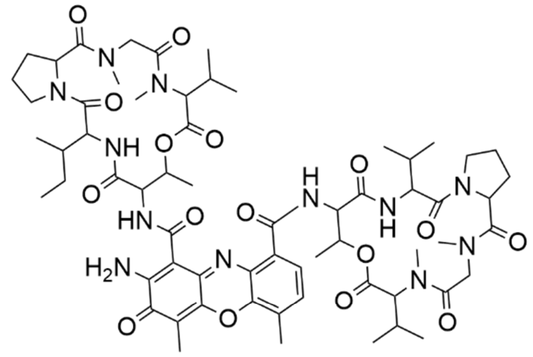

$C_{63}H_{88}N_{12}O_{16}$
Actinomycin C2

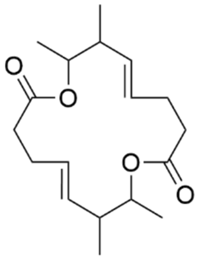

$C_{18}H_{28}O_4$
1,9-Dioxacyclohexadeca-4,13-diene -2-10-dione, 7,8,15,16-tetramethyl-

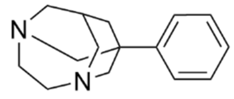

$C_{15}H_{20}N_2$
1-Phenyl-3,6-diazahomoadamantane

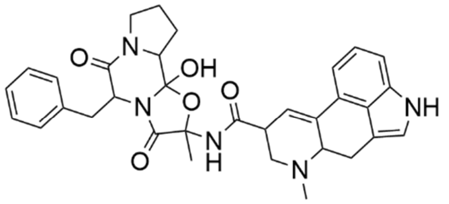

$C_{33}H_{35}N_5O_5$
Ergotaman-3',6',18-trione,

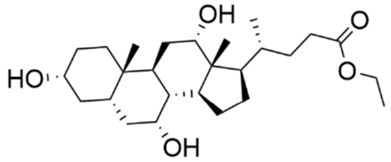

$C_{26}H_{44}O_5$
Ethyl iso-allocholate

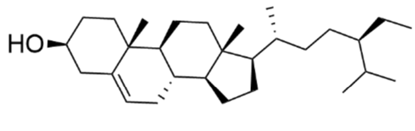

$C_{29}H_{50}O$
γ-Sitosterol

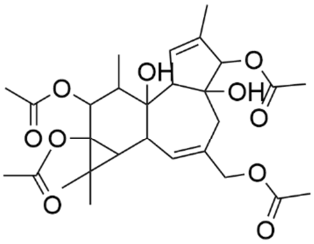

$C_{28}H_{38}O_{10}$
1H-Cyclopropa[3,4]benz[1,2-e]azulene-4a,5,7b,9,9a(1aH)-pentol

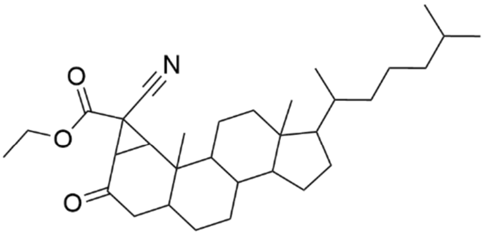

$C_{32}H_{49}NO_3$
3'H-Cycloprop(1,2)-5-cholest-1-en-3-one

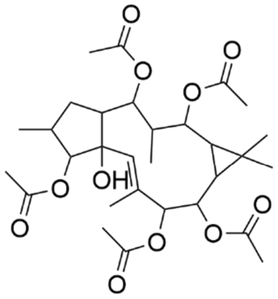

$C_{30}H_{44}O_{11}$
7aH-Cyclopenta[a]cyclopropa[f]cycloundecene-2,4,7,7a,10,11-hexol

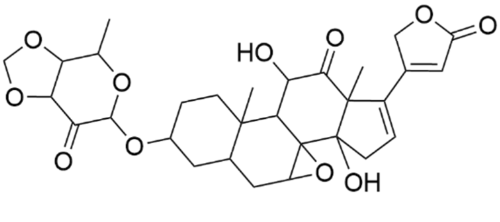

$C_{30}H_{36}O_{11}$
Carda-16,20(22)-dienolide

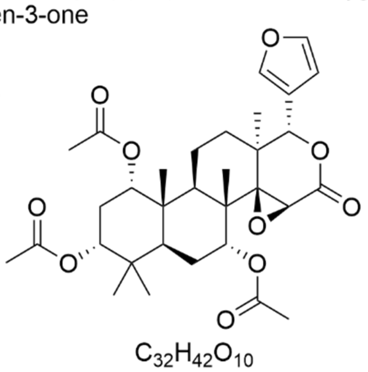

$C_{32}H_{42}O_{10}$
D-Homo-24-nor-17-oxachola-20,22-dien16-one

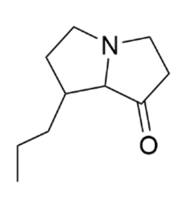

$C_{10}H_{17}NO$
Pyrrolizin-1-one, 7-propyl-

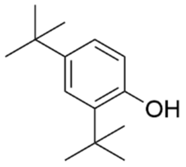

$C_{14}H_{22}O$
2,4-Di-tert-butylphenol

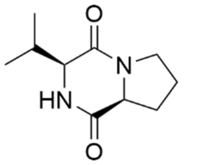

$C_{10}H_{16}N_2O_2$
cyclo(L-Pro-L-Val)

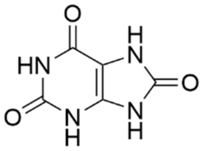

$C_5H_4N_4O_3$
Uric Acid

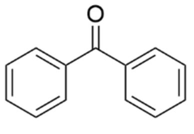

$C_{13}H_{10}O$
Benzophenone

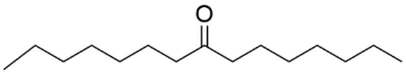

$C_{15}H_{30}O$
8-Pentadecanone

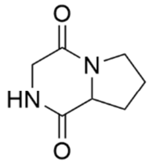

$C_7H_{10}N_2O_2$
Pyrrolo[1,2-a]pyrazine-1,4-dione, hexahydro-

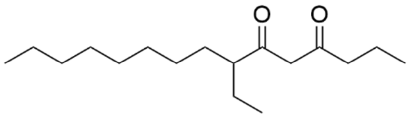

$C_{17}H_{32}O_2$
7-Ethylpentadecane-4,6-dione

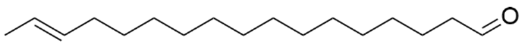

$C_{17}H_{32}O$
E-15-Heptadecenal

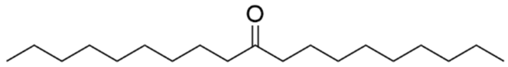

$C_{19}H_{38}O$
10-Nonadecanone

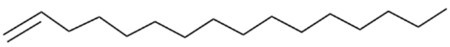

$C_{16}H_{32}$
Cetene

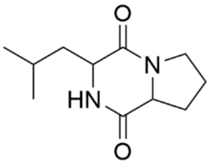

$C_{11}H_{18}N_2O_2$
Pyrrolo[1,2-a]pyrazine-1,4-dione, hexahydro-3-(2-methylpropyl)-

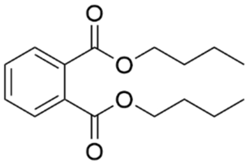

$C_{16}H_{22}O_4$
Dibutyl Phthalate

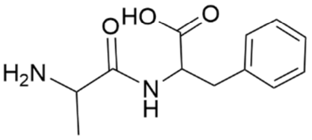

$C_{12}H_{16}N_2O_3$
dl-Alanyl-*dl*-phenylalanine

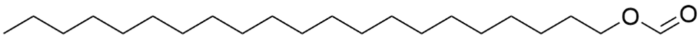

$C_{22}H_{44}O_2$
1-Henicosyl formate

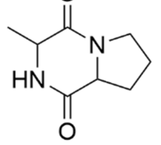

$C_8H_{12}N_2O_2$
1,4-diazabicyclo[4.3.0]nonan-2,5-dione,3-methyl

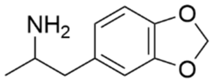

$C_{10}H_{13}NO_2$
3,4-Methylenedioxyamphetamine

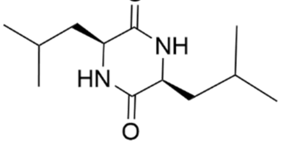

$C_{12}H_{22}N_2O_2$
2,5-Piperazinedione, 3,6-bis(2-methylpropyl)-

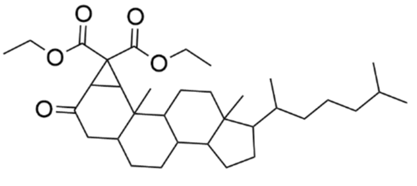

$C_{34}H_{54}O_5$
3'H-Cycloprop(1,2)-5α-cholest-1-en-3-one,1',1'-dicarboethoxy-1β,2β-dihydro

Figure S39. Chemical structures of identified compounds in EA extracts of *Streptomyces* species PY108 and PY109 through GC-MS analysis.

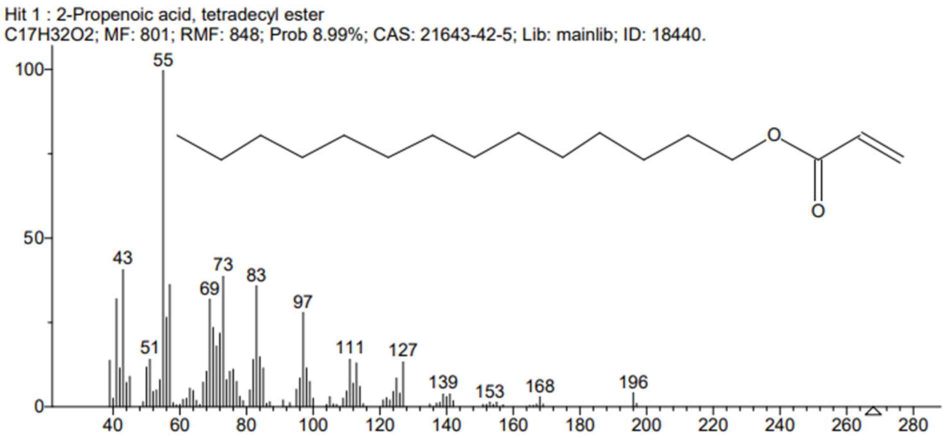

Figure S40. GC-MS profile of 2-Propenoic acid

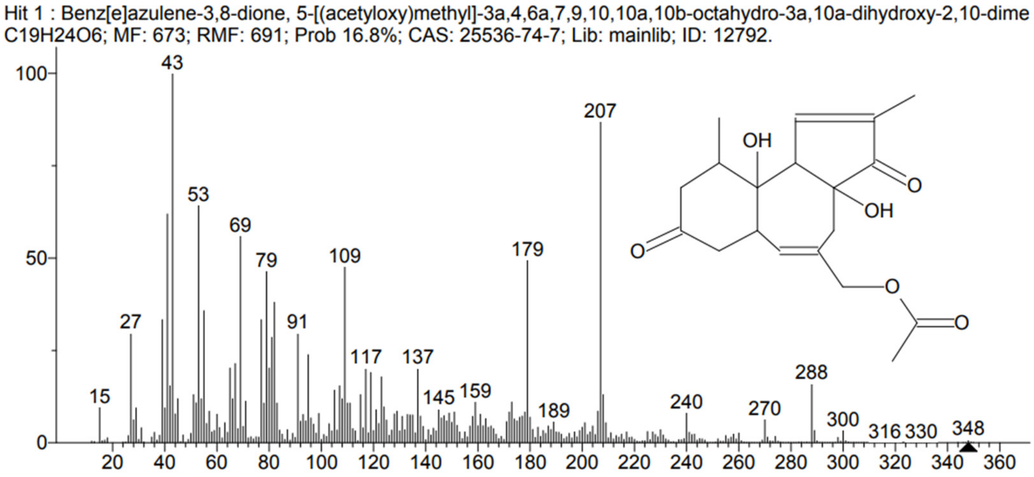

Figure S41. GC-MS profile of Benz[e]azulene-3,8-dione

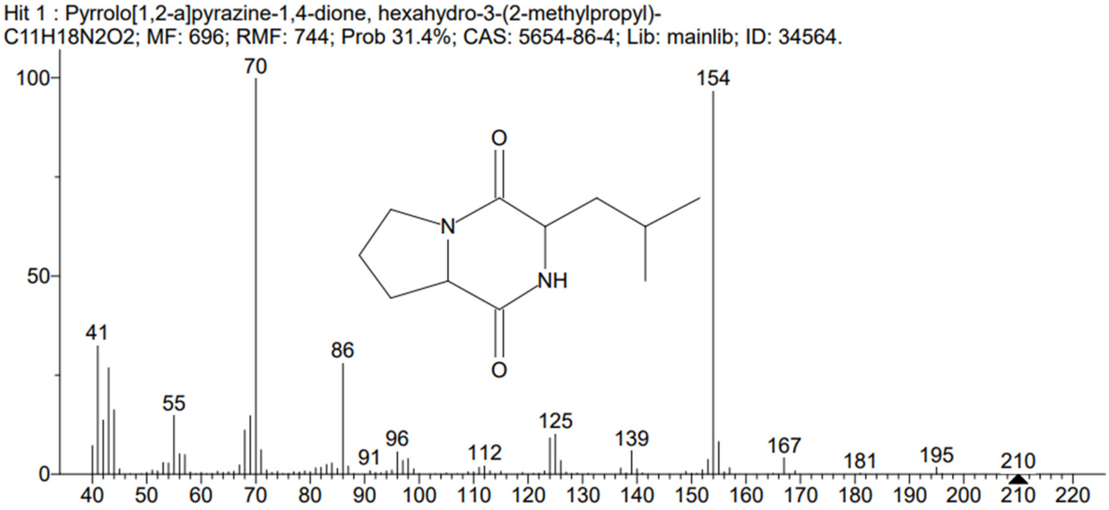

Figure S42. GC-MS profile of Pyrrolo[1,2-a]pyrazine-1,4-dione, hexahydro-3-(2-methyl propyl)-

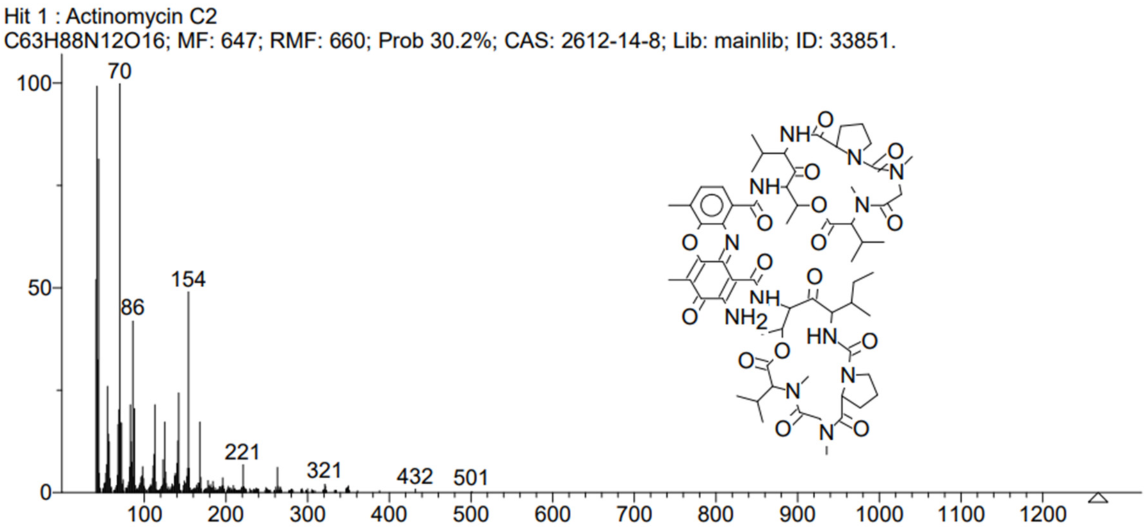

Figure S43. GC-MS profile of Actinomycin C2

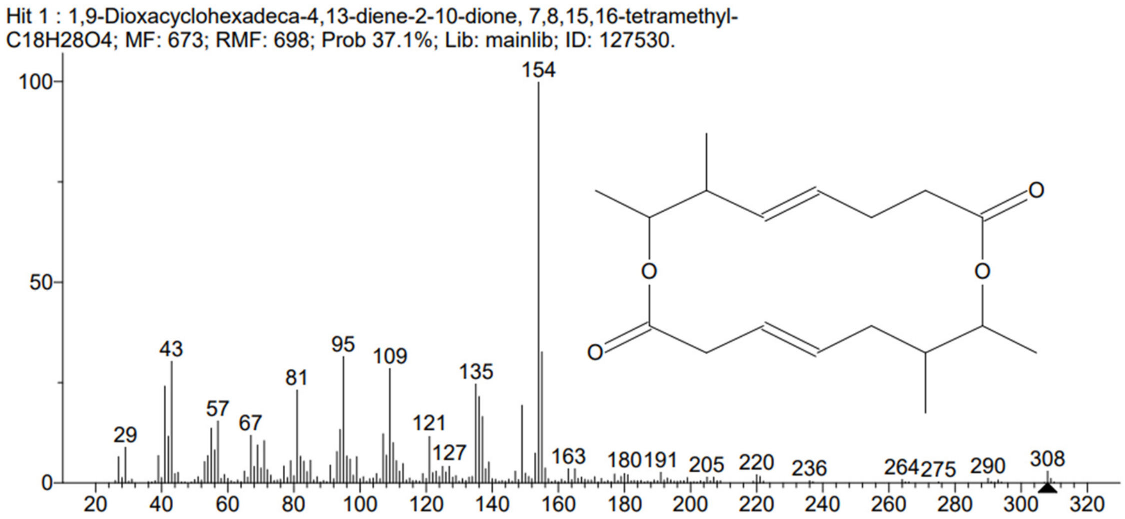

Figure S44. GC-MS profile of 7,8,15,16-tetramethyl-1,9-dioxacyclohexadeca-4,13-diene-2-10-dione

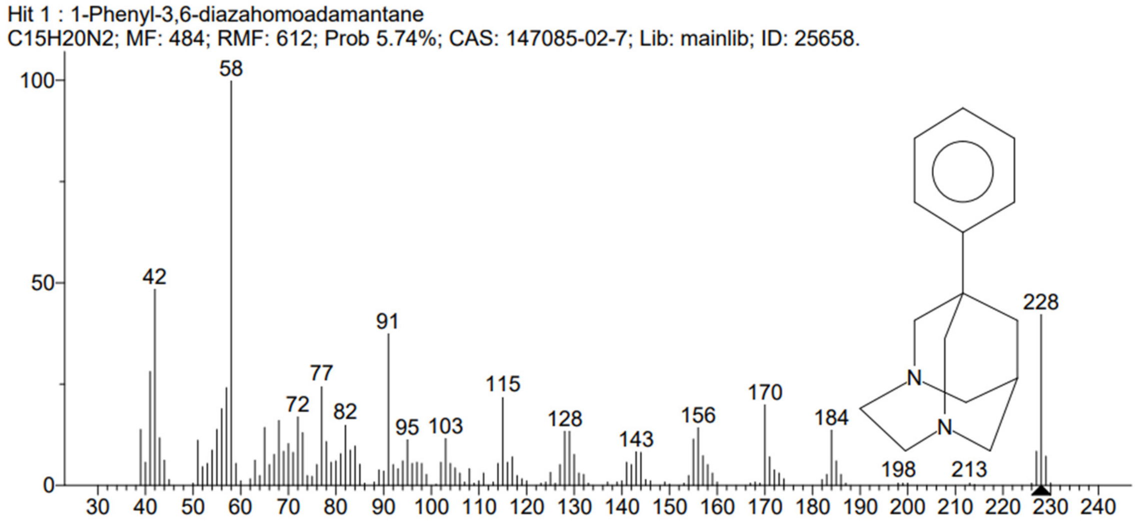

Figure S45. GC-MS profile of 1-Phenyl-3,6-diazahomoadamantane

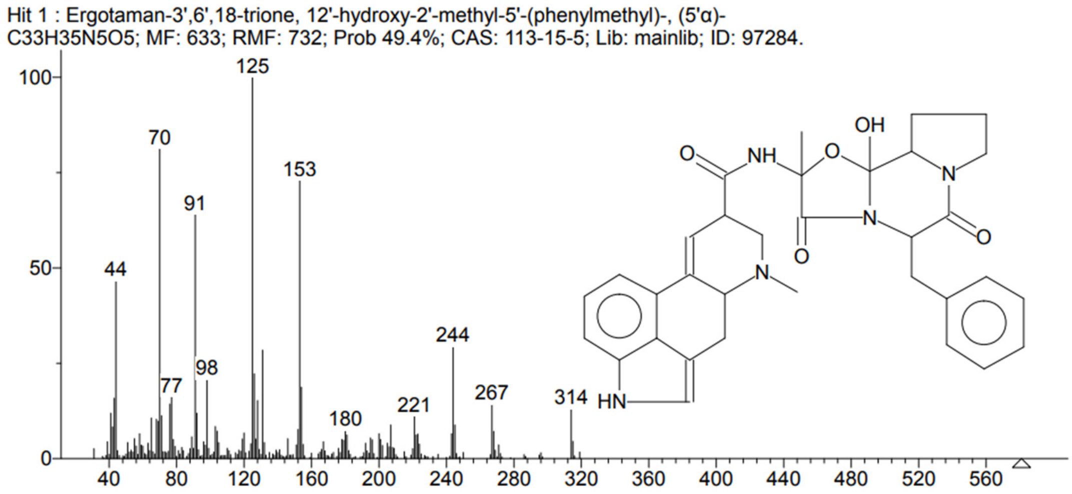

Figure S46. GC-MS profile of ergotaman-3', 6', 18-trione

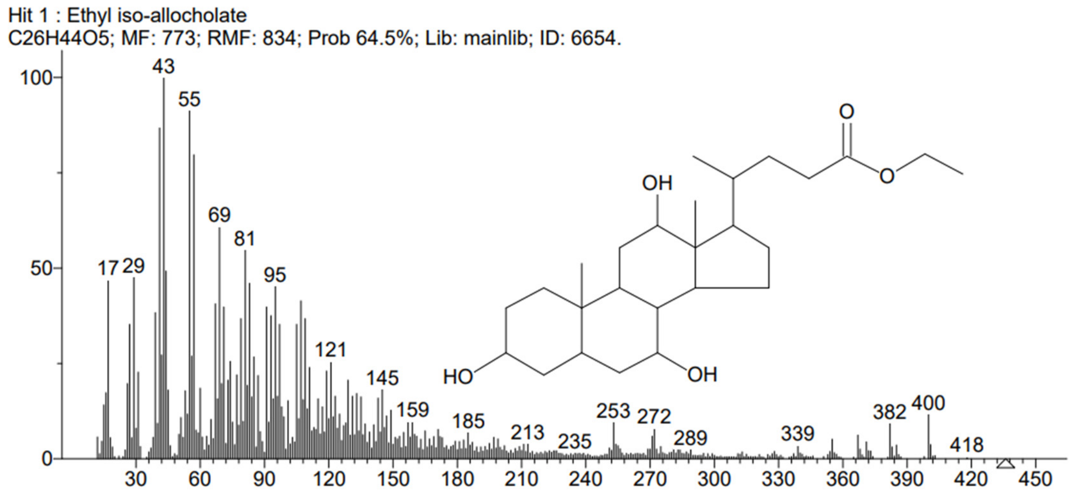

Figure S47. GC-MS profile of ethyl iso-allocholate

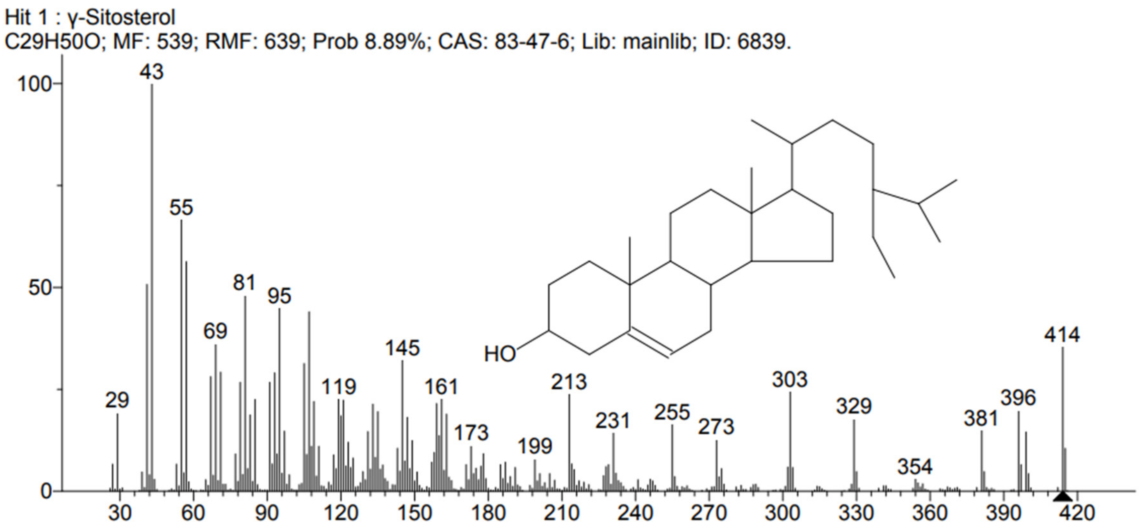

Figure S48. GC-MS profile of γ-sitosterol

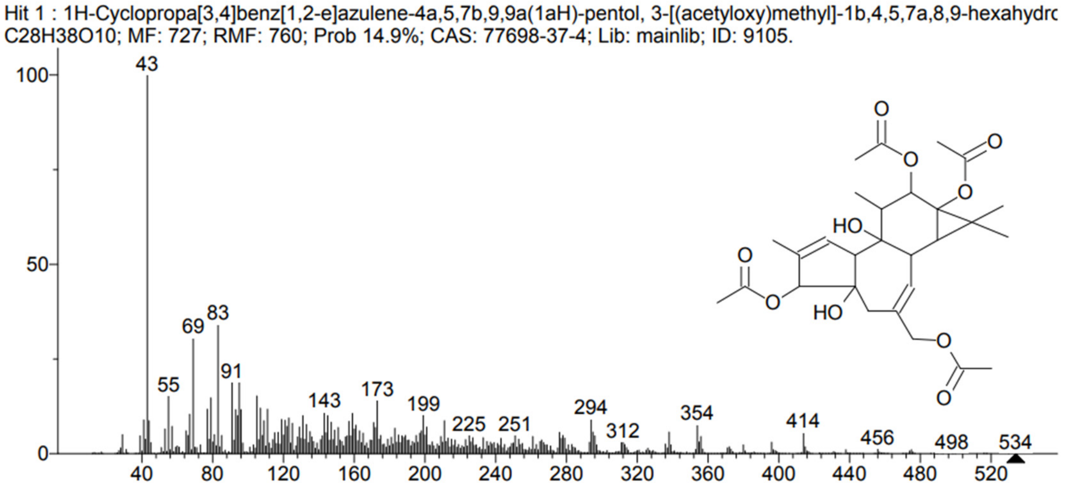

Figure S49. GC-MS profile of 1H-cyclopropa [3,4]benz[1,2-e]azulene-4a,5,7b,9,9a(1aH)-pentol

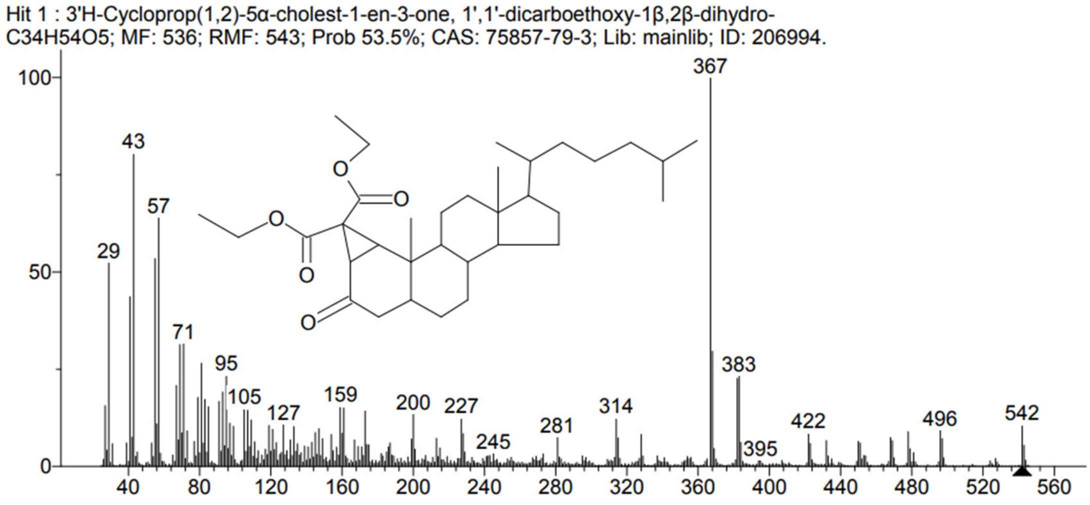

Figure S50. GC-MS profile of 1',1'-dicarboethoxy-1β,2β-dihydro-3'H-cycloprop(1,2)-5α-cholest-1-en-3-one

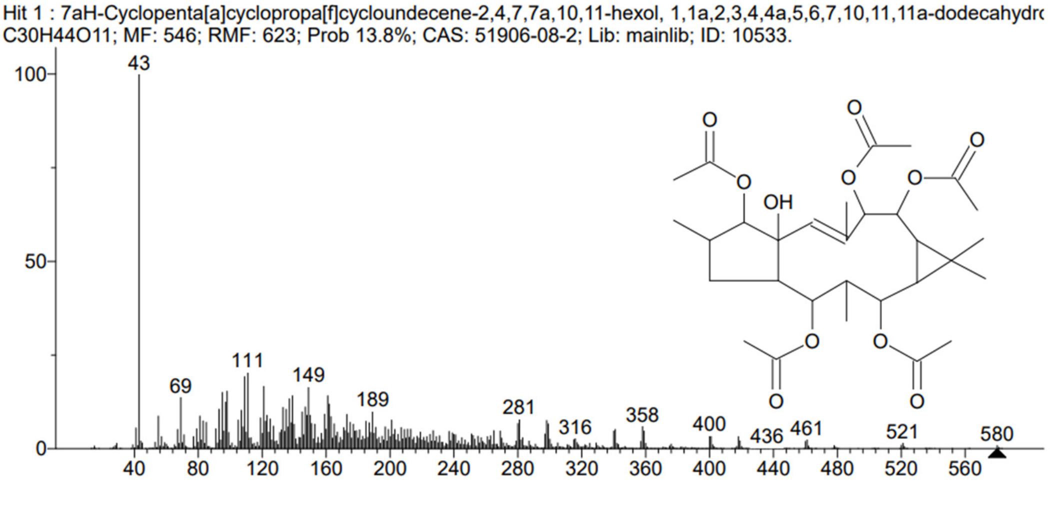

Figure S51. GC-MS profile of 7aH-cyclopenta[a]cyclopropa[f]cycloundecene-2,4,7,7a,10,11-hexol

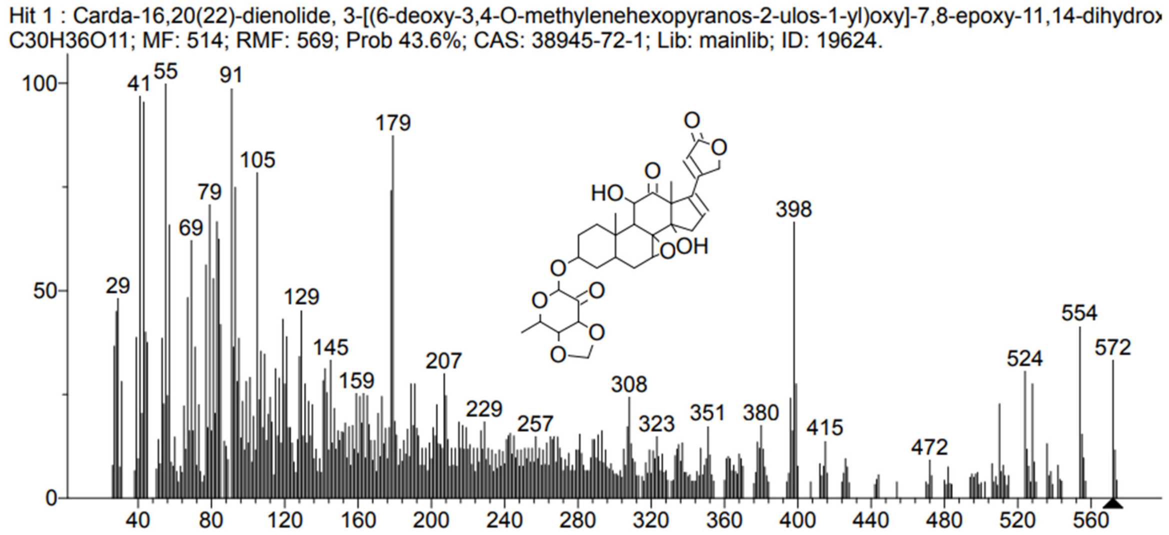

Figure S52. GC-MS profile of carda-16, 20(22)-dienolide

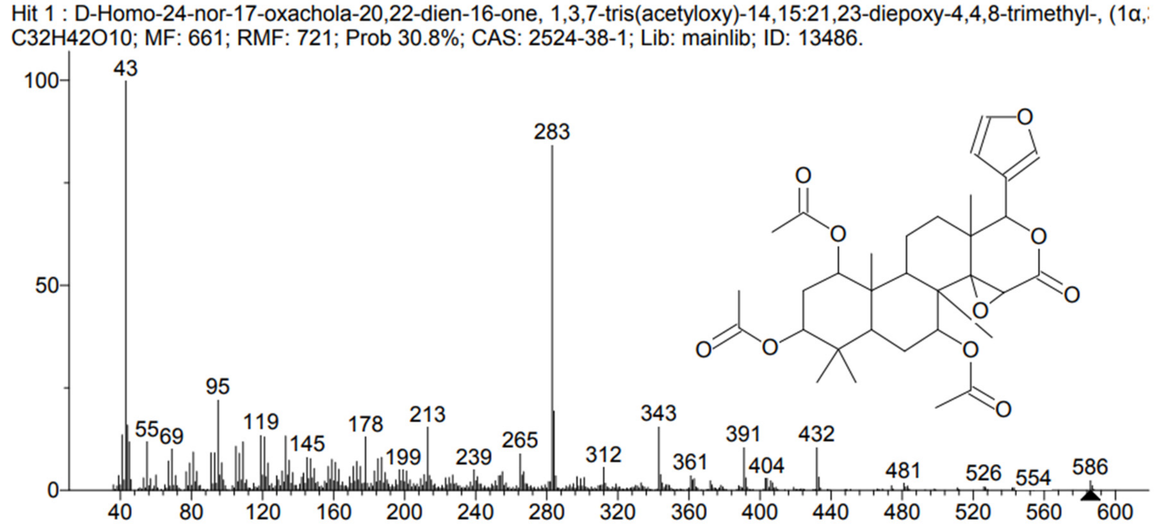

Figure S53. GC-MS profile of D-homo-24-nor-17-oxachola-20,22-dien16-one,

Peak @ 14.175 **Area** 3847898.772 **Area %** 2.44

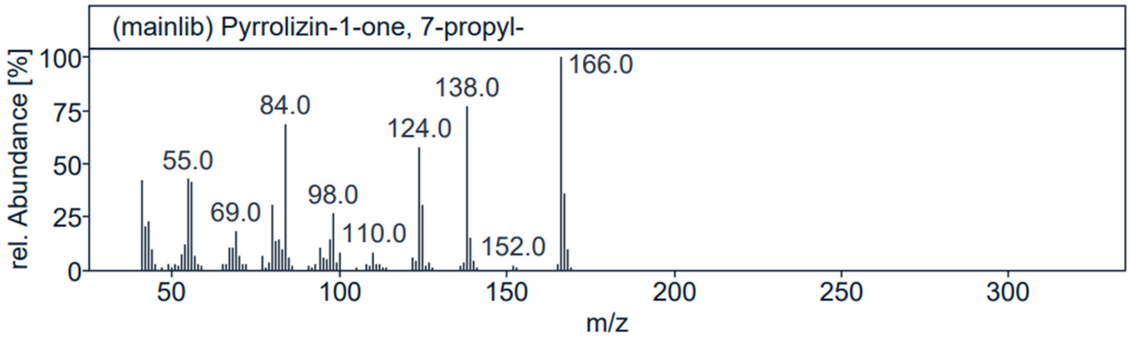

Figure S54. GC-MS profile of pyrrolizin-1-one, 7-propyl-

Peak @ 15.297 **Area** 1522001.090 **Area %** 0.97

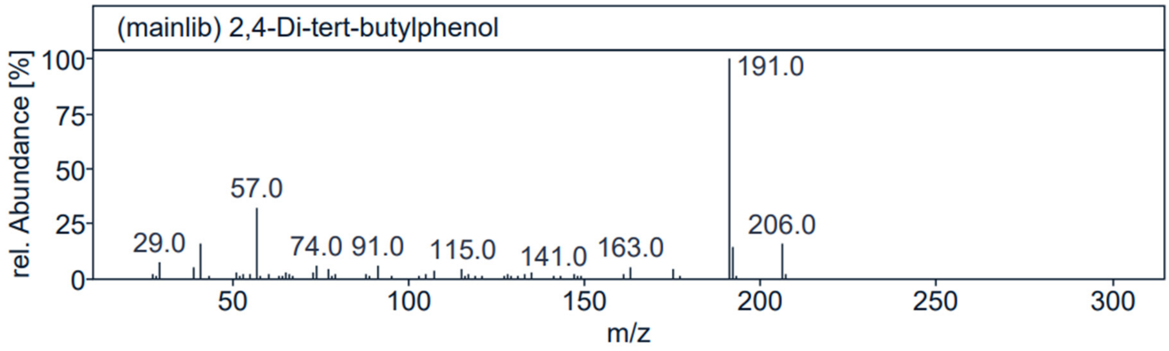

Figure S55. GC-MS profile of 2,4-di-tert-butylphenol

Peak @ 17.119 **Area** 1110247.260 **Area %** 0.71

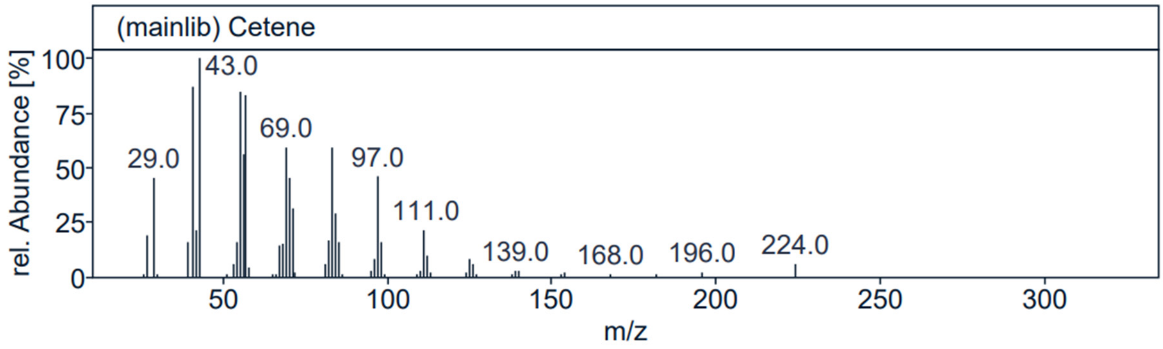

Figure S56. GC-MS profile of cetene

Peak @ 17.916 **Area** 1163961.481 **Area %** 0.74

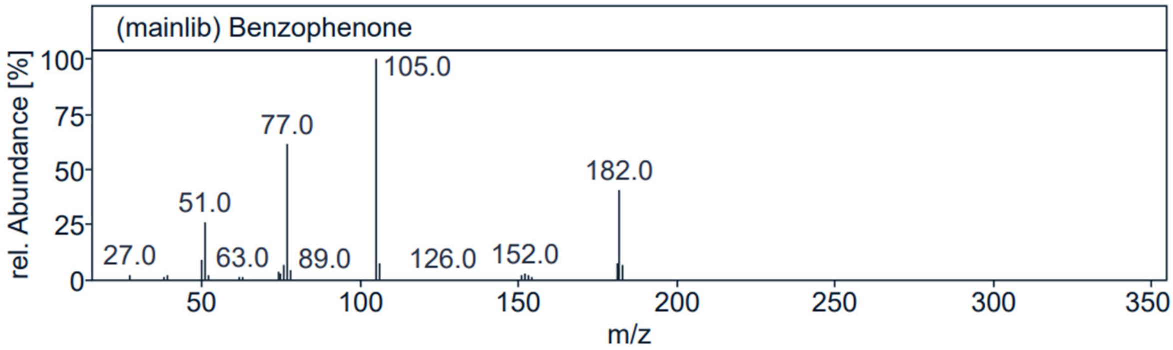

Figure S57. GC-MS profile of benzophenone

Peak @ 18.907 **Area** 672505.570 **Area %** 0.43

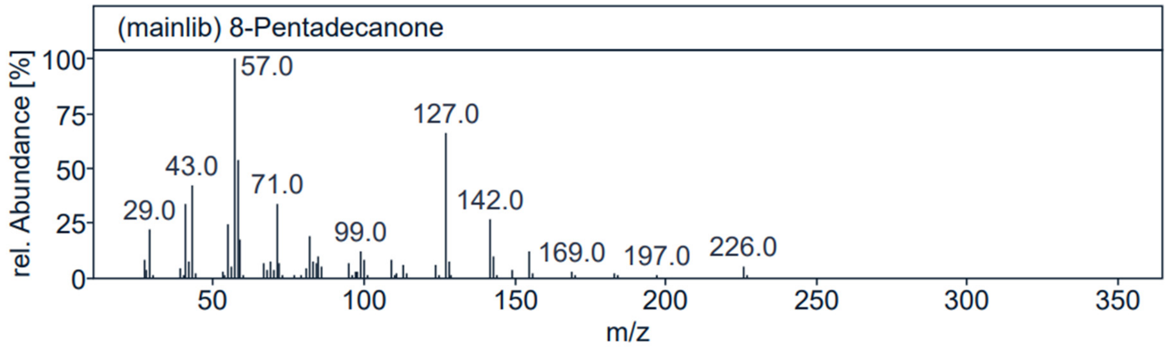

Figure S58. GC-MS profile of 8-pentadecanone

Peak @ 19.612 **Area** 2423495.493 **Area %** 1.54

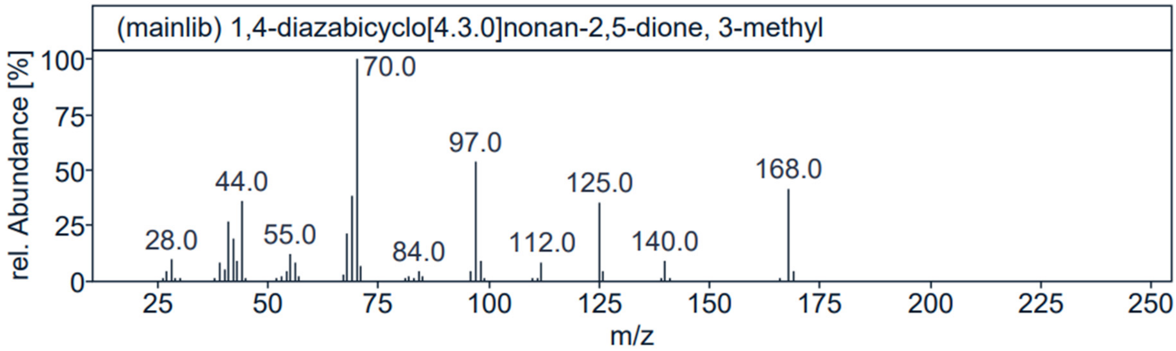

Figure S59. GC-MS profile of 3-methyl 1,4-diazabicyclo[4.3.0]nonan-2,5-dione

Peak @ 20.123 **Area** 1036006.759 **Area %** 0.66

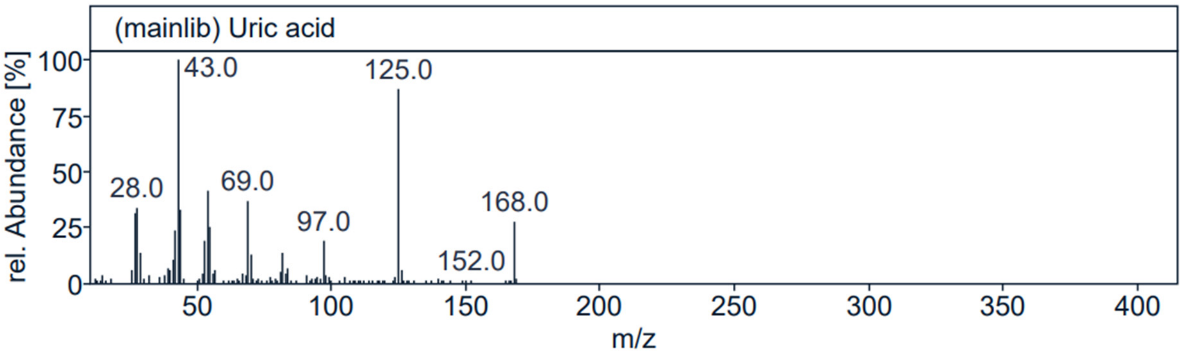

Figure S60. GC-MS profile of uric acid

Peak @	20.523	Area	2387316.925	Area %	1.52

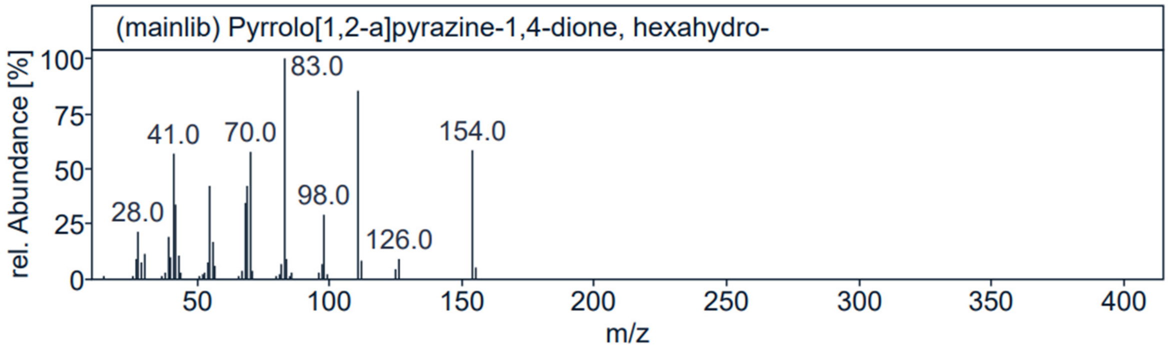

Figure S61. GC-MS profile of hexahydro-pyrrolo[1,2-a]pyrazine-1,4-dione

Peak @	20.894	Area	725630.506	Area %	0.46

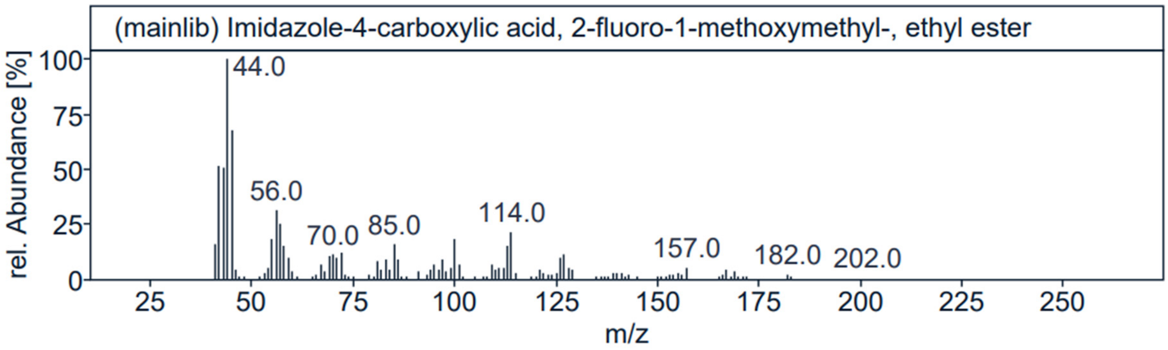

Figure S62. GC-MS profile of 2-fluoro-1-methoxymethyl-imidazole-4-carboxylic acid ethyl ester

Peak @	21.067	Area	714226.534	Area %	0.45

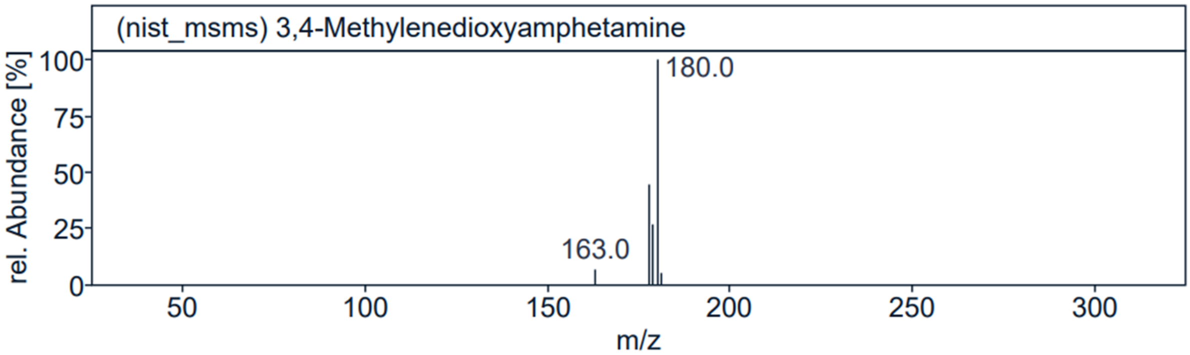

Figure S63. GC-MS profile of 3,4-methylenedioxyamphetamine

Peak @ 21.464 **Area** 2002932.891 **Area %** 1.27

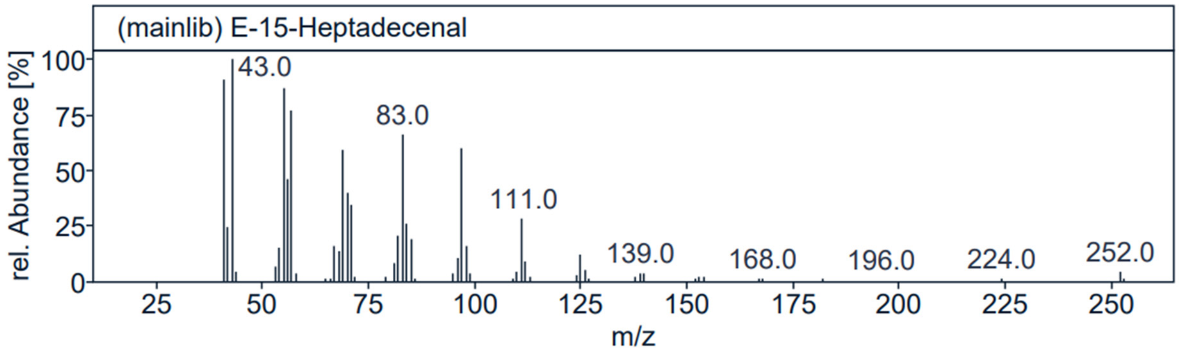

Figure S64. GC-MS profile of E-15-heptadecenal

Peak @ 21.944 **Area** 17604569.972 **Area %** 11.19

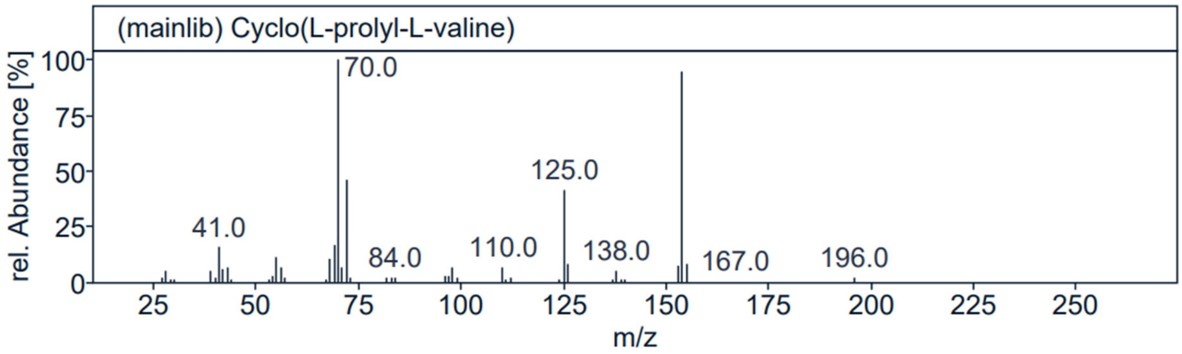

Figure S65. GC-MS profile of cyclo(L-prolyl-L-valine)

Peak @ 22.741 **Area** 3221313.785 **Area %** 2.05

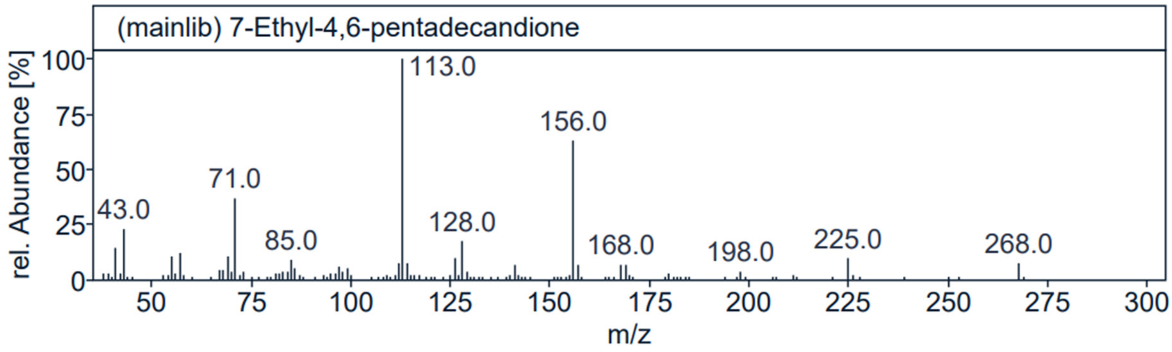

Figure S66. GC-MS profile of 7-ethylpentadecane-4,6-dione

Peak @ 23.350 Area 1398959.870 Area % 0.89

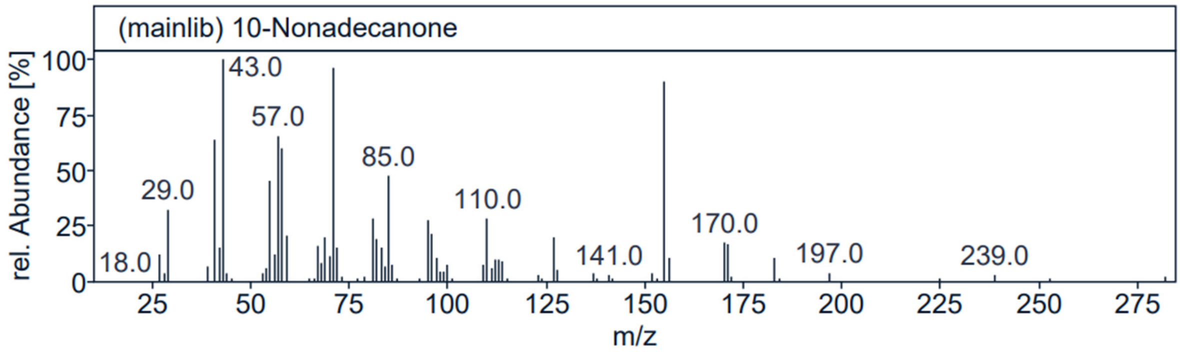

Figure S67. GC-MS profile of 10-nonadecanone

Peak @ 24.335 Area 11499146.143 Area % 7.31

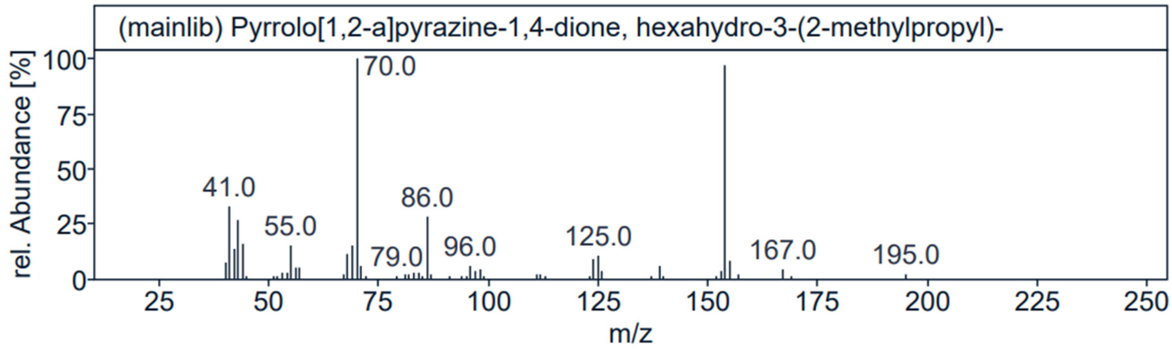

Figure S68. GC-MS profile of hexahydro-3-(2-methylpropyl)-pyrrolo[1,2-a]pyrazine-1,4-dione

Peak @ 25.831 Area 4493876.557 Area % 2.86

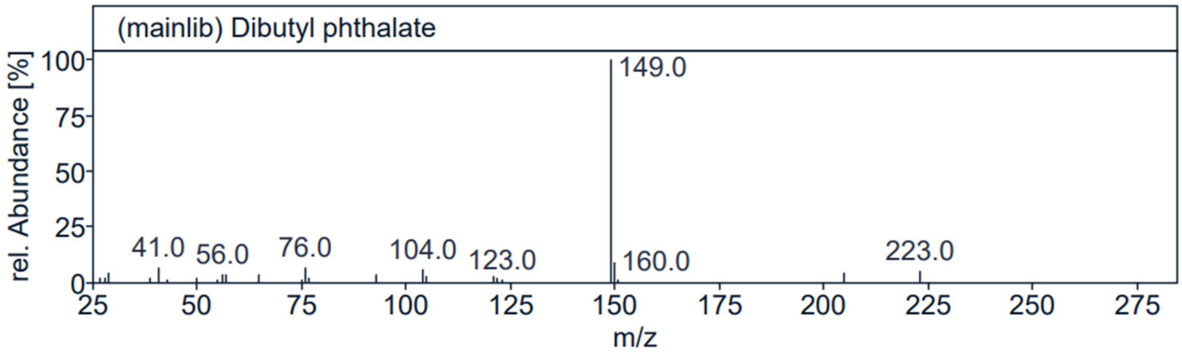

Figure S69. GC-MS profile of dibutyl phthalate

Peak @ 26.667 **Area** 1601921.630 **Area %** 1.02

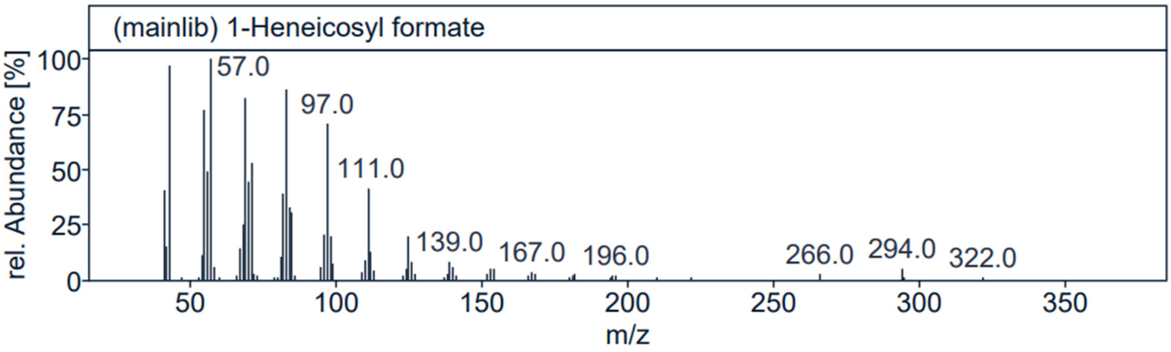

Figure S70. GC-MS profile of 1-heneicosyl formate

Peak @ 30.090 **Area** 2080323.497 **Area %** 1.32

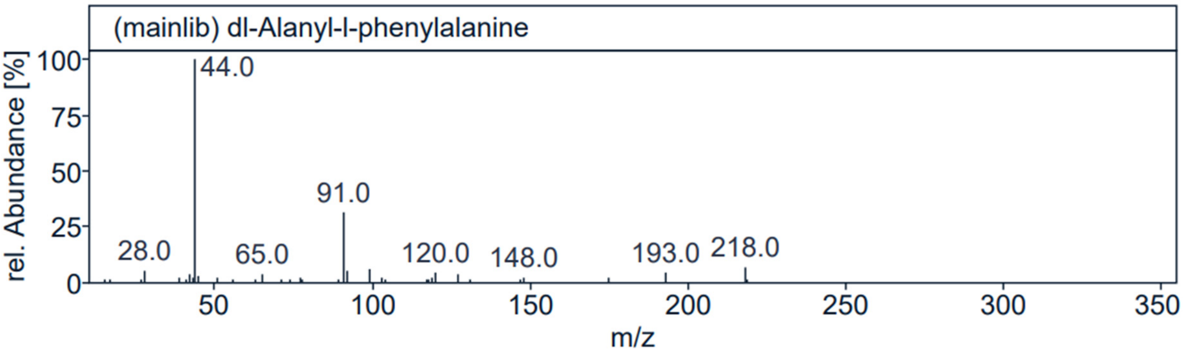

Figure S71. GC-MS profile of *dl*-alanyl-*dl*-phenylalanine

Peak @ 30.603 **Area** 6348293.375 **Area %** 4.03

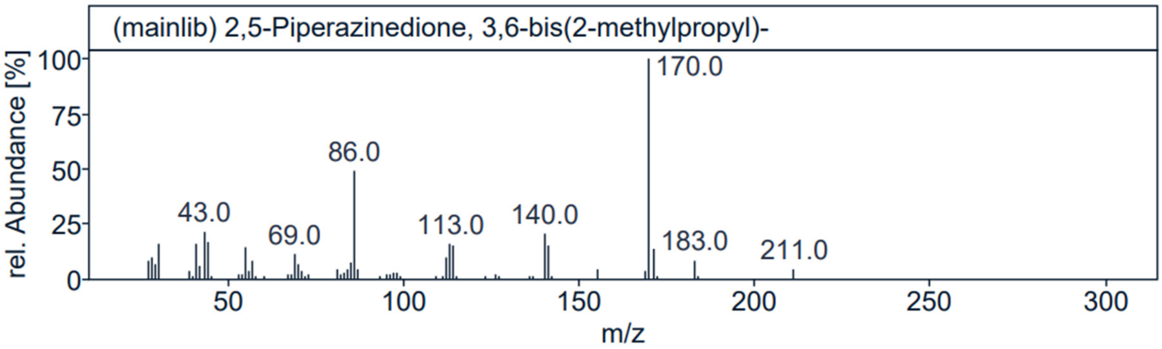

Figure S72. GC-MS profile of 3,6-bis(2-methylpropyl)-2,5-piperazinedione
